# Cooperative Brønsted Acid and Photo‐Promoted Stereoselective Synthesis of Substituted Piperidones

**DOI:** 10.1002/anie.202516050

**Published:** 2025-11-02

**Authors:** Qiupeng Peng, Poulami Mukherjee, Adhya Suresh, Meemie Hwang, Osvaldo Gutierrez, Karl A. Scheidt

**Affiliations:** ^1^ Department of Chemistry Northwestern University 2145 Sheridan Road Evanston IL 60208 USA; ^2^ Department of Chemistry and Biochemistry University of California, Los Angeles 607 Charles E. Young Drive East Los Angeles CA 90095 USA; ^3^ Department Chemistry School of Science China Pharmaceutical University 639 Longmian Dadao, Jiangning District Nanjing 211198 China

**Keywords:** Carbonyl triplet diradical, Chromophore activator, Cis‐piperidone and pyrrolidone, Formal ring‐insertion

## Abstract

Saturated nitrogen heterocycles are highly prevalent in medicinal chemistry, agrochemistry, and material science. Despite extensive research to access substituted piperidines and related compounds, there remains a strong demand for new stereoselective approaches. We present a Brønsted acid‐promoted, light‐induced formal ring‐insertion strategy for the efficient synthesis of piperidone and pyrrolidone derivatives. This method enables the efficient construction of thermodynamically disfavored *cis*‐disubstituted piperidone and pyrrolidone derivatives. A key aspect of this strategy is the use of an acyl imidazole as a uniquely capable chromophore activator, in cooperation with the Brønsted acid, which controls both reactivity and stereoselectivity without the need for photosensitizers. The unique properties of the in situ generated carbonyl triplet diradical enable a selective 1,5‐hydrogen atom transfer process, followed by C─N bond cleavage and a subsequent Mannich reaction, offering broad functional group tolerance. The synthetic utility of this methodology was exemplified by the preparation of key intermediates for the synthesis of neurokinin 1 antagonist drug candidates.

## Graphical abstract

A Brønsted acid cooperates with a photoexcited carbonyl triplet to promote stereoselective C─C bond formation in disubstituted piperidones through a chromophore‐activator strategy. The Brønsted acid lowers the excitation energy gap, directs selective C─N bond cleavage over C─C fragmentation after H‐atom transfer, and enables enantioselective C─C bond construction.

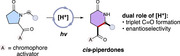

Piperidines and related 6‐membered saturated analogs are a major class of nitrogen‐containing heterocycles in organic chemistry.^[^ ^1^, ^2^ ^]^ They are also among the most prevalent structures found in pharmaceuticals and bioactive molecules (Figure 1a).^[^ ^3^, ^4^, ^5^, ^6^ ^]^ Consequently, significant effort has been expended to develop efficient synthetic methods to access piperidines and piperidones, which can be broadly categorized into three distinct strategies (Figure 1b).^[^ ^7^ ^]^ A straightforward approach involves the hydrogenation of substituted pyridines, which yields *cis*‐substituted piperidines.^[^ ^8^, ^9^, ^10^, ^11^, ^12^ ^]^ However, achieving enantioselectivity in these reactions can be challenging, usually requiring transition metal‐chiral ligand catalysts.^[^ ^13^ ^]^ Another strategy involves convergent reactions such as formal [4 + 2],^[^ ^14^, ^15^ ^]^ [3 + 3],^[^ ^16^ ^]^ and [5 + 1]^[^ ^17^ ^]^ cycloadditions. These cycloaddition methods typically produce thermodynamically favored *trans*‐substituted piperidines.^[^ ^18^ ^]^ Intramolecular S*_N_* 2 or acylation reactions offer a different alternative for constructing piperidine/ones with well‐defined substitution patterns from elaborated starting materials.^[^ ^19^, ^20^, ^21^, ^22^, ^23^, ^24^ ^]^ The desymmetrization of meso‐piperidine was shown to be a feasible pathway with carefully refined substrates.^[^ ^25^ ^]^ Despite the utility of these synthetic strategies, few offer the ability to efficiently access stereochemical permutations of substituted piperidine/piperidones with high levels of stereocontrol from a common chiral intermediate.^[^ ^26^ ^]^

**Figure 1:**
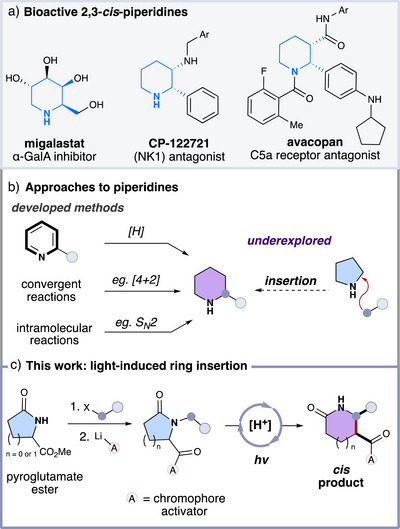
a) Representative examples of bioactive molecules containing *cis*‐disubstituted piperidine scaffolds. b) Approaches to piperidines. c) Reaction design.

Molecular remodeling, or skeletal editing, wherein an atom or functional group change can dramatically modulate physical properties, has long been appreciated in organic chemistry and has recently experienced an impressive and exciting renaissance.^[^ ^27^, ^28^ ^]^ The venerable Büchner reaction and recent reports by Levin,^[^ ^29^ ^]^ Morandi,^[^ ^30^, ^31^ ^]^ and others^[^ ^32^, ^33^, ^34^, ^35^, ^36^, ^37^ ^]^ have creatively demonstrated efficient and unique pathways to access nitrogen heteroarenes via ring insertion processes involving carbenes or nitrenes.

Our interest in molecular remodeling has been stimulated by light‐induced carbonyl triplet chemistry, utilizing both efficient “activators” to access synthetically useful high‐energy intermediates and modern photonic energy sources.^[^ ^38^, ^39^, ^40^, ^41^, ^42^, ^43^ ^]^ We hypothesized that an attractive molecular remodeling approach could leverage triplet chemistry to forge fully saturated substituted six‐membered piperidines from five‐membered pyrrolidines (Figure 1c). The reaction design involves straightforward *N*–alkylation followed by the installation of a specifically optimized chromophore activator.^[^ ^44^, ^45^ ^]^ The subsequent exposure of these systems to light in the presence of a Brønsted acid could promote a formal ring insertion involving a site‐selective hydrogen atom transfer process, yielding non‐thermodynamically favored 2,3‐*cis*‐substituted piperidones.^[^ ^46^ ^]^

In 1971, Leermakers reported an intriguing study demonstrating that Brønsted acids could modulate the carbonyl groups in ketones or aldehydes, promoting a transition from the ground state to the triplet excited state and extending the lifetime of resulting triplet diradicals (Figure 2a).^[^ ^47^, ^48^ ^]^ However, this discovery has to the best of our knowledge not yet been applied in HAT chemistry toward new transformations. Although the role of Brønsted acids in facilitating reactions involving carbon–carbon double bonds in unsaturated compounds has been explored by Yoon,^[^ ^49^ ^]^ Bach,^[^ ^50^ ^]^ and others,^[^ ^51^, ^52^ ^]^ the activation of carbonyl groups in ester surrogates remains less explored.^[^ ^53^, ^54^, ^55^, ^56^, ^57^, ^58^ ^]^ This synthetic gap provided an opportunity to develop a novel activation model specifically targeting the excitation of carbonyl groups in esters and their oxidative equivalents.

**Figure 2:**
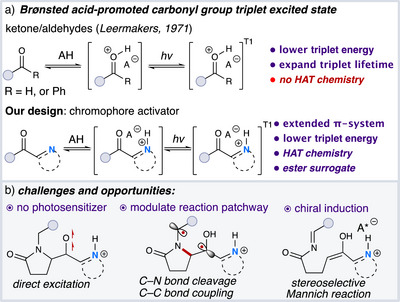
Brønsted acid promoted photochemistry.

Achieving a formal 5‐ to 6‐membered ring insertion (Figure 2b), requires addressing several key challenges. First, the selection of a chromophore activator that can be easily installed and enable direct photoexcitation of the carbonyl group without the need for an exogenous photosensitizer to achieve a triplet diradical species. Second, we anticipated challenges controlling the selectivity of C─N bond cleavage to generate either an imine and enol intermediate, or C─C bond coupling to form a four‐membered ring after the 1,4‐diradical intermediate is formed.^[^ ^59^ ^]^ Third, achieving high levels of enantioselectivity and diastereoselectivity of the subsequent Mannich reaction would be another significant hurdle. Last, ensuring that the Brønsted acid selectively interacts with the chromophore activator without compromising the reactivity of the carbonyl group could be an obstacle.^[^ ^60^ ^]^

We initiated our study by employing *N*‐methyl imidazole as a chromophore activator with diphenyl hydrogen phosphate as a Brønsted acid in dichloromethane and irradiation with 390 nm LEDs (light‐emitting diodes). With these conditions, the desired ring‐insertion product **2a** was obtained in 38% yield (Table 1, entry 1). Upon evaluating various solvents, we determined that acetonitrile was the optimal solvent and provided the desired product in improved yield (Table 1, entries 2–7). Switching to 370 nm LEDs increased the yield to 62% (Table 1, entry 8), while other light sources (427 or 456 nm LEDs) resulted in only trace amounts of the product or no reaction (Table 1, entries 12–13). When the loading of the Brønsted acid was reduced, a decrease in yield was observed, accompanied by the formation of an unidentified side product (Table 1, entries 9–11). Control experiments confirmed that light is essential for the reaction, as no product was obtained in the absence of light. The addition of a free radical scavenger, TEMPO, completely inhibited the reaction, further supporting the involvement of a radical process. Furthermore, conducting the reaction under air resulted in a significant decrease in yield (6%), suggesting the formation of triplet diradical species under standard conditions. The reaction also proved sensitive to moisture, as only 10% of the product was obtained when water was introduced under standard conditions.

**Table 1:** Reaction optimization^a)^. [Correction added on November 9, 2025, after first online publication: Table 1 has been updated.]

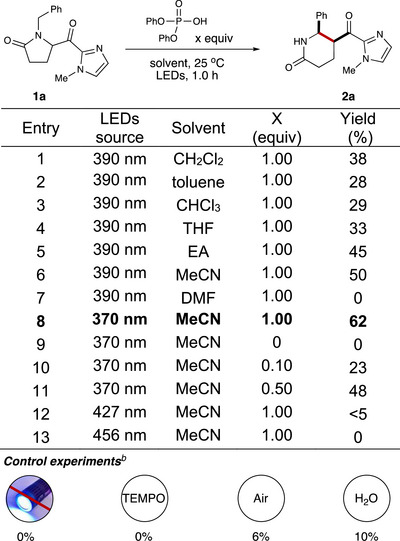				

^a)^ Reaction conditions: **1a** (0.10 mmol) scale, yield determined by ^1^H NMR using 1,3,5‐trimethoxybenzene as internal standard, diastereomeric ratios determined by ^1^H NMR analysis of unpurified reaction mixtures (in all cases > 20:1 d.r.), Kessil LEDs lamp (44 W), N_2_, 25 °C.

^b)^ Control experiments: **1a** (0.10 mmol), diphenyl hydrogen phosphate (0.10 mmol); no irradiation; 2.0 equivs of TEMPO; under air atmosphere; 10.0 equivs of water were added.

With the optimized conditions established, we proceeded to explore the substrate scope. Various *N*‐substituted chromophore activators were tested, all successfully undergoing the desired transformation with acceptable yields (Figures 3 and 2a–d). Both electron‐withdrawing and electron‐donating groups at the para position of the phenyl ring were incorporated under standard conditions without a significant decrease in yield (Figures 3 and e–j). Notably, electron‐donating groups provided slightly better yields and shorter reaction times, likely due to the lower bond dissociation energy (BDE) of the C─H bond at the benzylic position.^[^ ^61^ ^]^ The *cis*‐substituted relationship of the product was confirmed by the X‐ray crystallography for compound **2i**.^[^ ^62^ ^]^ We tested methyl‐ and chloride‐substitution at the meta position, which delivered the target products in satisfactory yields (Figures 3 and 2k,l). The more sterically hindered ortho‐methyl‐substituted derivative **2m** was obtained with 42% yield. Similarly, the naphthalene‐substituted product **2n** was formed in 40% yield. To further examine the applicability of this strategy in late‐stage functionalization, we applied our strategy to ataluren (treat Duchenne muscular dystrophy) derivative **2o**, obtaining a 34% yield (64% brsm). Additionally, we extended the reaction to explore the ring insertion from a β‐lactam to a γ‐lactam, and were pleased to observe that product **2p** was achieved with 71% yield. Bromide‐ and methyl‐substituted phenyl rings were also tested, and the desired products were obtained in moderate to good yields (Figures 3 and 2q,r). Despite full consumption of proline, indoline, or *N*‐methyl/propargyl substrates, no identifiable products were obtained. Attempts to expand the six‐membered ring to a seven‐membered ring were unsuccessful, as the formation of the seven‐membered ring is thermodynamically unfavorable.

**Figure 3:**
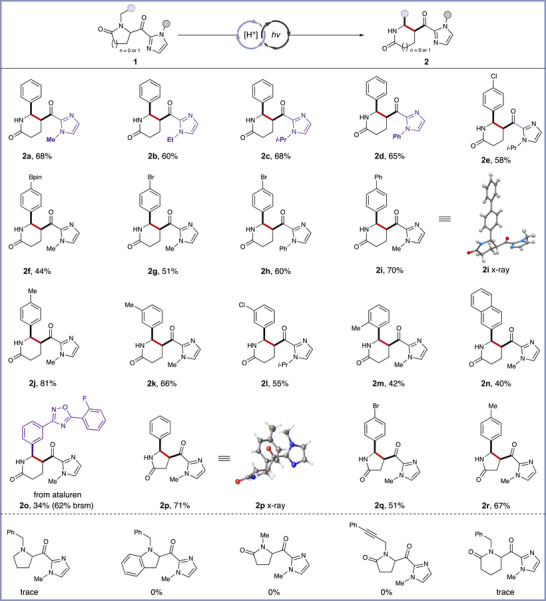
Substrate scope^a)^. ^a)^Standard conditions: **1** (0.20 mmol), diphenyl hydrogen phosphate (0.20 mmol), MeCN (2.0 mL), Kessil 370 nm LED lamp (44 W), N_2_, 1 – 16 h, isolated yield reported (for more details, see Supporting Information), all *d.r*. >20:1.

We next focused on the developing the chiral Brønsted acid catalyzed enantioselective synthesis of chiral piperidones (Figure 4). We were pleased to find that the *N*‐phenyl substituted acyl imidazole was stable under the irradiation conditions without decomposition for 24 h. Introducing 10 mol % of *R*‐TRIP (3,3′‐Bis(2,4,6‐triisopropylphenyl)‐1,1′‐binaphthyl‐2,2′‐diyl hydrogenphosphate) in 1,4‐dioxane (0.1 M) and irradiating under 370 nm LEDs for 48 hresulted in the formation of enantioenriched *cis‐(5S, 6R)‐* **2d** with excellent enantiomeric excess (90% *ee*) and diastereomeric ratio (> 20:1 d.r.). Subsequently, heating *cis‐(5S, 6R)‐* **2d** in isopropanol at 100 °C for 48 h led to the thermodynamically favored *trans‐(5R, 6R)‐* **2d** in good yield, high *ee*, and *d.r*. When *S*‐TRIP was applied under the same conditions, the *cis‐(5R, 6S)‐* **2d** was smoothly produced with high *ee* and *d.r*. The thermodynamically favored *trans‐(5R, 6R)‐* **2d** could also be obtained from *cis‐(5R, 6S)‐* **2d** in good yield when heated in isopropanol. Notably, all stereoisomers of the product can be accessed through these straightforward protocols.

**Figure 4:**
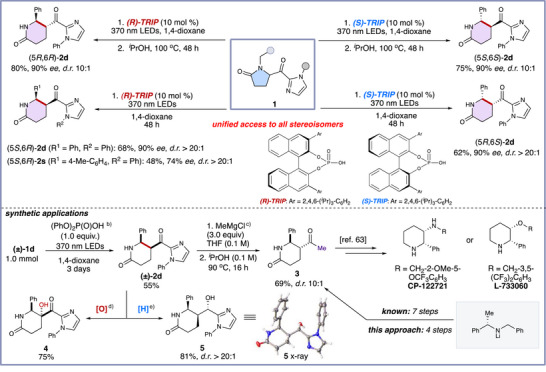
Asymmetric synthesis of piperidones and synthetic applications^a)^. ^a)^Standard conditions: **1d** (0.20 mmol), *R*‐TRIP (0.020 mmol, 10 mol %), 1,4‐dioxane (2.0 mL), Kessil 370 nm LED lamp (44 W), N_2_, 48 h, isolated yield reported, *ee* was determined by chiral SFC, *d.r*. diastereomeric ratios determined by ^1^H NMR spectroscopic analysis of unpurified reaction mixtures (for more details, see Supporting Information). ^b)^ **1d**, 1.0 mmol scale, 3 days. ^c)^ (±)‐**2d** (0.2 mmol, 1.0 equiv. MeMgCl (3.0 equiv.), THF (0.1 M), 2.0 h; isopropanol (0.1 M), 24 h. ^d)^ (±)‐**2d** (0.1 mmol, 1.0 equiv.), Na_2_HPO_4_ (10.0 equiv.), UPH (12.0 equiv.), TFAA (4.0 equiv.), DCM (0.1 M), 40 °C, 16 h. ^e)^ (*5S,6R*)‐**2d** (0.1 mmol, 1.0 equiv.) was used, NaBH_4_ (1.0 equiv.), THF/MeOH (10/1, 0.1 M), 4 h, 25 °C

We next increased the scale of the process to demonstrate the robustness of this protocol. On a 1.0 mmol scale, we successfully obtained **2d** in 55% yield. To further explore the utility of this method, **2d** was treated with MeMgCl followed by heating to 90 °C to form the methyl ketone in a single‐flask procedure, yielding **3** with 69% yield in two steps which can be synthesized in six steps according to the literature.^[^ ^63^, ^64^ ^]^ Notably, **3** is the key intermediate in the synthesis of the neurokinin 1 antagonist drug candidates CP‐122721 and L‐733060. Additionally, **2d** can be treated with either an oxidant or reductant to yield products **4** or **5** in good yields, respectively. The absolute configuration of **5** was determined to be (*5S, 6R*) by X‐ray crystallography when (*5S,6R*)‐**2d** was utilized (for more details, see Supporting Information Table S3), and other products were assigned by analogy.

The interaction between substrate **1a** and Brønsted acid (PO(OPh)_2_OH) was analyzed by ^1^H‐NMR spectroscopy. As the equivalents of Brønsted acid are increased, the protons H_a_ exhibit downfield shifts, while H_b_ and H_c_ undergo upfield shifts (Figure 5a). However, no bathochromic shift was observed in the absorbance profile after the addition of the Brønsted acid (see Supporting Information Figure S7 for more details). Hence, we postulated that the addition of Brønsted acid decreases the LUMO of carbonyl group which has been further supported by following dispersion‐corrected density functional theory (DFT, see below). Light on‐off experiments further demonstrated that a radical chain process is less favored under the standard conditions (see Supporting Information Figure S8 for more details). The chromophore activator, in cooperation with the Brønsted acid, plays a ***crucial role*** in this transformation, as evidenced by the absence of product when ester, phenyl ketone, pyridyl ketone, or acyl phosphonate were employed (Figure 5b). This observation interestingly contrasts with Sarpong's elegant previous work on a related ring contraction, which proceeds without the need for a Brønsted acid.^[^ ^65^, ^66^ ^]^ Additionally, non‐linear effects were observed, suggesting that multiple Brønsted acid catalysts are involved in the process (Figure 5c). Finally, kinetic isotope effect (KIE) experiments indicated that the 1,5‐HAT step is unlikely to be rate‐determining in this process (Figure 5d).

**Figure 5:**
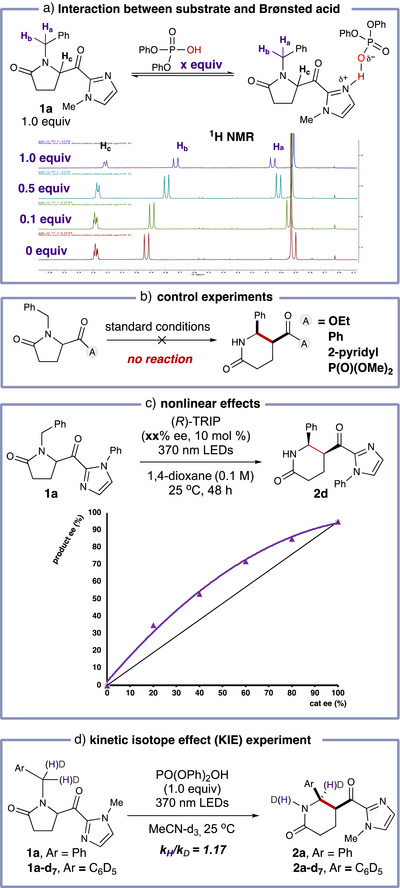
Mechanism study^a)^. ^a)^ **1a** (0.10 mmol), diphenyl hydrogen phosphate (x equiv.), MeCN‐d_3_ (0.1 M). ^b)^ **1a** (0.10 mmol), or **1a‐d_7_** (0.10 mmol), diphenyl hydrogen phosphate (1.0 equiv.), MeCN‐d_3_ (0.1 M). For more details, see Supporting Information.

To deepen our understanding of the unusual role of the Brønsted acid and the origin of diastereoselectivity in this process, we turned to dispersion‐corrected DFT calculations (see Supporting Information for additional details). Figure 6 presents the computed energy profile for the protonated *N*‐methyl imidazole chromophore activator (**1aH^+^**). The first step of the mechanism is presumably the excitation of **1aH^+^** under 370‐nm‐wavelength light irradiation followed by intersystem crossing (ISC) to reach the triplet excited state ^3^[1aH^+^]*.^[^ ^39^ ^]^ **1aH^+^** generates the triplet excited state ^3^[1aH]^+^* with a relatively lower energy of excitation compared to the non‐protonated **1a (**Δ*E* = 55.1 kcal mol^−1^ versus 66.2 kcal mol^−1^ respectively, see Figures S10 and S11) due to the decreased excitation energy gap between HOMO and LUMO on protonation of the chromophore.^[^ ^49^ ^]^ Mulliken spin density analysis of optimized **^3^[1aH]^+^** * revealed that the spin density was primarily located on the oxygen atom (as shown in red) consistent with selective O─H bond formation via HAT step (versus C─H bond formation initiated from acyl carbon). Moreover, the triplet species ^3^ **[1aH] ^+^** * is more efficient to undergo 1,5‐HAT with a relatively smaller energy barrier of 4.1 kcal mol^−1^ in contrast to that of the non‐protonated congener to deliver 1,4‐diradical **^3^BH^+^** (–13.8 kcal mol^−1^ downhill in energy from the excited state intermediate ^3^ **[1aH] ^+^** *).

**Figure 6:**
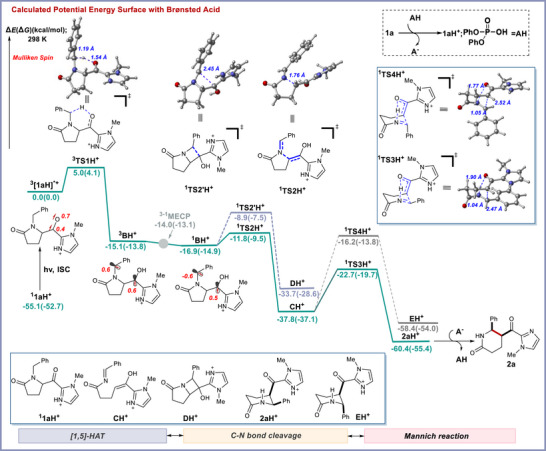
DFT calculations. Working Mechanism supported by computational studies. Calculated energies [uB3LYP‐D3/def2‐svp‐CPCM(ACN)] are in kcal mol^−1^.

Furthermore, consistent with the experimental findings **^3^BH^+^** can also convert to open shell singlet species **^1^BH^+^** via minimum energy crossing point (MECP, Δ*E *= 1.1 kcal mol^−1^), which in turn promotes selective C─N bond fragmentation over C─C bond formation. Specifically, as illustrated in Figure 6, **^1^BH^+^** can undergo C─N bond cleavage with a relatively smaller energy barrier of 5.4 kcal/mol via **^1^TS2H^+^** (versus 7.4 kcal mol^−1^ for C─C bond formation via **^1^TS2’H^+^** emanating from the radical‐radical coupling) to afford the more thermodynamically favored imine enol intermediate species **CH^+^** (downhill by 37.1 kcal mol^−1^ in energy). Notably, the shorter C─N bond length observed in the protonated transition state **^1^TS2H^+^** compared to its non‐protonated counterpart (1.76 versus 1.84 Å, respectively; Figures S11 and S10) indicates a more facile C─N bond cleavage pathway that leads to a lower energy barrier. *These data highlight the critical role of protonation in facilitating this transformation*. In the next step, the imine enol intermediate **CH^+^** performs an irreversible Mannich reaction via a six‐membered chair‐like transition state **^1^TS3H^+^ (**Δ*G* ^‡^ = 17.4 kcal mol^−1^) to afford the product intermediate **2aH** ^+^(–55.4 kcal mol^−1^ downhill by energy from the excited intermediate). Finally, the conjugate base can abstract the proton from the intermediate product to deliver the desired **2a**.

To shed light on the origin of *syn* versus *anti* diastereoselectivity in the final Mannich step, we explored the possible isomers of the imine enol intermediate **CH^+^** which could lead to *syn* and *anti*‐diastereomers (For details see Supporting Information, Figure S15). Among them, two transition states ^1^ **TS3H^+^** and ^1^ **TS4H^+^** were found to be stabilized by internal hydrogen bonding (C═N─H─O) via six membered rings which could lead to the *syn* and *anti*‐products respectively. However, ^1^ **TS3H^+^** exhibits a lower energy barrier (ΔΔ*G* ^‡^ = 5.9 kcal mol^−1^) compared to **^1^TS4H^+^** which can be attributed to the anti‐geometry of the imine double bond, enabling a stabilizing π‐π interaction between the protonated imidazole and the equatorially positioned Ph ring, as revealed by non‐covalent interaction analysis (see Supporting Information, Figure S16).^[^ ^67^, ^68^ ^]^ Therefore, we ascribe the origin of *syn* diastereoselectivity of the final product stemming from the combined effects of *anti‐*geometry of the imine double bond and stabilizing π‐π stacking. DFT calculations to investigate the absolute stereochemistry of (*5R,6S*)‐**2d** in the (*R*)‐TRIP‐catalyzed Mannich reaction require significant computational resources and are currently ongoing in our laboratory.^[^ ^69^, ^70^, ^71^ ^]^

We have developed a versatile ring‐insertion protocol for the concise synthesis of the full stereochemical set of 2,3‐*cis*‐substituted piperidones from simple racemic γ‐lactams. In contrast to previous related transformations, the unusual reactivity enabled by a specific chromophore activator and Brønsted acid allows for direct excitation of the carbonyl group, thereby generating a triplet diradical. This high‐energy intermediate then undergoes a selective 1,5‐HAT process that initiates C─N bond cleavage resulting in facile 5‐membered ring scission. In addition to providing access to triplet carbonyl state reactivity, the Brønsted acid is also involved in effectively controlling the enantio‐ and diastereoselectivity of the subsequent intramolecular Mannich reaction of the ring‐opened intermediate. Further applications and detailed mechanistic studies of light‐induced triplet‐state carbonyl chemistry are underway and will be reported in due course.

## Conflict of Interests

The authors declare no conflict of interest.

## Supporting information



Supporting Information

Supporting Information

## Data Availability

The data that support the findings of this study are available from the corresponding author upon reasonable request.
