# Supplementary material for: Cooperative Brønsted Acid and Photo‐Promoted Stereoselective Synthesis of Substituted Piperidones
Source: Angew Chem Int Ed Engl. 2025 Nov 2;64(51):e16050. doi: 10.1002/anie.202516050 (PMC12707350; doi:10.1002/anie.202516050)
Supplement: Supplementary file 1 — Supporting Information [file ANIE-64-e16050-s001.docx]

**Supporting Information**

Table of Contents

[General information 2](#_Toc182571866)

[Procedure for substrate synthesis 3](#_Toc182571867)

[Procedure I: synthesis of acyl imidazole 3](#_Toc182571868)

[Procedure II: procedure for light-induced ring insertion 11](#_Toc182571869)

[Procedure for asymmetric synthesis of piperidones 20](#_Toc182571870)

[Transformation 20](#_Toc182571871)

[TEMPO-trapping experiment 22](#_Toc182571872)

[O_2_-quench experiment 22](#_Toc182571873)

[H_2_O-quench experiment 23](#_Toc182571874)

[UV/Vis data 23](#_Toc182571875)

[Parallel KIE 24](#_Toc182571876)

[Light off and on experiment 24](#_Toc182571877)

[Nonlinear effects 25](#_Toc182571878)

[Crystal structure 26](#_Toc182571879)

[Computational methods 29](#_Toc182571880)

[Cartesian coordinates 34](#_Toc182571881)

[NMR Spectra 69](#_Toc182571882)

[SFC Spectra 110](#_Toc182571883)

[References 111](#_Toc182571884)

# General Information

All reactions were carried out under an argon or nitrogen atmosphere in oven-dried glassware with magnetic stirring. All solvents were purified by passing through a bed of activated alumina, dried over 3Å molecular sieves, and then degassed using freeze-pump-thaw method (3-4 cycles). Purification of reaction products was carried out by flash chromatography on Biotage Isolera 4 or Biotage Dalton 2000 (ELSD-A120) systems with ultra-grade silica cartridges. Analytical thin layer chromatography was performed on EM Reagent 0.25 mm silica gel 60-F plates. Visualization was accomplished with UV light or ceric ammonium molybdate (CAM) stain followed by heating. ^1^H NMR spectra were recorded on an AVANCE III 500 MHz w/ direct cryoprobe (500 MHz) spectrometer and are reported in ppm using solvent as an internal standard (CDCl_3_ at 7.26 ppm). Data are reported as (ap = apparent, s = singlet, d = doublet, t = apparent triplet, q = quartet, m = multiplet; coupling constant(s) in Hz; integration) Proton-decoupled ^13^C NMR spectra were recorded on an AVANCE III 500 MHz w/ direct cryoprobe (126 MHz) spectrometer and are reported in ppm using solvent as an internal standard (CDCl_3_ at 77.16 ppm). Mass spectra were obtained on a WATERS Acquity-H UPLC-MS with a single quad detector (ESI) or an Agilent 7890 gas chromatograph equipped with a 5975C single quadrupole EI-MS. High-resolution mass spectrometry (HRMS) was obtained using an Agilent 6201 MSLC-TOF (ESI) or Bruker IMPACT II (ESI). Enantioselectivity measurements were made on an Agilent 1290 Infinity SFC using Chiralpak IA-3, IB-3, IC-3, ID-3, IG-3 chiral stationary phases.

# Procedure for substrate synthesis

# Procedure I: synthesis of acyl imidazole

All reactions were set up under N_2_ atmosphere. To an oven-dried flask containing a stir bar, imidazole (12.0 mmol, 1.2 equiv) and THF (50 mL) were added at -78 ^o^C, then *n*-BuLi (12.0 mmol, 1.2 equiv, 1.6 M in hexane) was added dropwise. The reaction was stirred at the same temperature for 0.5 h before **s1** (10.0 mmol, 1.0 equiv) was added dropwise. The mixture was stirred for 2.0 h and monitored by TLC and UPLC-MS. When complete consumption of **s1** was observed, then quenched with NH_4_Cl (aq.) and partitioned between brine (50 mL) and ethyl acetate (100 mL). The layers were separated, and the aqueous layer extracted with ethyl acetate (2x40 mL), and the organic layers combined. The organic layers were washed with saturated sodium bicarbonate solution (40 mL) and brine (40 mL), then dried with Na_2_SO_4_, filtered, and concentrated. The residue was purified by silica gel column chromatography (acetone/hexane 0-40%) to give the corresponding acyl imidazole **1**.^[72]^

*Representative example*

**1a** 1-benzyl-5-(1-methyl-1H-imidazole-2-carbonyl)pyrrolidin-2-one. General Procedure I, 25.0 mmol scale, the reaction mixture was purified by column chromatography (0-40% acetone/hexanes) to yield the product as yellow wax, 4.50 g, 64%.

^1^H NMR (500 MHz, CDCl_3_) δ 7.36 – 7.27 (m, 3H), 7.24 – 7.19 (m, 3H), 7.14 (s, 1H), 5.57 – 5.35 (m, 1H), 5.07 (d, *J* = 14.9 Hz, 1H), 4.09 (d, *J* = 14.9 Hz, 1H), 4.01 (s, 3H), 2.70 – 2.57 (m, 1H), 2.57 – 2.45 (m, 2H), 2.24 – 2.07 (m, 1H). ^13^C NMR (126 MHz, CDCl_3_) δ 189.8, 176.0, 141.4, 136.6, 130.1, 128.8, 128.6, 128.0, 127.8, 61.6, 45.9, 36.3, 29.9, 23.9.

FTIR (ATR) cm^-1^: 1676, 1404, 1228, 700.

HRMS (ESI): Mass calcd for C16H18N3O2 [M+H]^+^: 284.1394; found: 284.1400.

**1b** 1-benzyl-5-(1-ethyl-1H-imidazole-2-carbonyl)pyrrolidin-2-one. General Procedure I, 4.0 mmol scale, the reaction mixture was purified by column chromatography (0-40% acetone/hexanes) to yield the product as yellow wax, 890 mg, 75%.

^1^H NMR (500 MHz, CDCl_3_) δ 7.25 – 7.16 (m, 3H), 7.15 – 7.10 (m, 4H), 5.38 – 5.28 (m, 1H), 4.99 (d, *J* = 14.9 Hz, 1H), 4.35 (qd, *J* = 7.1, 2.4 Hz, 2H), 4.00 (d, *J* = 14.9 Hz, 1H), 2.58 – 2.48 (m, 1H), 2.48 – 2.37 (m, 2H), 2.06 – 2.00 (m, 1H), 1.37 (t, *J* = 7.2 Hz, 3H). ^13^C NMR (126 MHz, CDCl_3_) δ 189.2, 175.8, 140.5, 136.3, 130.0, 128.5, 128.4, 127.5, 126.0, 61.5, 45.7, 43.8, 29.7, 23.7, 16.3.

FTIR (ATR) cm^-1^: 1679, 1408, 1255, 1157.

HRMS (ESI): Mass calcd for C17H20N3O2 [M+H]^+^: 298.1550; found: 298.1562.

**1c** 1-benzyl-5-(1-isopropyl-1H-imidazole-2-carbonyl)pyrrolidin-2-one. General Procedure I, 4.0 mmol scale, the reaction mixture was purified by column chromatography (0-40% acetone/hexanes) to yield the product as yellow wax, 1.03 g, 83%.

^1^H NMR (500 MHz, CDCl_3_) δ 7.28 (s, 1H), 7.24 – 7.16 (m, 3H), 7.16 – 7.10 (m, 3H), 5.47 – 5.32 (m, 2H), 4.99 (d, *J* = 14.9 Hz, 1H), 4.00 (d, *J* = 14.9 Hz, 1H), 2.63 – 2.48 (m, 1H), 2.48 – 2.36 (m, 2H), 2.07 – 1.96 (m, 1H), 1.45 – 1.36 (m, 6H). ^13^C NMR (126 MHz, CDCl_3_) δ 189.6, 175.9, 140.5, 136.4, 130.3, 128.6, 128.5, 127.6, 122.1, 61.9, 49.5, 45.8, 29.8, 23.9, 23.7, 23.6.

FTIR (ATR) cm^-1^: 1678, 1453, 1395, 1256.

HRMS (ESI): Mass calcd for C18H22N3O2 [M+H]^+^: 312.1707; found: 312.1715.

**1d** 1-benzyl-5-(1-phenyl-1H-imidazole-2-carbonyl)pyrrolidin-2-one. General Procedure I, 20.0 mmol scale, the reaction mixture was purified by column chromatography (0-40% acetone/hexanes) to yield the product as white solid, 3.90 g, 57%.

^1^H NMR (500 MHz, CDCl_3_) δ 7.49 – 7.41 (m, 3H), 7.28 – 7.19 (m, 5H), 7.19 – 7.12 (m, 4H), 5.39 – 5.31 (m, 1H), 4.94 (d, J = 14.9 Hz, 1H), 3.95 (d, J = 14.9 Hz, 1H), 2.52 – 2.38 (m, 3H), 2.10 – 2.01 (m, 1H). ^13^C NMR (126 MHz, CDCl_3_) δ 188.2, 175.6, 141.0, 137.7, 136.4, 130.4, 129.2, 129.2, 128.7, 128.5, 127.9, 127.6, 125.8, 61.5, 45.7, 29.6, 23.7.

FTIR (ATR) cm^-1^: 1662, 1681, 1490, 1406.

HRMS (ESI): Mass calcd for C21H20N3O2 [M+H]^+^: 346.1550; found: 346.1560.

**1e** 1-(4-chlorobenzyl)-5-(1-isopropyl-1H-imidazole-2-carbonyl)pyrrolidin-2-one. General Procedure I, 1.0 mmol scale, the reaction mixture was purified by column chromatography (0-40% acetone/hexanes) to yield the product as yellow wax, 198 mg, 57%.

^1^H NMR (500 MHz, CDCl_3_) δ 7.29 (s, 1H), 7.23 – 7.13 (m, 3H), 7.09 (d, *J* = 8.4 Hz, 2H), 5.42 – 5.30 (m, 2H), 4.85 (d, *J* = 15.0 Hz, 1H), 4.08 (d, *J* = 15.0 Hz, 1H), 2.61 – 2.49 (m, 1H), 2.49 – 2.36 (m, 2H), 2.07 – 2.00 (m, 1H), 1.44 – 1.37 (m, 6H). ^13^C NMR (126 MHz, CDCl_3_) δ 189.5, 176.0, 140.5, 135.1, 133.5, 130.4, 130.0, 128.8, 122.3, 61.8, 49.6, 45.3, 29.7, 23.9, 23.7, 23.6.

FTIR (ATR) cm^-1^: 1679, 1491, 1404, 1254.

HRMS (ESI): Mass calcd for C18H21ClN3O2 [M+H]^+^: 346.1317; found: 346.1327.

**1f** 5-(1-methyl-1H-imidazole-2-carbonyl)-1-(4-(4,4,5,5-tetramethyl-1,3,2-dioxaborolan-2-yl)benzyl)pyrrolidin-2-one. **1g** (543 mg, 1.5 mmol, 1.0 equiv), B_2_Pin_2_ (457 mg, 1.8 mmol, 1.2 equiv), PdCl_2_(dppf) (61 mg, 0.075 mmol, 5 mol %), KOAc (441 mg, 4.5 mmol, 3.0 equiv) and DMF (8 mL) were added to a 20 mL vial under N_2_. The reaction mixture was stirred at 95 °C for 12 hours. The reaction mixture was cooled to room temperature, then filtered. The residue was diluted with water (50 mL) and extracted with ethyl acetate (3×30 mL). The combined organic layers were washed with brine and dried over Na_2_SO_4_, then concentrated under reduced pressure. The reaction mixture was purified by column chromatography (0-40% acetone/hexanes) to yield the product as yellow solid, 350 mg, 57%, m.p.: 174-176 ^o^C.

^1^H NMR (500 MHz, CDCl_3_) δ 7.68 (d, *J* = 7.5 Hz, 2H), 7.14 (d, *J* = 7.5 Hz, 2H), 7.10 (s, 1H), 7.04 (s, 1H), 5.28 (dd, *J* = 9.0, 3.0 Hz, 1H), 5.03 (d, *J* = 15.0 Hz, 1H), 3.99 (d, *J* = 15.0 Hz, 1H), 3.93 (s, 3H), 2.56 – 2.38 (m, 3H), 2.09 – 2.00 (m, 1H), 1.32 (s, 12H). ^13^C NMR (126 MHz, CDCl_3_) δ 189.4, 175.7, 141.0, 139.4, 135.0, 129.8, 127.7, 127.6, 83.7, 61.3, 45.7, 36.03, 29.6, 24.8, 24.8, 23.6.

FTIR (ATR) cm^-1^: 1612, 1467, 1378, 1160, 1128.

HRMS (ESI): Mass calcd for C22H29BN3O4 [M+H]^+^: 410.2246; found: 410.2251.

**1g** 1-(4-bromobenzyl)-5-(1-methyl-1H-imidazole-2-carbonyl)pyrrolidin-2-one. General Procedure I, 14.0 mmol scale, the reaction mixture was purified by column chromatography (0-40% acetone/hexanes) to yield the product as yellow wax, 3.80 g, 75%.

^1^H NMR (500 MHz, CDCl_3_) δ 7.35 (d, *J* = 8.3 Hz, 2H), 7.13 (s, 1H), 7.07 (s, 1H), 7.02 (d, *J* = 8.3 Hz, 2H), 5.31 (dd, *J* = 9.0, 3.0 Hz, 1H), 4.84 (d, *J* = 15.0 Hz, 1H), 4.05 (d, *J* = 15.0 Hz, 1H), 3.92 (s, 3H), 2.62 – 2.51 (m, 1H), 2.48 – 2.36 (m, 2H), 2.11 – 2.02 (m, 1H). ^13^C NMR (126 MHz, CDCl_3_) δ 189.4, 175.7, 141.1, 135.4, 131.5, 130.1, 129.9, 127.8, 121.5, 61.2, 45., 36.0, 29.5, 23.5.

FTIR (ATR) cm^-1^: 1681, 1404, 1348, 1233.

HRMS (ESI): Mass calcd for C16H17BrN3O2 [M+H]^+^: 362.0499; found: 362.0504.

**1h** 1-(4-bromobenzyl)-5-(1-phenyl-1H-imidazole-2-carbonyl)pyrrolidin-2-one. General Procedure I, 15.0 mmol scale, the reaction mixture was purified by column chromatography (0-40% acetone/hexanes) to yield the product as yellow wax, 3.20 g, 50%, m.p.: 136-139 ^o^C.

^1^H NMR (500 MHz, CDCl_3_) δ 7.56 – 7.46 (m, 3H), 7.45 – 7.38 (m, 2H), 7.32 – 7.24 (m, 2H), 7.21 – 7.15 (m, 2H), 7.12 – 7.03 (m, 2H), 5.43 – 5.38 (m, 1H), 4.80 (d, *J* = 15.0 Hz, 1H), 4.10 (d, *J* = 15.1 Hz, 1H), 2.59 – 2.37 (m, 3H), 2.15 – 2.04 (m, 1H). ^13^C NMR (126 MHz, CDCl_3_) δ 187.9, 175.6, 140.9, 137.5, 135.5, 131.6, 130.3, 130.2, 129.2, 129.1, 128.0, 125.6, 121.5, 61.3, 45.2, 29.4, 23.5.

FTIR (ATR) cm^-1^: 1691, 1678, 1489, 1403.

HRMS (ESI): Mass calcd for C21H19BrN3O2 [M+H]^+^: 424.0655; found: 424.0660.

**1i** 1-([1,1'-biphenyl]-4-ylmethyl)-5-(1-methyl-1H-imidazole-2-carbonyl)pyrrolidin-2-one. General Procedure I, 3.0 mmol scale, the reaction mixture was purified by column chromatography (0-40% acetone/hexanes) to yield the product as yellow wax, 641 mg, 60%, m.p.: 90-94 ^o^C.

^1^H NMR (500 MHz, CDCl_3_) δ 7.54 – 7.51 (m, 2H), 7.46 – 7.40 (m, 4H), 7.35 – 7.31 (m, 1H), 7.23 – 7.19 (m, 2H), 7.12 (s, 1H), 7.04 (s, 1H), 5.38 (dd, *J* = 9.2, 3.0 Hz, 1H), 4.98 (d, *J* = 14.9 Hz, 1H), 4.11 (d, *J* = 14.9 Hz, 1H), 3.90 (s, 3H), 2.61 – 2.51 (m, 1H), 2.50 – 2.38 (m, 2H), 2.12 – 2.04 (m, 1H). ^13^C NMR (126 MHz, CDCl_3_) δ 189.6, 175.8, 141.2, 140.8, 140.5, 135.4, 129.9, 128.9, 128.8, 127.7, 127.4, 127.3, 127.1, 61.4, 45.5, 36.0, 29.7, 23.6.

FTIR (ATR) cm^-1^: 1683, 1405, 1228, 906.

HRMS (ESI): Mass calcd for C22H22N3O2 [M+H]^+^: 360.1707; found: 360.1715.

**1j** 5-(1-methyl-1H-imidazole-2-carbonyl)-1-(4-methylbenzyl)pyrrolidin-2-one. General Procedure I, 3.0 mmol scale, the reaction mixture was purified by column chromatography (0-40% acetone/hexanes) to yield the product as yellow wax, 423 mg, 47%.

^1^H NMR (500 MHz, CDCl_3_) δ 7.10 (s, 1H), 7.05 (s, 1H), 7.02 (s, 4H), 5.35 – 5.24 (m, 1H), 4.95 (d, *J* = 14.8 Hz, 1H), 3.97 – 3.90 (m, 4H), 2.57 – 2.45 (m, 1H), 2.45 – 2.37 (m, 2H), 2.37 – 2.23 (m, 3H), 2.08 – 1.97 (m, 1H). ^13^C NMR (126 MHz, CDCl_3_) δ 189.5, 175.6, 141.13, 137.1, 133.1, 129.7, 129.1, 128.4, 127.6, 61.2, 45.3, 35.9, 29.7, 23.5, 21.0.

FTIR (ATR) cm^-1^: 1681, 1406, 1229, 905.

HRMS (ESI): Mass calcd for C17H20N3O2 [M+H]^+^: 298.1550; found: 298.1560.

**1k** 5-(1-methyl-1H-imidazole-2-carbonyl)-1-(3-methylbenzyl)pyrrolidin-2-one. General Procedure I, 3.0 mmol scale, the reaction mixture was purified by column chromatography (0-40% acetone/hexanes) to yield the product as yellow wax, 840 mg, 94%.

^1^H NMR (500 MHz, CDCl_3_) δ 7.12 – 7.08 (m, 2H), 7.05 (s, 1H), 6.99 (d, *J* = 7.6 Hz, 1H), 6.95 – 6.89 (m, 2H), 5.35 – 5.27 (m, 1H), 4.95 (d, *J* = 14.9 Hz, 1H), 4.00 – 3.89 (m, 4H), 2.57 – 2.47 (m, 1H), 2.47 – 2.37 (m, 2H), 2.24 (s, 3H), 2.13 – 1.98 (m, 1H).^13^C NMR (126 MHz, CDCl_3_) δ 189.5, 175.6, 141.1, 138.2, 136.1, 129.7, 129.0, 128.3, 128.2, 127.6, 125.4, 61.3, 45.6, 36.0, 29.6, 23.5, 21.3.

FTIR (ATR) cm^-1^: 1679, 1405, 1227, 1156.

HRMS (ESI): Mass calcd for C17H20N3O2 [M+H]^+^: 298.1550; found: 298.1557.

**1l** 1-(3-chlorobenzyl)-5-(1-isopropyl-1H-imidazole-2-carbonyl)pyrrolidin-2-one. General Procedure I, 4.0 mmol scale, the reaction mixture was purified by column chromatography (0-40% acetone/hexanes) to yield the product as yellow wax, 990 mg, 72%.

^1^H NMR (500 MHz, CDCl_3_) δ 7.29 (d, *J* = 1.0 Hz, 1H), 7.18 – 7.13 (m, 3H), 7.11 (s, 1H), 7.05 (s, 1H), 5.45 – 5.31 (m, 2H), 4.92 (d, *J* = 15.1 Hz, 1H), 4.00 (d, *J* = 15.1 Hz, 1H), 2.55 – 2.41 (m, 3H), 2.10 – 1.99 (m, 1H), 1.43 – 1.40 (m, 6H). ^13^C NMR (126 MHz, CDCl_3_) δ 189.2, 175.8, 140.3, 138.5, 134.3, 130.3, 129.8, 128.3, 127.7, 126.4, 122.2, 61.7, 49.5, 45.2, 29.5, 23.7, 23.5, 23.5.

FTIR (ATR) cm^-1^: 1683, 1668, 1406, 1394, 1254. HRMS (ESI): Mass calcd for C18H21ClN3O2 [M+H]^+^: 346.1317; found: 346.1325.

**1m** 5-(1-methyl-1H-imidazole-2-carbonyl)-1-(2-methylbenzyl)pyrrolidin-2-one. General Procedure I, 3.0 mmol scale, the reaction mixture was purified by column chromatography (0-40% acetone/hexanes) to yield the product as yellow wax, 518 mg, 58%.

^1^H NMR (500 MHz, CDCl_3_) δ 7.09 – 7.06 (m, 3H), 7.02 (d, *J* = 0.9 Hz, 1H), 7.00 – 6.95 (m, 2H), 5.26 – 5.21 (m, 1H), 4.88 (d, *J* = 15.0 Hz, 1H), 4.18 (d, *J* = 14.9 Hz, 1H), 3.90 (s, 3H), 2.57 – 2.38 (m, 3H), 2.24 (s, 3H), 2.06 – 1.98 (m, 1H). ^13^C NMR (126 MHz, CDCl_3_) δ 189.4, 175.4, 140.9, 137.1, 133.7, 130.4, 129.7, 129.2, 127.7, 127.5, 125.7, 61.2, 43.8, 36.0, 29.6, 23.6, 19.1.

FTIR (ATR) cm^-1^: 1687, 1675, 1399, 1238. HRMS (ESI): Mass calcd for C17H20N3O2 [M+H]^+^: 298.1550; found: 298.1561.

**1n** 5-(1-methyl-1H-imidazole-2-carbonyl)-1-(naphthalen-2-ylmethyl)pyrrolidin-2-one. General Procedure I, 3.0 mmol scale, the reaction mixture was purified by column chromatography (0-40% acetone/hexanes) to yield the product as yellow wax, 608 mg, 61%.

^1^H NMR (500 MHz, CDCl_3_) δ 7.77 – 7.71 (m, 2H), 7.67 – 7.62 (m, 1H), 7.50 (s, 1H), 7.44 – 7.37 (m, 2H), 7.31 (dd, *J* = 8.4, 1.7 Hz, 1H), 7.06 (s, 1H), 6.93 (s, 1H), 5.37 (dd, *J* = 9.3, 3.0 Hz, 1H), 5.00 (d, *J* = 14.8 Hz, 1H), 4.32 (d, *J* = 14.8 Hz, 1H), 3.70 (s, 3H), 2.62 – 2.52 (m, 1H), 2.50 – 2.37 (m, 2H), 2.10 – 2.01 (m, 1H). ^13^C NMR (126 MHz, CDCl_3_) δ 189.5, 175.8, 141.1, 133.8, 133.1, 132.8, 129.7, 128.4, 127.7, 127.7, 127.5, 127.2, 126.5, 126.0, 125.9, 61.3, 46.0, 35.7, 29.7, 23.5.

FTIR (ATR) cm^-1^: 1675, 1407, 1348, 1227.

HRMS (ESI): Mass calcd for C20H20N3O2 [M+H]^+^: 334.1550; found: 334.1552.

**1o** 1-(3-(5-(2-fluorophenyl)-1,2,4-oxadiazol-3-yl)benzyl)-5-(1-methyl-1H-imidazole-2-carbonyl)pyrrolidin-2-one. General Procedure I, 3.0 mmol scale, the reaction mixture was purified by column chromatography (0-40% acetone/hexanes) to yield the product as yellow wax, 520 mg, 79%.

^1^H NMR (400 MHz, CDCl_3_) δ 8.23 – 8.15 (m, 1H), 8.06 – 7.95 (m, 1H), 7.91 (d, *J* = 1.8 Hz, 1H), 7.66 – 7.54 (m, 1H), 7.47 – 7.25 (m, 4H), 7.07 (d, *J* = 0.9 Hz, 1H), 7.01 (d, *J* = 0.9 Hz, 1H), 5.40 – 5.34 (m, 1H), 5.07 (d, *J* = 15.1 Hz, 1H), 4.13 (d, *J* = 15.1 Hz, 1H), 3.93 (s, 3H), 2.60 – 2.41 (m, 3H), 2.15 – 2.03 (m, 1H). ^13^C NMR (151 MHz, CDCl_3_) δ 189.3, 175.8, 172.7 (d, *J* = 4.3 Hz), 168.3, 160.7 (d, *J* = 260.5 Hz), 141.0, 137.2, 134.6 (d, *J* = 8.6 Hz), 131.2, 130.9, 129.8, 129.2, 127.7, 127.3, 126.9, 126.8, 124.7 (d, *J* = 3.8 Hz), 117.1 (d, *J* = 20.9 Hz), 112.7 (d, *J* = 11.4 Hz), 61.3, 45.4, 36.0, 29.5, 23.6. ^19^F NMR (376 MHz, CDCl_3_) δ -108.36.

FTIR (ATR) cm^-1^: 1677, 1618, 1451, 1335.

HRMS (ESI): Mass calcd for C24H21FN5O3 [M+H]^+^: 446.1623; found: 446.1632.

**1p** 1-benzyl-4-(1-methyl-1H-imidazole-2-carbonyl)azetidin-2-one. General Procedure I, 3.0 mmol scale, the reaction mixture was purified by column chromatography (0-40% acetone/hexanes) to yield the product as yellow wax, 650 mg, 60%.

^1^H NMR (500 MHz, CDCl_3_) δ 7.29 – 7.25 (m, 2H), 7.24 – 7.21 (m, 1H), 7.21 – 7.17 (m, 2H), 7.11 (s, 1H), 7.05 (s, 1H), 5.11 (dd, *J* = 5.9, 2.7 Hz, 1H), 4.82 (d, *J* = 15.0 Hz, 1H), 4.28 (d, *J* = 15.0 Hz, 1H), 3.94 (s, 3H), 3.36 (dd, *J* = 14.6, 5.9 Hz, 1H), 3.06 – 2.93 (m, 1H). ^13^C NMR (126 MHz, CDCl_3_) δ 188.0, 166.1, 141.1, 135.4, 129.9, 128.6, 128.3, 127.7, 127.6, 53.3, 45.7, 42.6, 35.9.

FTIR (ATR) cm^-1^: 1741, 1683, 1405, 1389.

HRMS (ESI): Mass calcd for C15H16N3O2 [M+H]^+^: 270.1237; found: 270.1239.

**1q** 1-(4-bromobenzyl)-4-(1-methyl-1H-imidazole-2-carbonyl)azetidin-2-one . General Procedure I, 1.0 mmol scale, the reaction mixture was purified by column chromatography (0-40% acetone/hexanes) to yield the product as yellow wax, 120 mg, 35%.

^1^H NMR (500 MHz, CDCl_3_) δ 7.38 (d, *J* = 8.4 Hz, 2H), 7.11 (s, 1H), 7.09 – 7.05 (m, 3H), 5.11 (dd, *J* = 5.9, 2.7 Hz, 1H), 4.69 (d, *J* = 15.1 Hz, 1H), 4.28 (d, *J* = 15.1 Hz, 1H), 3.93 (s, 3H), 3.34 (dd, *J* = 14.7, 5.9 Hz, 1H), 3.03 (dd, *J* = 14.7, 2.7 Hz, 1H). ^13^C NMR (126 MHz, CDCl_3_) δ 187.7, 166.2, 141.1, 134.4, 131.7, 130.1, 130.0, 127.8, 121.6, 53.4, 45.2, 42.5, 35.9.

FTIR (ATR) cm^-1^: 1750, 1677, 1407, 1488.

HRMS (ESI): Mass calcd for C15H15BrN3O2 [M+H]^+^: 348.0342; found: 348.0350.

**1r** 4-(1-methyl-1H-imidazole-2-carbonyl)-1-(4-methylbenzyl)azetidin-2-one. General Procedure I, 3.5 mmol scale, the reaction mixture was purified by column chromatography (0-40% acetone/hexanes) to yield the product as yellow wax, 511 mg, 52%.

^1^H NMR (500 MHz, CDCl_3_) δ 7.10 (s, 1H), 7.07 (s, 4H), 7.05 (s, 1H), 5.08 (dd, *J* = 5.9, 2.7 Hz, 1H), 4.79 (d, *J* = 14.9 Hz, 1H), 4.23 (d, *J* = 14.9 Hz, 1H), 3.95 (s, 3H), 3.34 (dd, *J* = 14.5, 5.9 Hz, 1H), 2.99 (dd, *J* = 14.5, 2.7 Hz, 1H), 2.29 (s, 3H). ^13^C NMR (126 MHz, CDCl_3_) δ 188.0, 166.1, 141.1, 137.3, 132.2, 129.9, 129.3, 128.3, 127.6, 53.2, 45.4, 42.5, 35.8, 21.0.

FTIR (ATR) cm^-1^: 1742, 1679, 1381, 1409.

HRMS (ESI): Mass calcd for C16H18N3O2 [M+H]^+^: 284.1394; found: 284.1399.

**1a-d_7_** 5-(1-methyl-1H-imidazole-2-carbonyl)-1-((phenyl-d5)methyl-d2)pyrrolidin-2-one . General Procedure I, 5.0 mmol scale, the reaction mixture was purified by column chromatography (0-40% acetone/hexanes) to yield the product as yellow wax, 890 mg, 61%.

^1^H NMR (500 MHz, CDCl_3_) δ 7.09 (s, 1H), 7.04 (s, 1H), 5.32 – 5.27 (m, 1H), 3.91 (s, 3H), 2.57 – 2.47 (m, 1H), 2.46 – 2.36 (m, 2H), 2.07 – 1.99 (m, 1H). ^13^C NMR (126 MHz, CDCl_3_) δ 189.5, 175.7, 141.1, 136.0, 129.8, 128.3 – 127.8 (m), 127.7, 127.3 – 126.8 (m), 61.3, 45.0 (p, *J* = 21.2 Hz), 36.0, 29.7, 23.6.

FTIR (ATR) cm^-1^: 1677, 1410, 1330, 1227.

HRMS (ESI): Mass calcd for C16H11D7N3O2 [M+H]^+^: 291.1833; found: 291.1839.

# Procedure II: procedure for light-induced ring insertion

All reactions were set up under N_2_ atmosphere. To an oven-dried flask containing a stir bar, **1** (0.20 mmol, 1.0 equiv), diphenyl hydrogen phosphate (0.20 mmol, 1.0 equiv) and MeCN (2.0 mL) were added. Parafilm was wrapped around the cap to prevent air from entering, and the vial was stirred and irradiated with 370 nm LEDs. The reactions were monitored by TLC and UPLC-MS. When complete consumption of the **1** was observed (between 1–48 h), the mixture was concentrated under reduced pressure and then purified by column chromatography on silica gel (0-10% (methanol/(dichloromethane/acetone/hexanes (2/1/1))) to give compound **2**

**2a** 5-(1-methyl-1H-imidazole-2-carbonyl)-6-phenylpiperidin-2-one. General Procedure II, 0.2 mmol scale, the reaction mixture was purified by column chromatography (0-10% (dichloromethane/acetone/hexanes(2/1/1))/methanol) to yield the product as yellow wax, 38.5 mg, 68%.

^1^H NMR (500 MHz, CDCl_3_) δ 7.26 – 7.16 (m, 4H), 7.05 (s, 1H), 6.88 – 6.79 (m, 2H), 6.07 (s, 1H), 5.47 (dd, *J* = 6.0, 3.1 Hz, 1H), 4.42 (ddd, *J* = 11.8, 6.0, 3.2 Hz, 1H), 3.74 (s, 3H), 2.76 – 2.67 (m, 1H), 2.57 – 2.46 (m, 1H), 2.24 – 2.12 (m, 1H), 1.93 – 1.84 (m, 1H). ^13^C NMR (126 MHz, CDCl_3_) δ 190.0, 171.6, 142.7, 138.8, 129.4, 128.3, 128.2, 127.1, 126.8, 57.8, 46.4, 35.8, 30.1, 17.9.

FTIR (ATR) cm^-1^: 1670, 1460, 1391.

HRMS (ESI): Mass calcd for C16H18N3O2 [M+H]^+^: 284.1394; found: 284.1398.

**2b** 5-(1-ethyl-1H-imidazole-2-carbonyl)-6-phenylpiperidin-2-one. General Procedure II, 0.2 mmol scale, the reaction mixture was purified by column chromatography (0-10% (dichloromethane/acetone/hexanes(2/1/1))/methanol) to yield the product as yellow wax, 35.8 mg, 60%.

^1^H NMR (500 MHz, CDCl_3_) δ 7.22 – 7.17 (m, 4H), 7.11 (s, 1H), 6.85 (d, *J* = 6.4 Hz, 2H), 6.11 (d, *J* = 5.5 Hz, 1H), 5.51 (dd, *J* = 6.4, 3.1 Hz, 1H), 4.49 – 4.38 (m, 1H), 4.27 (dq, *J* = 14.3, 7.3 Hz, 1H), 4.08 (dq, *J* = 14.2, 7.3 Hz, 1H), 2.78 – 2.67 (m, 1H), 2.56 – 2.46 (m, 1H), 2.26 – 2.14 (m, 1H), 1.95 – 1.85 (m, 1H), 1.22 (t, *J* = 7.3 Hz, 3H). ^13^C NMR (126 MHz, CDCl_3_) δ 189.7, 171.6, 142.1, 138.9, 129.6, 128.3, 128.2, 126.9, 125.5, 57.9, 46.6, 43.7, 30.2, 17.9, 16.1.

FTIR (ATR) cm^-1^: 1673, 1452, 1408, 1357.

HRMS (ESI): Mass calcd for C17H20N3O2 [M+H]^+^: 298.1550; found: 298.1555.

**2c** 5-(1-isopropyl-1H-imidazole-2-carbonyl)-6-phenylpiperidin-2-one. General Procedure II, 0.2 mmol scale, the reaction mixture was purified by column chromatography (0-10% (dichloromethane/acetone/hexanes(2/1/1))/methanol) to yield the product as yellow wax, 42.3 mg, 68%.

^1^H NMR (500 MHz, CDCl_3_) δ 7.24 (s, 1H), 7.22 (s, 1H), 7.20 – 7.15 (m, 3H), 6.86 – 6.80 (m, 2H), 5.96 (s, 1H), 5.49 (dd, *J* = 6.2, 3.2 Hz, 1H), 5.16 – 5.09 (m, 1H), 4.44 (ddd, *J* = 11.9, 6.1, 3.2 Hz, 1H), 2.75 – 2.65 (m, 1H), 2.55 – 2.46 (m, 1H), 2.23 – 2.12 (m, 1H), 1.91 – 1.83 (m, 1H), 1.36 (d, *J* = 6.7 Hz, 3H), 1.22 (d, *J* = 6.7 Hz, 3H). ^13^C NMR (126 MHz, CDCl_3_) δ 190.0, 171.6, 142.0, 138.9, 129.9, 128.2, 128.2, 127.0, 121.4, 58.0, 49.1, 46.9, 30.2, 23.4, 23.3, 17.9.

FTIR (ATR) cm^-1^: 1664, 1465, 1396, 1357.

HRMS (ESI): Mass calcd for C18H22N3O2 [M+H]^+^: 312.1707; found: 312.1710.

**2d** 6-phenyl-5-(1-phenyl-1H-imidazole-2-carbonyl)piperidin-2-one. General Procedure II, 0.2 mmol scale, the reaction mixture was purified by column chromatography (0-10% (dichloromethane/acetone/hexanes(2/1/1))/methanol) to yield the product as yellow wax, 42.3 mg, 65%, 48 h; 1.0 mmol, 189 mg, 55% yield, 3 days.

^1^H NMR (500 MHz, CDCl_3_) δ 7.39 – 7.29 (m, 4H), 7.25 (dd, *J* = 5.0, 1.8 Hz, 3H), 7.18 (s, 1H), 6.95 (dd, *J* = 6.7, 2.8 Hz, 2H), 6.93 – 6.88 (m, 2H), 6.20 (d, *J* = 3.2 Hz, 1H), 5.62 (dd, *J* = 6.2, 3.0 Hz, 1H), 4.41 (ddd, *J* = 11.7, 6.2, 3.3 Hz, 1H), 2.72 – 2.59 (m, 1H), 2.54 – 2.43 (m, 1H), 2.19 – 2.07 (m, 1H), 1.87 – 1.82 (m, 1H). ^13^C NMR (126 MHz, CDCl_3_) δ 188.3, 171.6, 142.4, 139.0, 137.8, 129.9, 128.8, 128.8, 128.4, 128.3, 127.3, 127.2, 125.5, 57.8, 46.5, 30.1, 18.0.

FTIR (ATR) cm^-1^: 1686, 1658, 1493, 1404.

HRMS (ESI): Mass calcd for C21H20N3O2 [M+H]^+^: 346.1550; found: 346.1557.

**2e** 6-(4-chlorophenyl)-5-(1-isopropyl-1H-imidazole-2-carbonyl)piperidin-2-one. General Procedure II, 0.2 mmol scale, the reaction mixture was purified by column chromatography (0-10% (dichloromethane/acetone/hexanes(2/1/1))/methanol) to yield the product as yellow wax, 39.8 mg, 58%.

^1^H NMR (500 MHz, CDCl_3_) δ 7.23 (s, 1H), 7.21 (s, 1H), 7.14 (d, *J* = 8.4 Hz, 2H), 6.78 (d, *J* = 8.5 Hz, 2H), 6.10 (d, *J* = 3.2 Hz, 1H), 5.48 (dd, *J* = 6.1, 3.2 Hz, 1H), 5.17 – 5.11 (m, 1H), 4.41 (ddd, *J* = 11.7, 6.0, 3.1 Hz, 1H), 2.70 – 2.64 (m, 1H), 2.52 – 2.44 (m, 1H), 2.16 – 2.08 (m, 1H), 1.89 – 1.82 (m, 1H), 1.36 (d, *J* = 6.7 Hz, 3H), 1.24 (d, *J* = 6.6 Hz, 3H). ^13^C NMR (126 MHz, CDCl_3_) δ 189.8, 171.6, 141.8, 137.5, 134.1, 130.0, 128.4, 128.3, 121.6, 57.4, 49.2, 46.7, 30.1, 23.4, 23.3, 18.0.

FTIR (ATR) cm^-1^: 1667, 1396, 1466, 1490.

HRMS (ESI): Mass calcd for C18H21ClN3O2 [M+H]^+^: 346.1317; found: 346.1328.

**2f** 5-(1-methyl-1H-imidazole-2-carbonyl)-6-(4-(4,4,5,5-tetramethyl-1,3,2-dioxaborolan-2-yl)phenyl)piperidin-2-one. General Procedure II, 0.2 mmol scale, the reaction mixture was purified by column chromatography (0-10% (dichloromethane/acetone/hexanes(2/1/1))/methanol) to yield the product as yellow wax, 35.8 mg, 44%.

^1^H NMR (400 MHz, CDCl_3_) δ 7.65 (d, *J* = 8.1 Hz, 2H), 7.21 (d, *J* = 1.0 Hz, 1H), 7.05 (d, *J* = 1.0 Hz, 1H), 6.83 (d, *J* = 8.1 Hz, 2H), 5.98 (d, *J* = 3.2 Hz, 1H), 5.48 (dd, *J* = 6.0, 3.2 Hz, 1H), 4.43 (ddd, *J* = 11.8, 5.9, 3.1 Hz, 1H), 3.76 (s, 3H), 2.76 – 2.67 (m, 1H), 2.58 – 2.47 (m, 1H), 2.19 – 2.10 (m, 1H), 1.91 – 1.84 (m, 1H), 1.37 – 1.27 (m, 12H). ^13^C NMR (101 MHz, CDCl_3_) δ 189.8, 171.6, 142.7, 141.8, 134.7, 129.5, 127.1, 126.1, 83.9, 57.9, 46.5, 35.9, 30.1, 24.9, 24.8, 24.7, 17.9.

FTIR (ATR) cm^-1^: 1665, 1612, 1401, 1360.

HRMS (ESI): Mass calcd for C22H29BN3O4 [M+H]^+^: 410.2246; found: 410.2253.

**2g** 6-(4-bromophenyl)-5-(1-methyl-1H-imidazole-2-carbonyl)piperidin-2-one. General Procedure II, 0.2 mmol scale, the reaction mixture was purified by column chromatography (0-10% (dichloromethane/acetone/hexanes(2/1/1))/methanol) to yield the product as yellow wax, 36.7 mg, 51%, m.p.: 174-178 ^o^C.

^1^H NMR (500 MHz, CDCl_3_) δ 7.34 (d, *J* = 8.4 Hz, 2H), 7.20 (d, *J* = 1.0 Hz, 1H), 7.06 (d, *J* = 0.9 Hz, 1H), 6.74 (d, *J* = 8.5 Hz, 2H), 6.18 (s, 1H), 5.45 (dd, *J* = 5.9, 3.1 Hz, 1H), 4.41 (ddd, *J* = 11.6, 6.0, 3.2 Hz, 1H), 3.78 (s, 3H), 2.75 – 2.64 (m, 1H), 2.54 – 2.46 (m, 1H), 2.20 – 2.08 (m, 1H), 1.94 – 1.86 (m, 1H). ^13^C NMR (126 MHz, CDCl_3_) δ 189.8, 171.6, 142.6, 138.0, 131.4, 129.5, 128.5, 127.3, 122.2, 57.4, 46.2, 35.9, 30.0, 18.0.

FTIR (ATR) cm^-1^: 1660, 1485, 1405, 1287.

HRMS (ESI): Mass calcd for C16H17BrN3O2 [M+H]^+^: 362.0499; found: 362.0498.

**2h** 6-(4-bromophenyl)-5-(1-methyl-1H-imidazole-2-carbonyl)piperidin-2-one. General Procedure II, 2.0 mmol scale, the reaction mixture was purified by column chromatography (0-10% (dichloromethane/acetone/hexanes(2/1/1))/methanol) to yield the product as yellow solid, 430 mg, 51%.

^1^H NMR (500 MHz, CDCl_3_) δ 7.43 – 7.29 (m, 6H), 7.19 (s, 1H), 6.98 – 6.89 (m, 2H), 6.84 (d, *J* = 8.1 Hz, 2H), 6.27 – 6.17 (m, 1H), 5.57 (dd, *J* = 6.2, 3.1 Hz, 1H), 4.42 (ddd, *J* = 11.5, 6.2, 3.3 Hz, 1H), 2.70 – 2.60 (m, 1H), 2.53 – 2.41 (m, 1H), 2.18 – 2.03 (m, 1H), 1.90 – 1.83 (m, 1H). ^13^C NMR (126 MHz, CDCl_3_) δ 188.1, 171.5, 142.3, 138.1, 137.7, 131.5, 130.0, 129.0, 128.9, 128.9, 127.5, 125.5, 122.3, 57.3, 46.3, 30.0, 18.2.

FTIR (ATR) cm^-1^: 1679, 1660, 1488, 1401.

HRMS (ESI): Mass calcd for C21H19BrN3O2 [M+H]^+^: 424.0655; found: 424.0655.

**2i** 6-([1,1'-biphenyl]-4-yl)-5-(1-methyl-1H-imidazole-2-carbonyl)piperidin-2-one. General Procedure II, 0.2 mmol scale, the reaction mixture was purified by column chromatography (0-10% (dichloromethane/acetone/hexanes(2/1/1))/methanol) to yield the product as yellow solid, 50.5 mg, 70%.

^1^H NMR (500 MHz, CDCl_3_) δ 7.54 – 7.48 (m, 2H), 7.47 – 7.39 (m, 4H), 7.36 – 7.30 (m, 1H), 7.22 (d, *J* = 1.0 Hz, 1H), 7.05 (d, *J* = 1.0 Hz, 1H), 6.90 (d, *J* = 8.3 Hz, 2H), 6.62 – 6.50 (m, 1H), 5.52 (dd, *J* = 6.0, 3.0 Hz, 1H), 4.45 (ddd, *J* = 11.5, 6.0, 3.2 Hz, 1H), 3.76 (s, 3H), 2.82 – 2.70 (m, 1H), 2.60 – 2.50 (m, 1H), 2.27 – 2.17 (m, 1H), 1.98 – 1.87 (m, 1H). ^13^C NMR (126 MHz, DMSO) δ 190.5, 170.6, 142.7, 139.9, 139.7, 139.7, 129.5, 129.4, 128.7, 127.9, 127.7, 127.0, 126.6, 56.9, 46.9, 35.9, 30.4, 17.6.

FTIR (ATR) cm^-1^: 1679, 1662, 1487, 1405.

HRMS (ESI): Mass calcd for C22H22N3O [M+H]^+^: 360.1707; found: 360.1712.

**2j** 5-(1-methyl-1H-imidazole-2-carbonyl)-6-(p-tolyl)piperidin-2-one. General Procedure II, 0.2 mmol scale, the reaction mixture was purified by column chromatography (0-10% (dichloromethane/acetone/hexanes(2/1/1))/methanol) to yield the product as yellow solid, 48.1 mg, 81%.

^1^H NMR (500 MHz, CDCl_3_) δ 7.21 (s, 1H), 7.05 (s, 1H), 7.02 (d, *J* = 7.9 Hz, 2H), 6.71 (d, *J* = 8.1 Hz, 2H), 5.94 (d, *J* = 3.2 Hz, 1H), 5.44 (dd, *J* = 6.0, 3.2 Hz, 1H), 4.41 (ddd, *J* = 11.9, 5.9, 3.1 Hz, 1H), 3.76 (s, 3H), 2.76 – 2.68 (m, 1H), 2.56 – 2.47 (m, 1H), 2.28 (s, 3H), 2.23 – 2.13 (m, 1H), 1.92 – 1.82 (m, 1H). ^13^C NMR (126 MHz, CDCl_3_) δ 190.1, 171.6, 142.8, 138.0, 135.8, 129.4, 129.0, 127.0, 126.7, 57.6, 46.6, 35.8, 30.2, 21.0, 17.8.

FTIR (ATR) cm^-1^: 1661, 1472, 1408, 1287.

HRMS (ESI): Mass calcd for C17H20N3O2 [M+H]^+^: 298.1550; found: 298.1559.

**2k** 5-(1-methyl-1H-imidazole-2-carbonyl)-6-(m-tolyl)piperidin-2-one. General Procedure II, 0.2 mmol scale, the reaction mixture was purified by column chromatography (0-10% (dichloromethane/acetone/hexanes(2/1/1))/methanol) to yield the product as yellow solid, 39.2 mg, 66%.

^1^H NMR (400 MHz, CDCl_3_) δ 7.21 (d, *J* = 1.0 Hz, 1H), 7.09 – 7.01 (m, 3H), 6.64 (s, 1H), 6.57 (d, *J* = 7.6 Hz, 1H), 6.07 – 5.97 (m, 1H), 5.41 (dd, *J* = 6.0, 3.2 Hz, 1H), 4.41 (ddd, *J* = 11.8, 6.0, 3.2 Hz, 1H), 3.74 (s, 3H), 2.76 – 2.66 (m, 1H), 2.56 – 2.46 (m, 1H), 2.26 – 2.15 (m, 4H), 1.93 – 1.86 (m, 1H). ^13^C NMR (101 MHz, CDCl_3_) δ 190.1, 171.7, 142.8, 138.7, 138.0, 129.4, 129.0, 128.1, 127.5, 127.0, 123.9, 57.8, 46.4, 35.7, 30.1, 21.4, 17.8.

FTIR (ATR) cm^-1^: 1694, 1491, 1338, 1436.

HRMS (ESI): Mass calcd for C17H20N3O2 [M+H]^+^: 298.1550; found: 298.1555.

**2l** 6-(3-chlorophenyl)-5-(1-isopropyl-1H-imidazole-2-carbonyl)piperidin-2-one. General Procedure II, 0.2 mmol scale, the reaction mixture was purified by column chromatography (0-10% (dichloromethane/acetone/hexanes(2/1/1))/methanol) to yield the product as yellow solid, 38.3 mg, 55%.

^1^H NMR (400 MHz, CDCl_3_) δ 7.31 (d, *J* = 1.1 Hz, 1H), 7.25 – 7.23 (m, 1H), 7.19 – 7.13 (m, 2H), 6.83 (dt, *J* = 7.1, 1.9 Hz, 1H), 6.70 (t, *J* = 1.9 Hz, 1H), 6.39 – 6.29 (m, 1H), 5.50 (dd, *J* = 6.1, 3.3 Hz, 1H), 5.22 – 5.13 (m, 1H), 4.49 – 4.39 (m, 1H), 2.71 (ddd, *J* = 18.1, 5.8, 2.9 Hz, 1H), 2.50 (ddd, *J* = 18.2, 11.7, 6.8 Hz, 1H), 2.22 – 2.08 (m, 1H), 1.91 – 1.86 (m, 1H), 1.39 (d, *J* = 6.7 Hz, 3H), 1.30 (d, *J* = 6.7 Hz, 3H). ^13^C NMR (101 MHz, CDCl_3_) δ 189.6, 171.6, 141.8, 141.1, 134.2, 130.1, 129.6, 128.3, 127.1, 125.2, 121.7, 57.5, 49.3, 46.8, 30.2, 23.4, 23.4, 17.8.

FTIR (ATR) cm^-1^: 1662, 1466, 1395, 1254.

HRMS (ESI): Mass calcd for C18H21ClN3O2 [M+H]^+^: 346.1317; found: 346.1321.

**2m** 5-(1-methyl-1H-imidazole-2-carbonyl)-6-(o-tolyl)piperidin-2-one. General Procedure II, 0.2 mmol scale, the reaction mixture was purified by column chromatography (0-10% (dichloromethane/acetone/hexanes(2/1/1))/methanol) to yield the product as yellow solid, 31.1 mg, 52%.

^1^H NMR (400 MHz, CDCl_3_) δ 7.30 (dd, *J* = 7.8, 1.5 Hz, 1H), 7.16 – 7.11 (m, 1H), 7.09 – 7.05 (m, 1H), 7.01 – 6.97 (m, 2H), 6.89 (d, *J* = 0.9 Hz, 1H), 5.86 (s, 1H), 5.32 (dd, *J* = 5.8, 1.8 Hz, 1H), 4.78 (ddd, *J* = 7.4, 5.7, 4.1 Hz, 1H), 3.71 (s, 3H), 2.87 – 2.76 (m, 1H), 2.58 – 2.49 (m, 1H), 2.39 – 2.31 (m, 1H), 2.11 – 2.04 (m, 4H). ^13^C NMR (101 MHz, CDCl_3_) δ 190.9, 172.1, 143.0, 136.5, 135.2, 130.5, 129.0, 127.7, 127.3, 126.9, 125.9, 54.4, 42.3, 35.8, 29.1, 21.3, 18.8.

FTIR (ATR) cm^-1^: 1658, 1462, 1410, 1360.

HRMS (ESI): Mass calcd for C17H20N3O2 [M+H]^+^: 298.1550; found: 298.1556.

**2n** 5-(1-methyl-1H-imidazole-2-carbonyl)-6-(naphthalen-2-yl)piperidin-2-one. General Procedure II, 0.2 mmol scale, the reaction mixture was purified by column chromatography (0-10% (dichloromethane/acetone/hexanes(2/1/1))/methanol) to yield the product as yellow solid, 26.6 mg, 40%.

^1^H NMR (500 MHz, CDCl_3_) δ 7.79 – 7.73 (m, 1H), 7.67 (d, *J* = 8.4 Hz, 2H), 7.48 – 7.43 (m, 2H), 7.32 – 7.29 (m, 1H), 7.23 (s, 1H), 7.05 (s, 1H), 6.91 (dd, *J* = 8.5, 1.8 Hz, 1H), 6.17 (d, *J* = 3.0 Hz, 1H), 5.63 (dd, *J* = 6.1, 3.1 Hz, 1H), 4.51 (ddd, *J* = 11.6, 6.0, 3.2 Hz, 1H), 3.64 (s, 3H), 2.78 (ddd, *J* = 18.1, 5.8, 3.3 Hz, 1H), 2.56 (ddd, *J* = 18.1, 11.4, 6.7 Hz, 1H), 2.29 – 2.21 (m, 1H), 1.97 – 1.88 (m, 1H). ^13^C NMR (126 MHz, CDCl_3_) δ 190.0, 171.7, 142.8, 136.3, 133.0, 132.9, 129.5, 128.0, 127.8, 127.6, 127.1, 126.5, 126.3, 126.0, 124.5, 58.0, 46.5, 35.8, 30.2, 18.1.

FTIR (ATR) cm^-1^: 1657, 1405, 1365, 1465.

HRMS (ESI): Mass calcd for C20H20N3O2 [M+H]^+^: 334.1550; found: 334.1551.

**2o** 6-(3-(5-(2-fluorophenyl)-1,2,4-oxadiazol-3-yl)phenyl)-5-(1-methyl-1H-imidazole-2-carbonyl)piperidin-2-one. General Procedure II, 0.2 mmol scale, the reaction mixture was purified by column chromatography (0-10% (dichloromethane/acetone/hexanes(2/1/1))/methanol) to yield the product as yellow solid, 15.0 mg, 34%, and recover **1o** 19.9 mg.

^1^H NMR (500 MHz, CDCl_3_) δ 8.18 (td, *J* = 7.4, 1.8 Hz, 1H), 8.05 (d, *J* = 7.7 Hz, 1H), 7.65 – 7.59 (m, 1H), 7.47 – 7.39 (m, 2H), 7.37-7.34 (m, 1H), 7.33 – 7.28 (m, 1H), 7.26 – 7.21 (m, 2H), 7.11 (s, 1H), 6.12 (d, *J* = 3.4 Hz, 1H), 5.58 (dd, *J* = 5.9, 3.3 Hz, 1H), 4.51 (ddd, *J* = 11.8, 5.9, 3.1 Hz, 1H), 3.75 (s, 3H), 2.83 – 2.70 (m, 1H), 2.56 (s, 1H), 2.23 – 2.11 (m, 1H), 1.96 – 1.88 (m, 1H). ^13^C NMR (126 MHz, CDCl_3_) δ 189.8, 172.7 (d, *J* = 4.5 Hz), 171.6, 168.2, 160.7 (d, *J* = 260.6 Hz), 142.6, 139.8, 134.7 (d, *J* = 8.6 Hz), 130.9, 129.8, 129.6, 129.1, 127.6, 127.4, 126.7, 125.8, 124.7 (d, *J* = 3.8 Hz), 117.2 (d, *J* = 20.9 Hz), 112.7 (d, *J* = 11.3 Hz), 57.7, 46.5, 35.9, 30.2, 17.7. ^19^F NMR (376 MHz, CD_3_CN) δ -110.41.

FTIR (ATR) cm^-1^: 1660, 1621, 1462, 1405, 1357.

HRMS (ESI): Mass calcd for C24H21FN5O3 [M+H]^+^: 446.1623; found: 446.1636.

**2p** 4-(1-methyl-1H-imidazole-2-carbonyl)-5-phenylpyrrolidin-2-one. General Procedure II, 0.1 mmol scale, the reaction mixture was purified by column chromatography (0-10% (methanol/dichloromethane) to yield the product as yellow solid, 19.1 mg, 71%, m.p.: 201-205 ^o^C.

^1^H NMR (500 MHz, CDCl_3_) δ 7.19 – 7.08 (m, 4H), 6.90 (s, 1H), 6.88 – 6.81 (m, 2H), 5.91 (s, 1H), 5.30 (d, *J* = 9.4 Hz, 1H), 5.08 (q, *J* = 9.4 Hz, 1H), 3.40 (s, 3H), 3.25 (dd, *J* = 17.5, 9.5 Hz, 1H), 2.41 (dd, *J* = 17.5, 9.3 Hz, 1H). ^13^C NMR (126 MHz, CDCl_3_) δ 188.0, 176.9, 143.0, 138.2, 129.3, 128.1, 128.0, 126.8, 126.6, 59.8, 47.4, 35.2, 29.1.

FTIR (ATR) cm^-1^: 1681, 1412, 1233, 1157.

HRMS (ESI): Mass calcd for C15H16N3O2 [M+H]^+^: 270.1237; found: 270.1239.

**2q** 5-(4-bromophenyl)-4-(1-methyl-1H-imidazole-2-carbonyl)pyrrolidin-2-one. General Procedure II, 0.1 mmol scale, the reaction mixture was purified by column chromatography (0-10% (dichloromethane/methanol) to yield the product as yellow solid, 17.7 mg, 51%.

^1^H NMR (500 MHz, CDCl_3_) δ 7.24 (d, *J* = 8.4 Hz, 2H), 7.16 (s, 1H), 6.94 (s, 1H), 6.74 (d, *J* = 8.4 Hz, 2H), 5.99 (s, 1H), 5.29 (d, *J* = 9.4 Hz, 1H), 5.11 – 5.03 (m, 1H), 3.50 (s, 3H), 3.22 (dd, *J* = 17.5, 9.6 Hz, 1H), 2.41 (dd, *J* = 17.5, 9.3 Hz, 1H). ^13^C NMR (126 MHz, CDCl_3_) δ 187.7, 176.8, 142.8, 137.4, 131.1, 129.4, 128.4, 127.1, 122.1, 59.2, 47.3, 35.3, 29.1.

FTIR (ATR) cm^-1^: 1680, 1488, 1413, 1233.

HRMS (ESI): Mass calcd for C15H15BrN3O2 [M+H]^+^: 348.0342; found: 348.0347.

**2r** 4-(1-methyl-1H-imidazole-2-carbonyl)-5-(p-tolyl)pyrrolidin-2-one. General Procedure II, 0.1 mmol scale, the reaction mixture was purified by column chromatography (0-10% (dichloromethane/methanol) to yield the product as yellow solid, 19.0 mg, 67%.

^1^H NMR (500 MHz, CDCl_3_) δ 7.15 (s, 1H), 6.98 – 6.88 (m, 3H), 6.70 (d, *J* = 8.1 Hz, 2H), 6.01 (s, 1H), 5.26 (d, *J* = 9.3 Hz, 1H), 5.04 (q, *J* = 9.4 Hz, 1H), 3.42 (s, 3H), 3.23 (dd, *J* = 17.4, 9.7 Hz, 1H), 2.37 (dd, *J* = 17.5, 9.2 Hz, 1H), 2.23 (s, 3H). ^13^C NMR (126 MHz, CDCl_3_) δ 188.2, 177.1, 143.1, 138.0, 135.3, 129.4, 128.8, 126.9, 126.6, 59.7, 47.7, 35.3, 29.2, 21.1.

FTIR (ATR) cm^-1^: 1684, 1418, 1379, 1235.

HRMS (ESI): Mass calcd for C16H18N3O2 [M+H]^+^: 284.1394; found: 284.1400.

Failde exemple

We hypothesized that the six- to seven-membered ring transformation failed because the seven-membered ring formation is thermodynamically unfavorable. Crude ¹H-NMR analysis revealed trace amounts of an imine intermediate. For the proline derivative, multiple sites were available for the HAT process, leading to a lack of selectivity, and only trace amounts of the product were observed by crude ¹H-NMR. When heteroaromatic substrates were tested under the standard conditions, no desired products were obtained, and unidentified byproducts were observed. For the indoline substrate, only a redox product was obtained, with no detectable amounts of the desired product. For the *N*-substituted examples containing alkyl, alkene, or propargyl groups, the only observed products were those resulting from the Norrish-Yang cycloaddition. Changing the chromophore activation group to thiazole, picolinoyl, or quinoline did not work either and resulted in unidentified products.

# Procedure for asymmetric synthesis of piperidones

To an oven-dried flask containing a stir bar, **1d** (0.20 mmol, 1.0 equiv), *(R)-*TRIP (0.020 mmol, 10 mol %) or *(S)-*TRIP (0.020 mmol, 10 mol %) and 1,4-dioxane (2.0 mL) were added. Parafilm was wrapped around the cap to prevent air from entering, and the vial was stirred and irradiated with 370 nm LEDs. The reactions were monitored by TLC and UPLC-MS. When complete consumption of the **1d** was observed (48 h), the mixture was concentrated under reduced pressure and then purified by column chromatography on silica gel (0-10% (dichloromethane/acetone/hexanes(2/1/1))/methanol) to give compound ***cis*-2d**.

To an oven-dried flask containing a stir bar, ***cis*-2d** (0.10 mmol, 1.0 equiv) and *i*-PrOH (1.0 mL) were added. Parafilm was wrapped around the cap to prevent air from entering, and the vial was stirred and heated to 100 ^o^C. The reactions were monitored by TLC and UPLC-MS. When complete consumption of the ***cis*-2d** was observed (48 h), the mixture was concentrated under reduced pressure and then purified by column chromatography on silica gel (0-10% (dichloromethane/acetone/hexanes(2/1/1))/methanol) to give compound ***trans*-2d**.

(5*S*,6*R*)-**2d**: (*R*)-TRIP was used, as pale-yellow wax, 47.0 mg, 68%, dr >20:1, *ee* 90%. HPLC (Chiralpak IC, *i-*PrOH/*n*-Hexane = 30/70, flow rate 1.0 mL/min, 30 ^o^C, λ = 280 nm): t_R_ (major) = 32.8 min, t_R_ (minor) = 46.7 min.

(5*R*,6*S*)-**2d**: (*S*)-TRIP was used, as pale-yellow wax, 42.9 mg, 62%, dr >20:1, *ee* 90%. HPLC (Chiralpak IC, *i-*PrOH/*n*-Hexane = 30/70, flow rate 1.0 mL/min, 30 ^o^C, λ = 280 nm): t_R_ (minor) = 32.8 min, t_R_ (major) = 46.7 min.

(5*S*,6*R*)-**2s**: 0.10 mmol scale, (*R*)-TRIP was used, as pale-yellow wax, 17.2 mg, 48%, dr >20:1, *ee* 74%. HPLC (Chiralpak IC, *i-*PrOH/*n*-Hexane = 30/70, flow rate 1.0 mL/min, 30 ^o^C, λ = 280 nm): t_R_ (major) = 35.6 min, t_R_ (minor) = 52.5 min.

(5*R*,6*R*)-**2d**: from (5*S*,6*R*)-**2d**, 0.1 mmol scale, as white wax, 27.6 mg, 80%, dr = 10:1, *ee* 90%. HPLC (Chiralpak IC, *i-*PrOH/*n*-Hexane = 30/70, flow rate 1.0 mL/min, 30 ^o^C, λ = 280 nm): t_R_ (major) = 27.9 min, t_R_ (minor) = 35.2 min.

(5*S*,6*S*)-**2d**: from (5*R*,6*S*)-**2d**, 0.1 mmol scale, as white wax, 25.9 mg, 75%, dr = 10:1, *ee* 89%. HPLC Chiralpak IC, *i-*PrOH/*n*-Hexane = 30/70, flow rate 1.0 mL/min, 30 ^o^C, λ = 280 nm): t_R_ (minor) = 27.9 min, t_R_ (major) = 35.2 min.

Failed examples

# Transformation

**Fig. S1.** Transformation of **2d** to **3**

In a flame dried Schlenk flask, MeMgCl (3.0 M in Et_2_O, 0.2 mL, 0.6 mmol, 3.0 equiv) was added portionwise to a solution of the **2d** (69.1 mg, 0.2 mmol, 1.0 equiv) in dry THF (2.0 mL) at room temperature. The reaction was stirred for 4 hours and monitored by TLC and UPLC-MS. Then aqueous saturated NH_4_Cl solution (5 mL) was added to quench the reaction. The solution was extracted with ethyl acetate, washed with brine, dried over Na_2_SO_4_, filtered and concentrated on rotavapor under reduced pressure. The reaction crude was redissolved in *i*-PrOH (2.0 mL) and heated to 90 ^o^C for 16 hours and monitored by TLC and UPLC-MS. (*Note: The relative configuration of* ***3*** *was determined by ¹H-NMR, along with the epimerization process from cis-****2d*** *to trans-****2d****. We monitored this transformation using crude ¹H-NMR, which initially showed two diastereomers. After heating for 16 hours, only a single diastereomer remained*.) Then the reaction mixture was concentrated under reduced pressure and the residue was purified by column chromatography on silica gel (0-10% DCM/MeOH) to give 5-acetyl-6-phenylpiperidin-2-one **3** (30.1 mg, 69%, dr 10:1) as colorless oil.

^1^H NMR (500 MHz, CDCl_3_) δ 7.38 – 7.28 (m, 3H), 7.20 – 7.16 (m, 2H), 6.16 (s, 1H), 4.99 (dd, *J* = 5.5, 2.9 Hz, 1H), 3.15 (ddd, *J* = 10.6, 5.4, 3.4 Hz, 1H), 2.71 (ddd, *J* = 18.2, 6.2, 4.3 Hz, 1H), 2.46 (ddd, *J* = 18.2, 9.8, 7.1 Hz, 1H), 2.17 – 2.07 (m, 1H), 1.94 (ddd, *J* = 14.4, 7.3, 3.7 Hz, 1H), 1.82 (s, 3H). ^13^C NMR (126 MHz, CDCl_3_) δ 207.5, 171.5, 138.2, 128.7, 128.5, 127.1, 57.6, 51.0, 29.8, 29.7, 19.1.

FTIR (ATR) cm^-1^: 1709, 1653, 1406, 1353.

HRMS (ESI): Mass calcd for C13H16NO2 [M+H]^+^: 218.1176; found: 218.1176.

**Fig. S2.** Transformation of **2d** to **4**

In a flame dried Schlenk flask, TFAA (56 uL, 0.4 mmol, 4.0 equiv) was added dropwise to a solution of the **2d** (34.5 mg, 0.1 mmol, 1.0 equiv), carbamide peroxide (113 mg, 1.2 mmol, 12.0 equiv), and disodium phosphate (142 mg, 1.0 mmol, 10.0 equiv) in dry DCM (2.0 mL) at room temperature. The reaction was stirred for 16 hours at 40 ^o^C and monitored by TLC and UPLC-MS. Then aqueous saturated Na_2_SO_3_ solution (5 mL) was added to quench the reaction. The solution was extracted with ethyl acetate, washed with brine, dried over Na_2_SO_4_, filtered and concentrated on rotavapor under reduced pressure. Then the reaction mixture was concentrated under reduced pressure and the residue was purified by column chromatography on silica gel (0-10% DCM/MeOH) to 5-hydroxy-6-phenyl-5-(1-phenyl-1*H*-imidazole-2-carbonyl)piperidin-2-one **4** (27.1 mg, 75%) as colorless oil.

^1^H NMR (500 MHz, CDCl_3_) δ 7.46 – 7.40 (m, 1H), 7.40 – 7.34 (m, 3H), 7.31 – 7.27 (m, 3H), 7.21 (s, 1H), 7.01 – 6.96 (m, 2H), 6.94 – 6.86 (m, 3H), 5.94 (d, *J* = 3.1 Hz, 1H), 5.33 (d, *J* = 3.1 Hz, 1H), 2.90 – 2.78 (m, 1H), 2.65 – 2.58 (m, 1H), 2.42 – 2.32 (m, 1H), 1.97 – 1.90 (m, 1H). ^13^C NMR (126 MHz, CDCl_3_) δ 186.7, 171.6, 141.2, 138.0, 137.2, 129.1, 128.9, 128.9, 128.6, 128.5, 127.4, 126.8, 125.5, 78.8, 66.5, 27.0, 24.3.

FTIR (ATR) cm^-1^: 1688, 1662, 1493, 1393.

HRMS (ESI): Mass calcd for C21H20N3O3 [M+H]^+^: 362.1499; found: 362.1512.

**Fig. S3.** Transformation of (5*S*,6*R*)-**2d** to **5**

In a flame dried Schlenk flask, NaBH_4_ (3.8 mg, 0.10 mmol, 1.0 equiv) was added to a solution of the (5*S*,6*R*)-**2d** (34.5 mg, 0.1 mmol, 1.0 equiv) in THF/MeOH (1.0 mL, 10/1) at room temperature. The reaction was stirred for 4 hours at 25 ^o^C and monitored by TLC and UPLC-MS. Then aqueous saturated Na_2_SO_3_ solution (5 mL) was added to quench the reaction. The solution was extracted with ethyl acetate, washed with brine, dried over Na_2_SO_4_, filtered and concentrated on rotavapor under reduced pressure. Then the reaction mixture was concentrated under reduced pressure and the residue was purified by column chromatography on silica gel (0-10% DCM/MeOH) to 5-(hydroxy(1-phenyl-1*H*-imidazol-2-yl)methyl)-6-phenylpiperidin-2-one **5** (28.2 mg, 81%) as white solid, m.p.: 190-192 ^o^C. The absolute configuration of **5** was determined to be (*5S, 6R*) by X-ray crystallography when (*5S,6R*)-**2d** was utilized, and other products were assigned by analogy.

^1^H NMR (500 MHz, CDCl_3_) δ 7.34 – 7.21 (m, 8H), 7.20 – 7.13 (m, 2H), 7.05 – 7.01 (m, 1H), 6.99 – 6.95 (m, 1H), 6.66 (d, *J* = 4.1 Hz, 1H), 5.14 (t, *J* = 4.6 Hz, 1H), 4.82 (s, 1H), 3.98 (d, *J* = 10.7 Hz, 1H), 2.90 – 2.78 (m, 1H), 2.50 – 2.31 (m, 2H), 1.27 – 1.13 (m, 1H), 1.03 – 0.93 (m, 1H). ^13^C NMR (126 MHz, CDCl_3_) δ 172.2, 148.4, 138.2, 136.5, 129.3, 128.5, 128.1, 128.0, 127.8, 127.7, 125.5, 121.0, 65.5, 56.6, 44.0, 30.7, 17.8.

FTIR (ATR) cm^-1^: 1648, 1599, 1499, 1306.

HRMS (ESI): Mass calcd for C21H22N3O2 [M+H]^+^: 348.1707; found: 348.1711.

# TEMPO-trapping experiment

**Fig. S4.** Radical trapping experiment

To an oven-dried 8.0-mL vial containing a stir bar, **1d** (34.5 mg, 0.1 mmol, 1.0 equiv), diphenyl hydrogen phosphate (25.0 mg, 0.10 mmol, 1.0 equiv), TEMPO (31.2 mg, 0.2 mmol, 2.0 equiv) and MeCN (1.0 mL) were added. Parafilm was wrapped around the cap to prevent air from entering, and the vial was stirred and irradiated with 370 nm LEDs for 1.0 h. The solvent was removed under reduced pressure, 1,3,5-trimethoxybenzene was added as an internal standard, and CDCl_3_ was added to dissolve all reaction components. The solution was then filtered through a cotton and celite plug into an NMR tube for ^1^H NMR analysis shown without detected amount of **2d**.

# O_2_-quench experiment

**Fig. S5.** O2-quench experiment

To an oven-dried 8.0-mL vial containing a stir bar, **1d** (34.5 mg, 0.1 mmol, 1.0 equiv), diphenyl hydrogen phosphate (25.0 mg, 0.10 mmol, 1.0 equiv), and MeCN (1.0 mL) were added. The vial was stirred under Air balloon and irradiated with 370 nm LEDs for 1.0 h. The solvent was removed under reduced pressure, 1,3,5-trimethoxybenzene was added as an internal standard, and CDCl_3_ was added to dissolve all reaction components. The solution was then filtered through a cotton and celite plug into an NMR tube for ^1^H NMR analysis shown **2d** with 6% yield.

# H_2_O-quench experiment

**Fig. S6.** H_2_O-quench experiment

To an oven-dried 8.0-mL vial containing a stir bar, **1d** (34.5 mg, 0.1 mmol, 1.0 equiv), diphenyl hydrogen phosphate (25.0 mg, 0.10 mmol, 1.0 equiv), water (18.0 mg, 1.0 mmol, 10.0 equiv) and MeCN (1.0 mL) were added. The vial was stirred under N_2_ and irradiated with 370 nm LEDs for 1.0 h. The solvent was removed under reduced pressure, 1,3,5-trimethoxybenzene was added as an internal standard, and CDCl_3_ was added to dissolve all reaction components. The solution was then filtered through a cotton and celite plug into an NMR tube for ^1^H NMR analysis shown **2d** with 10% yield.

# UV/Vis data

UV/Vis data was collected at room temperature under an atmosphere of nitrogen. Solutions were prepared in MeCN purchased from Sigma-Aldrich and measured in a quartz cuvette. **1a** (0.1 mM), and diphenyl hydrogen phosphate (x equiv.).


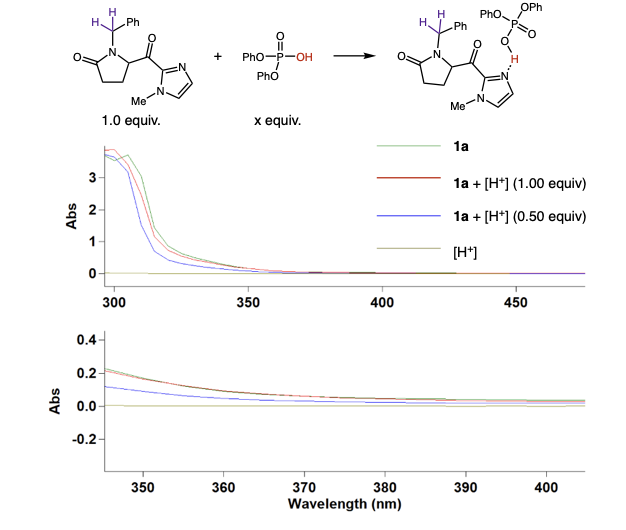


**Fig. S7.** UV/Vis Data

# Parallel KIE

A Young style NMR tube with a rubber valve was added **1a** or **1a-d_6_** (0.05 mmol, 1.0 equiv), PO(OPh)_2_OH (12.5 mg, 0.05 mmol, 1.0 equiv) and MeCN-d_3_ (0.5 mL) were added. The NMR tube irradiated with 370 nm LEDs and ^1^H-NMR spectrum were then taken about every 3 min in initial 18 min. The initial parallel KIE is 1.17 indicated that the 1,5-HAT process wasn’t likely the rate determining step.

| \| time (min) \| **2a**(yield) \| **2a-d_7_**(yield) \| \| --- \| --- \| --- \| \| 0 \| 0.0 \| 0.0 \| \| 3 \| 3.5 \| 1.1 \| \| 6 \| 10.0 \| 6.8 \| \| 9 \| 16.0 \| 11.9 \| \| 12 \| 20.3 \| 18.6 \| \| 15 \| 25.4 \| 20.7 \| |
| --- | --- | --- | --- | --- | --- | --- | --- | --- | --- | --- | --- | --- | --- | --- | --- | --- | --- | --- | --- | --- | --- |


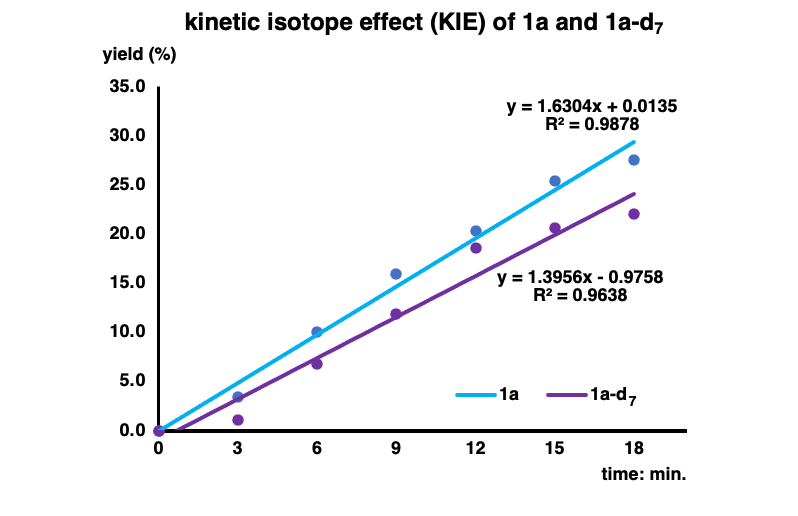


# Light off and on experiment

**Fig. S8.** UV/Vis Data

A Young style NMR tube with a rubber valve was added **1a** (0.05 mmol, 1.0 equiv), PO(OPh)_2_OH (12.5 mg, 0.05 mmol, 1.0 equiv) and MeCN-d_3_ (0.5 mL) were added. The NMR tube irradiated with 370 nm LEDs and dark in turn.

# Nonlinear effects

To an oven-dried flask containing a stir bar, **1d** (0.10 mmol, 1.0 equiv), *(R)-*TRIP (xx% ee,10 mol %) and 1,4-dioxane (1.0 mL) were added. Parafilm was wrapped around the cap to prevent air from entering, and the vial was stirred and irradiated with 370 nm LEDs. The reactions were monitored by TLC and UPLC-MS. When complete consumption of the **1d** was observed (48 h), the mixture was concentrated under reduced pressure and then purified by column chromatography on silica gel (0-10% (dichloromethane/acetone/hexanes(2/1/1))/methanol) to give compound ***cis*-2d**.

| Entry | *(R)-*TRIP (xx% ee) | ***cis*-2d** (ee %) |
| --- | --- | --- |
| 1 | 0% ee | 2% ee |
| 2 | 20% ee | 35% ee |
| 3 | 40% ee | 53% ee |
| 4 | 60% ee | 72% ee |
| 5 | 80% ee | 83% ee |
| 6 | 100% ee | 90% ee |

**Fig. S9.** Nonlinear effects

# Crystal structures

Crystallographic files (CDCC: 2392293 (**2i**)).


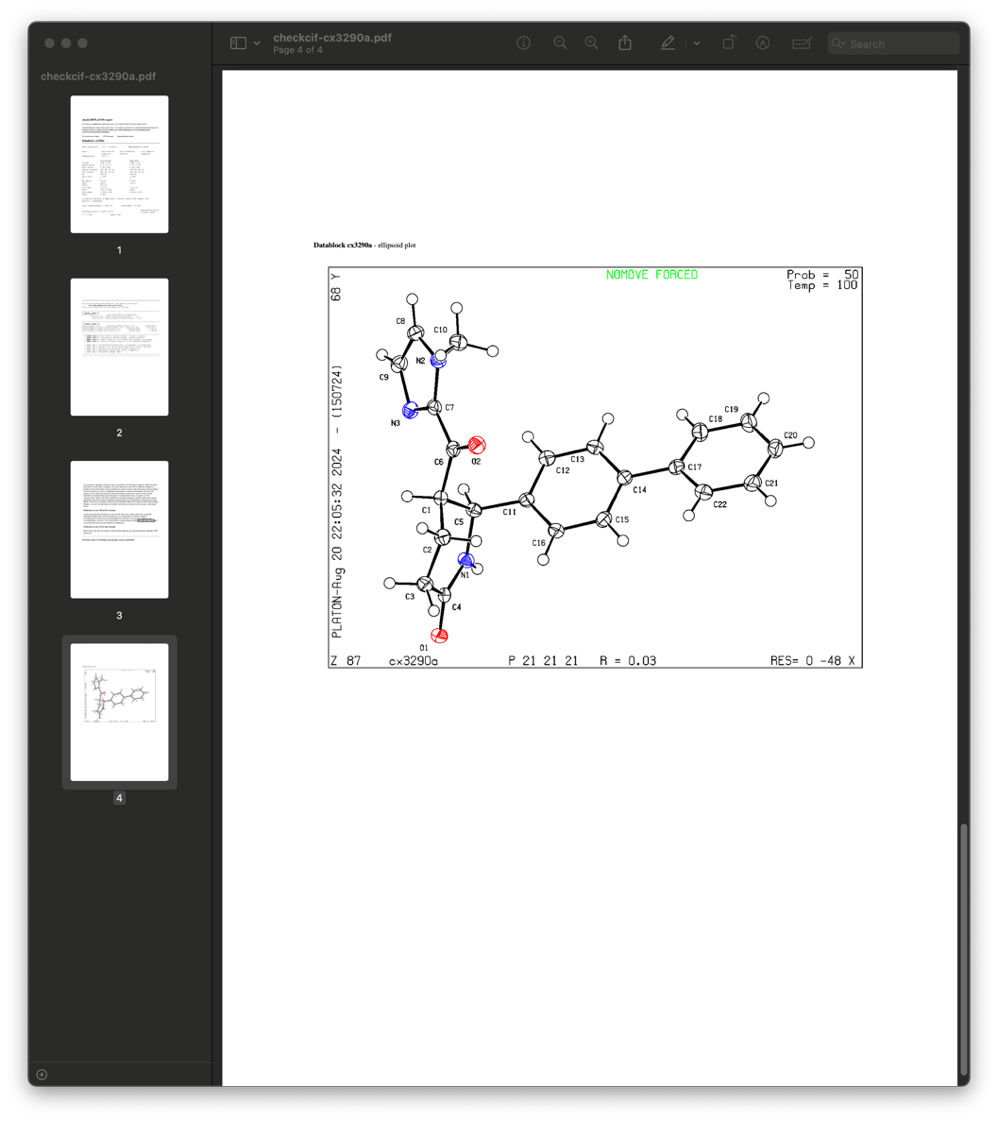


| **Table S1 Crystal data and structure refinement for cx3290a.** | |
| --- | --- |
| Identification code | cx3290a |
| Empirical formula | C_22_H_21_N_3_O_2_ |
| Formula weight | 359.42 |
| Temperature/K | 99.99(10) |
| Crystal system | orthorhombic |
| Space group | P2_1_2_1_2_1_ |
| a/Å | 6.07541(6) |
| b/Å | 16.79068(19) |
| c/Å | 17.4856(2) |
| α/° | 90 |
| β/° | 90 |
| γ/° | 90 |
| Volume/Å^3^ | 1783.71(3) |
| Z | 4 |
| ρ_calc_g/cm^3^ | 1.338 |
| μ/mm^‑1^ | 0.699 |
| F(000) | 760.0 |
| Crystal size/mm^3^ | 0.07 × 0.027 × 0.023 |
| Radiation | Cu Kα (λ = 1.54184) |
| 2Θ range for data collection/° | 7.3 to 151.1 |
| Index ranges | -7 ≤ h ≤ 5, -21 ≤ k ≤ 21, -21 ≤ l ≤ 20 |
| Reflections collected | 17801 |
| Independent reflections | 3608 [R_int_ = 0.0284, R_sigma_ = 0.0216] |
| Data/restraints/parameters | 3608/0/260 |
| Goodness-of-fit on F^2^ | 1.048 |
| Final R indexes [I>=2σ (I)] | R_1_ = 0.0276, wR_2_ = 0.0685 |
| Final R indexes [all data] | R_1_ = 0.0291, wR_2_ = 0.0693 |
| Largest diff. peak/hole / e Å^-3^ | 0.17/-0.15 |

Crystallographic files (CDCC: 2392294 (**2p**)).


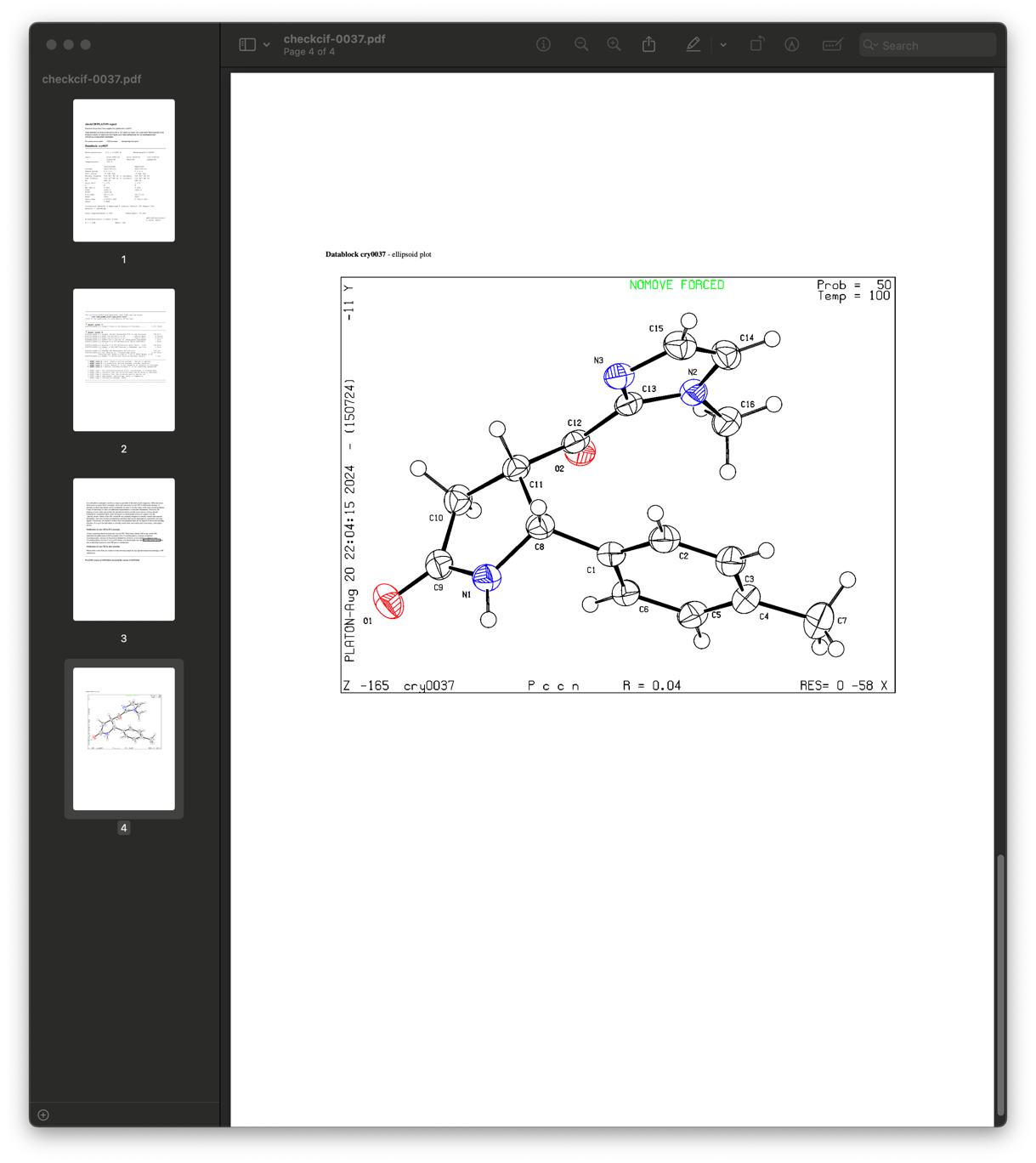


| **Table S2 Crystal data and structure refinement for cry0037.** | |
| --- | --- |
| Identification code | cry0037 |
| Empirical formula | C_16_H_17_N_3_O_2_ |
| Formula weight | 283.32 |
| Temperature/K | 100.01(10) |
| Crystal system | orthorhombic |
| Space group | Pccn |
| a/Å | 23.5961(4) |
| b/Å | 14.0616(3) |
| c/Å | 9.6760(2) |
| α/° | 90 |
| β/° | 90 |
| γ/° | 90 |
| Volume/Å^3^ | 3210.49(11) |
| Z | 8 |
| ρ_calc_g/cm^3^ | 1.172 |
| μ/mm^‑1^ | 0.642 |
| F(000) | 1200.0 |
| Crystal size/mm^3^ | 0.221 × 0.036 × 0.016 |
| Radiation | Cu Kα (λ = 1.54184) |
| 2Θ range for data collection/° | 7.494 to 150.792 |
| Index ranges | -29 ≤ h ≤ 29, -9 ≤ k ≤ 17, -12 ≤ l ≤ 11 |
| Reflections collected | 15614 |
| Independent reflections | 3227 [R_int_ = 0.0277, R_sigma_ = 0.0235] |
| Data/restraints/parameters | 3227/0/196 |
| Goodness-of-fit on F^2^ | 1.038 |
| Final R indexes [I>=2σ (I)] | R_1_ = 0.0420, wR_2_ = 0.1031 |
| Final R indexes [all data] | R_1_ = 0.0492, wR_2_ = 0.1072 |
| Largest diff. peak/hole / e Å^-3^ | 0.20/-0.24 |

Crystallographic files (CDCC: 2392295 (**5**))


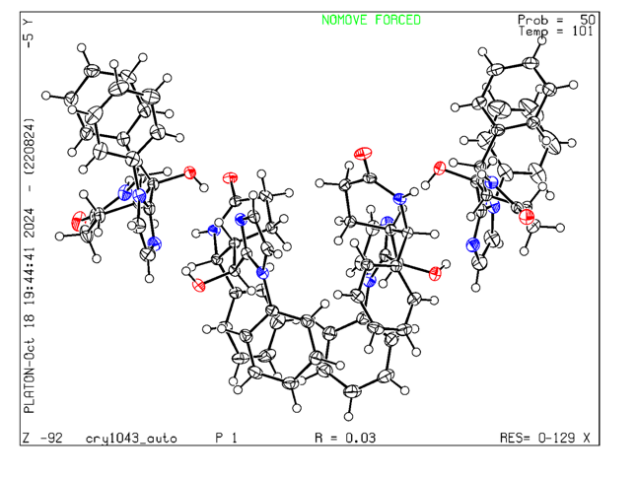


| **Table S3 Crystal data and structure refinement for cry1043_auto.** | |
| --- | --- |
| Identification code | cry1043_auto |
| Empirical formula | C_21_H_21_N_3_O_2_ |
| Formula weight | 347.41 |
| Temperature/K | 101(2) |
| Crystal system | triclinic |
| Space group | P1 |
| a/Å | 9.87228(10) |
| b/Å | 13.01294(13) |
| c/Å | 14.42469(12) |
| α/° | 94.0539(7) |
| β/° | 93.1954(7) |
| γ/° | 94.6894(8) |
| Volume/Å^3^ | 1838.75(3) |
| Z | 4 |
| ρ_calc_g/cm^3^ | 1.255 |
| μ/mm^‑1^ | 0.659 |
| F(000) | 736.0 |
| Crystal size/mm^3^ | 0.144 × 0.129 × 0.084 |
| Radiation | Cu Kα (λ = 1.54184) |
| 2Θ range for data collection/° | 6.836 to 159.636 |
| Index ranges | -12 ≤ h ≤ 12, -16 ≤ k ≤ 16, -18 ≤ l ≤ 18 |
| Reflections collected | 71055 |
| Independent reflections | 14608 [R_int_ = 0.0401, R_sigma_ = 0.0273] |
| Data/restraints/parameters | 14608/3/957 |
| Goodness-of-fit on F^2^ | 1.043 |
| Final R indexes [I>=2σ (I)] | R_1_ = 0.0296, wR_2_ = 0.0736 |
| Final R indexes [all data] | R_1_ = 0.0304, wR_2_ = 0.0742 |
| Largest diff. peak/hole / e Å^-3^ | 0.14/-0.18 |
| Flack parameter | 0.00(5) |

# Computational methods

All geometry optimizations of intermediates and transition states were achieved using spin-unrestricted uB3LYP^[73-74]^-D3^[75-78]^/def2SVP-CPCM^[79-80]^ method, in MeCN solvent using the CPCM solvent model^[81-85]^ with “opt=noeigen” and “guess=mix” keywords as implemented in Gaussian16.^[86]^ Frequency calculations were also conducted at the same level of theory to obtain vibrational frequencies to determine the identity of stationary points as intermediates (no imaginary frequencies) or transition states (only one imaginary frequency), as well as obtaining the thermochemistry: enthalpy (Δ*H*) and free energy (Δ*G*) at the temperature of 298 K. Also, extensive conformation was performed and only the lowest-energy species were shown and discussed. All structural figures were generated with CYLview.^[87]^ Distances in structural figures are shown in Å and energies are in kcal/mol. Noncovalent interaction (NCI) analysis, also known as reduce density gradient (RDG) method, was performed on Multiwfn to study the possible effect of noncovalent interaction in the relevant transition states.^[88]^ Extension distance of 0 Bohr, high quality grid (totally about 1728000 points) was set by default. Further visualization of the color-filled RDG isosurface was realized by VMD, where RDG isosurface and color range were set as 0.35-0.58 depending on the structures, and -0.035 to 0.2, respectively.^[89]^

As shown in Fig S10**,** the first step of the mechanism is presumably the excitation of **1a** under 370-nm-wavelength light irradiation followed by intersystem crossing (ISC) to reach the triplet excited state **^3^[1a]*****.^[90]^** Mulliken spin density analysis of optimized **^3^[1a]*** revealed that the spin density was primarily located on oxygen atom (as shown in red) consistent with selective O-H bond formation (vs. C-H bond formation initiated from acyl carbon) via HAT step. Furthermore, the [1,*5*]-HAT takes place through an energy barrier of 6.7 kcal/mol (from **1a***) via **TS1** to form a thermodynamically stable benzylic [1,*4*]-diradical **^3^B** (15.0 kcal/mol downhill from the excited state intermediate **^3^[1a]*** ). In turn, **^3^B** can then convert to open-shell singlet species **^1^B** by transversing the minimum energy crossing point (MECP, Δ*E*= 0.1 kcal/mol). In the next step, **^1^B** selectively undergoes an irreversible radical-radical coupling (Δ*G*^‡^ = 6.8 kcal/mol from intermediate **^1^B**) leading to **D** (downhill by 33.2 kcal/mol in energy). Our calculations indicate that the barrier for C-N bond cleavage to form imine enol intermediate **C** is higher in energy in comparison to C-C bond formation (^1^**TS2** vs ^1^**TS2’** Δ*G*^‡^ = 8.6 vs 6.8 kcal/mol) for the non-protonated analogs.

**Fig. S10**: Potential energy surface for the non-protonated system calculated at the UB3LYP-D3/def2-svp-CPCM (acetonitrile) level of theory.

**Fig. S11**: Potential energy surface for the protonated system calculated at the UB3LYP-D3/def2-svp-CPCM (acetonitrile) level of theory.

**Fig S12**: Electron density distribution map for ^1^1a and ^1^1aH^+^ calculated at the UB3LYP-D3/def2-svp-CPCM (acetonitrile) level of theory indicates the electron deficiency created upon the imidazole ring on protonation.

**Fig. S13**: Energies of the frontier orbitals (isovalue=0.05) (HOMO and LUMO) for both 1a and 1aH^+^ are calculated at the UB3LYP-D3/def2-svp-CPCM (acetonitrile) level of theory using Gaussian16. Our study shows that both HOMO and LUMO are stabilized on protonation; however, the degree of stabilization of LUMO (-3.2 eV for 1aH^+^ vs. -1.8 eV for 1a) is more than that of HOMO resulting in lowering the energy gap of excitation. Energies are reported in eV for HOMO and LUMO.

**Fig. S14:** Transition states for C-N bond cleavage and C-C bond formation (the protonated system vs. the non-protonated system) compared at different levels of theory.

**Fig. S15:** NCI analysis (RDG isovalue=0.58 for all except C’’H+, isovalue of NCI plot of C’’H+ is 0.50) for all possible lower energetic conformations of imine-enol intermediate in terms of H bonding calculated at the UB3LYP-D3/def2-svp-CPCM (acetonitrile) level of theory.

**Fig. S16**: NCI analysis (RDG isovalue =0.35) TS3H+ and TS4H+ calculated at the UB3LYP-D3/def2-svp-CPCM (acetonitrile) level of theory.

**Fig. S17**: Method dependence on the potential energy surface with Brønsted Acid reaction coordinate.

# Cartesian coordinates

**Table S4.** Cartesian coordinates (xyz format) and energies of all the structures involved in each reaction mechanism studied calculated at the CPCM(ACN) uB3LYP-d3/def2-svp level of theory.

**1a**

E(scf) = -934.044795741a.u.

ν_min_ = 13.7421cm^-1^

| C | -0.919017 | -3.038708 | -2.614941 |
| --- | --- | --- | --- |
| C | -1.441216 | -1.634342 | -2.900294 |
| N | -1.564819 | -0.988303 | -1.699863 |
| C | -1.068161 | -1.754567 | -0.572758 |
| C | -1.110315 | -3.211605 | -1.104272 |
| O | -1.694986 | -1.153154 | -3.994077 |
| C | -1.890386 | 0.419255 | -1.587796 |
| C | 0.368920 | -1.336848 | -0.218399 |
| C | 0.831101 | -1.713202 | 1.118898 |
| O | 1.074504 | -0.752649 | -1.028830 |
| N | 0.076402 | -2.325984 | 2.033265 |
| C | 0.873120 | -2.504907 | 3.114532 |
| C | 2.140476 | -1.992545 | 2.860166 |
| N | 2.105817 | -1.493030 | 1.593491 |
| C | 3.217529 | -0.854859 | 0.896250 |
| C | -3.022189 | 0.689246 | -0.615408 |
| C | -4.173837 | -0.114208 | -0.619739 |
| C | -5.226040 | 0.144962 | 0.262482 |
| C | -5.142463 | 1.214898 | 1.162318 |
| C | -3.997966 | 2.018324 | 1.175725 |
| C | -2.943270 | 1.752437 | 0.294706 |
| H | -1.447097 | -3.779401 | -3.231261 |
| H | 0.144887 | -3.060400 | -2.904754 |
| H | -1.708657 | -1.630847 | 0.310887 |
| H | -0.356109 | -3.850359 | -0.626103 |
| H | -2.101184 | -3.635990 | -0.884528 |
| H | -2.165371 | 0.749347 | -2.602100 |
| H | -0.992458 | 0.988361 | -1.292836 |
| H | 0.523149 | -2.984309 | 4.028363 |
| H | 3.038828 | -1.946206 | 3.471633 |
| H | 4.089566 | -0.848924 | 1.561553 |
| H | 3.453521 | -1.405861 | -0.023086 |
| H | 2.950878 | 0.173368 | 0.620812 |
| H | -4.237984 | -0.954146 | -1.316729 |
| H | -6.115666 | -0.490208 | 0.248621 |
| H | -5.964772 | 1.417375 | 1.853284 |
| H | -3.920629 | 2.851123 | 1.879559 |
| H | -2.045725 | 2.377279 | 0.317086 |


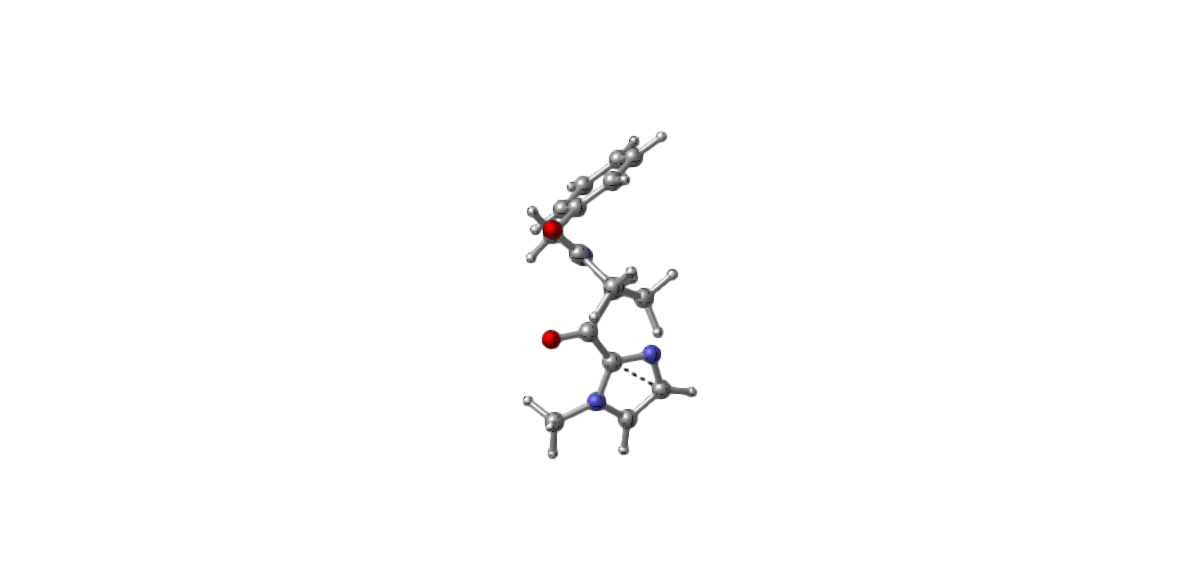


Zero-point correction= 0.310025 (Hartree/Particle)

Thermal correction to Energy= 0.328278

Thermal correction to Enthalpy= 0.329223

Thermal correction to Gibbs Free Energy= 0.259871

Sum of electronic and zero-point Energies= -933.734771

Sum of electronic and thermal Energies= -933.716517

Sum of electronic and thermal Enthalpies= -933.715573

Sum of electronic and thermal Free Energies= -933.784925

E(scf) = -933.939299505 a.u.

ν_min_ = 23.7201cm^-1^

| C | -0.841782 | -3.111480 | -2.548341 |
| --- | --- | --- | --- |
| C | -1.363530 | -1.686110 | -2.607999 |
| N | -1.212839 | -1.145426 | -1.339287 |
| C | -0.616166 | -2.046831 | -0.406378 |
| C | -0.823794 | -3.427787 | -1.050262 |
| O | -1.819843 | -1.080441 | -3.558958 |
| C | -1.559479 | 0.234355 | -1.019766 |
| C | 0.911830 | -1.701083 | -0.155347 |
| C | 1.214407 | -0.927522 | 0.996249 |
| O | 1.719833 | -2.090306 | -1.068002 |
| N | 0.317016 | -0.503670 | 1.909314 |
| C | 1.034479 | 0.174752 | 2.849993 |
| C | 2.375647 | 0.192330 | 2.522928 |
| N | 2.490610 | -0.498387 | 1.330621 |
| C | 3.728419 | -0.813748 | 0.642595 |
| C | -2.919693 | 0.326445 | -0.355049 |
| C | -4.083600 | 0.368845 | -1.138896 |
| C | -5.342409 | 0.413592 | -0.532602 |
| C | -5.448908 | 0.411518 | 0.863685 |
| C | -4.292088 | 0.361915 | 1.649276 |
| C | -3.031152 | 0.318005 | 1.043531 |
| H | -1.453586 | -3.781890 | -3.166474 |
| H | 0.183116 | -3.101435 | -2.960035 |
| H | -1.045983 | -1.920541 | 0.600814 |
| H | -0.049516 | -4.145757 | -0.755240 |
| H | -1.801944 | -3.815829 | -0.727100 |
| H | -1.551197 | 0.792242 | -1.965821 |
| H | -0.775618 | 0.634679 | -0.361706 |
| H | 0.558256 | 0.634234 | 3.716160 |
| H | 3.240901 | 0.629470 | 3.015428 |
| H | 3.600430 | -0.697482 | -0.442376 |
| H | 4.512556 | -0.123921 | 0.981885 |
| H | 4.049602 | -1.847931 | 0.847585 |
| H | -3.995795 | 0.360591 | -2.228717 |
| H | -6.243247 | 0.451135 | -1.150646 |
| H | -6.433114 | 0.448061 | 1.337882 |
| H | -4.370986 | 0.358325 | 2.739644 |
| H | -2.121729 | 0.264875 | 1.648383 |


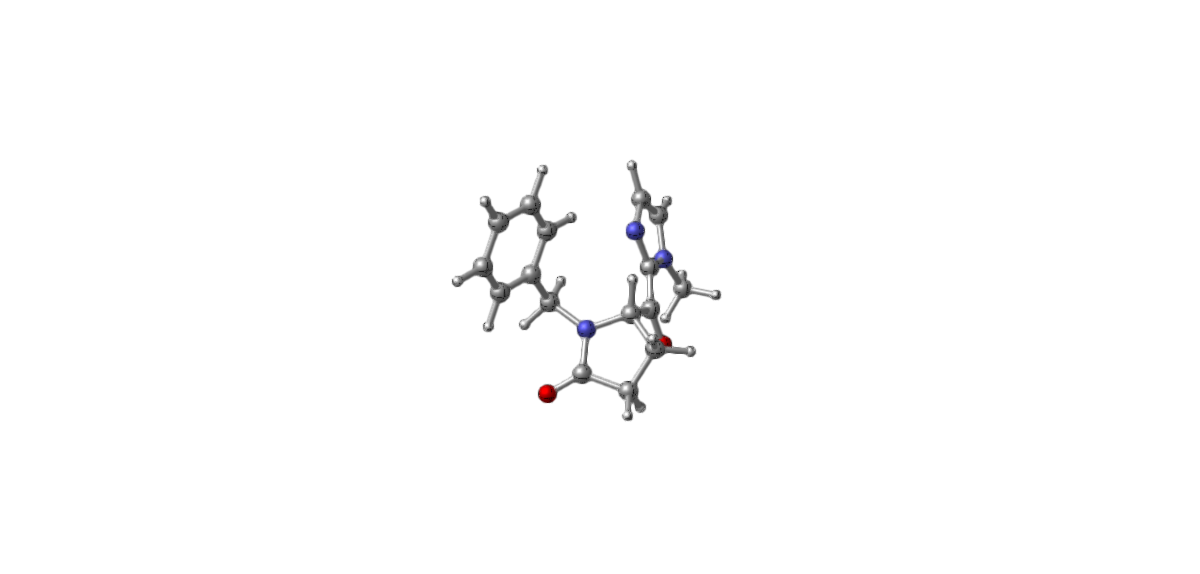


Zero-point correction= 0.306205 (Hartree/Particle)

Thermal correction to Energy= 0.324917

Thermal correction to Enthalpy= 0.325861

Thermal correction to Gibbs Free Energy= 0.255630

Sum of electronic and zero-point Energies= -933.633094

Sum of electronic and thermal Energies= -933.614383

Sum of electronic and thermal Enthalpies= -933.613439

Sum of electronic and thermal Free Energies= -933.683670

**2a**

E(scf) = -934.049967960 a.u.

ν_min_ = 25.9930cm^-1^

| C | -0.885415 | -3.626142 | -3.091770 |
| --- | --- | --- | --- |
| C | -2.217196 | -2.901229 | -3.175880 |
| N | -2.416073 | -1.858242 | -2.321758 |
| C | -0.297680 | -2.184062 | -1.067794 |
| C | 0.217359 | -2.879358 | -2.337649 |
| O | -3.097584 | -3.266889 | -3.949354 |
| C | -1.445411 | -1.205162 | -1.449362 |
| C | -0.765730 | -3.157457 | 0.009609 |
| C | -0.307618 | -2.881696 | 1.378304 |
| O | -1.523752 | -4.089345 | -0.235680 |
| N | 0.603402 | -1.966847 | 1.715592 |
| C | 0.710595 | -2.022121 | 3.065413 |
| C | -0.154761 | -2.984969 | 3.569900 |
| N | -0.793124 | -3.528326 | 2.495320 |
| C | -1.826212 | -4.556803 | 2.555558 |
| C | -2.120752 | -0.607558 | -0.224003 |
| C | -3.197319 | -1.252170 | 0.404970 |
| C | -3.741434 | -0.737767 | 1.586087 |
| C | -3.217156 | 0.429257 | 2.151950 |
| C | -2.149417 | 1.082136 | 1.525665 |
| C | -1.607476 | 0.566471 | 0.344992 |
| H | -1.109506 | -4.583551 | -2.597956 |
| H | -0.582854 | -3.861004 | -4.123330 |
| H | 0.501803 | -1.570722 | -0.634666 |
| H | 0.649064 | -2.098957 | -2.986333 |
| H | 1.038843 | -3.567684 | -2.085470 |
| H | -0.959749 | -0.379058 | -2.000645 |
| H | -3.314314 | -1.394196 | -2.431364 |
| H | 1.390713 | -1.381520 | 3.626210 |
| H | -0.360454 | -3.312243 | 4.586651 |
| H | -2.757622 | -4.178648 | 2.113920 |
| H | -1.514233 | -5.444371 | 1.992033 |
| H | -1.995356 | -4.819340 | 3.607142 |
| H | -3.598206 | -2.171969 | -0.025203 |
| H | -4.576350 | -1.253823 | 2.066989 |
| H | -3.641258 | 0.831065 | 3.075603 |
| H | -1.737503 | 1.997709 | 1.957639 |
| H | -0.769084 | 1.077347 | -0.136898 |


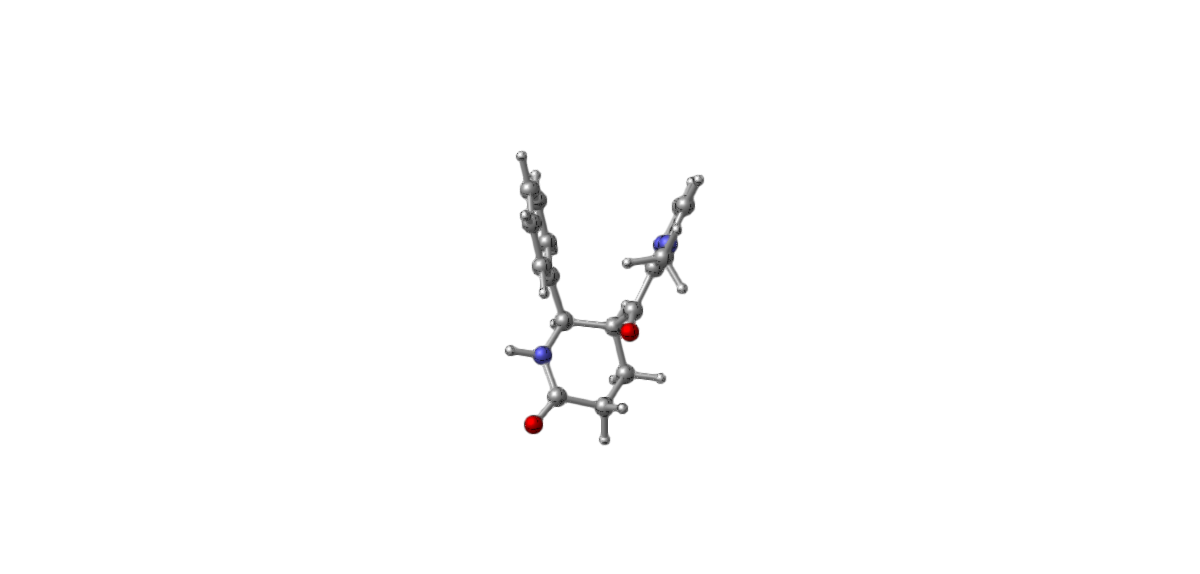


Zero-point correction= 0.311430 (Hartree/Particle)

Thermal correction to Energy= 0.329218

Thermal correction to Enthalpy= 0.330163

Thermal correction to Gibbs Free Energy= 0.264086

Sum of electronic and zero-point Energies= -933.738538

Sum of electronic and thermal Energies= -933.720750

Sum of electronic and thermal Enthalpies= -933.719805

Sum of electronic and thermal Free Energies= -933.785882

E(scf) = -933.966004327a.u.

ν_min_ = 21.0895cm^-1^

| C | -1.890313 | -3.724732 | -3.327117 |
| --- | --- | --- | --- |
| C | -2.197842 | -2.239258 | -3.313198 |
| N | -1.868189 | -1.740494 | -2.052409 |
| C | -1.308387 | -2.804258 | -1.163493 |
| C | -0.889592 | -3.888992 | -2.186763 |
| O | -2.658928 | -1.563902 | -4.215060 |
| C | -1.889541 | -0.375551 | -1.813285 |
| C | -0.192345 | -2.311540 | -0.304329 |
| C | -0.039473 | -2.756909 | 1.030586 |
| O | 0.732246 | -1.485836 | -0.851236 |
| N | -0.823919 | -3.698372 | 1.592273 |
| C | -0.395462 | -3.820563 | 2.877204 |
| C | 0.658208 | -2.957887 | 3.123073 |
| N | 0.886485 | -2.282336 | 1.945430 |
| C | 1.846578 | -1.202951 | 1.765456 |
| C | -1.943804 | 0.262434 | -0.544699 |
| C | -2.245400 | -0.404638 | 0.679503 |
| C | -2.196700 | 0.277448 | 1.895120 |
| C | -1.872571 | 1.638771 | 1.942016 |
| C | -1.616478 | 2.327531 | 0.740860 |
| C | -1.654499 | 1.660727 | -0.475002 |
| H | -2.830178 | -4.272623 | -3.138052 |
| H | -1.519018 | -4.028274 | -4.315420 |
| H | -2.099588 | -3.202680 | -0.512419 |
| H | 0.129127 | -3.667363 | -2.541066 |
| H | -0.887736 | -4.882267 | -1.719445 |
| H | -1.893439 | 0.223285 | -2.726259 |
| H | 0.306006 | -0.897291 | -1.502688 |
| H | -0.852880 | -4.520333 | 3.577112 |
| H | 1.251884 | -2.769406 | 4.014418 |
| H | 2.380748 | -1.048128 | 2.711391 |
| H | 2.568443 | -1.448263 | 0.975518 |
| H | 1.330075 | -0.273928 | 1.485226 |
| H | -2.558280 | -1.446583 | 0.677228 |
| H | -2.425618 | -0.262122 | 2.817900 |
| H | -1.833726 | 2.165323 | 2.898478 |
| H | -1.380804 | 3.394754 | 0.764514 |
| H | -1.438462 | 2.201155 | -1.400717 |


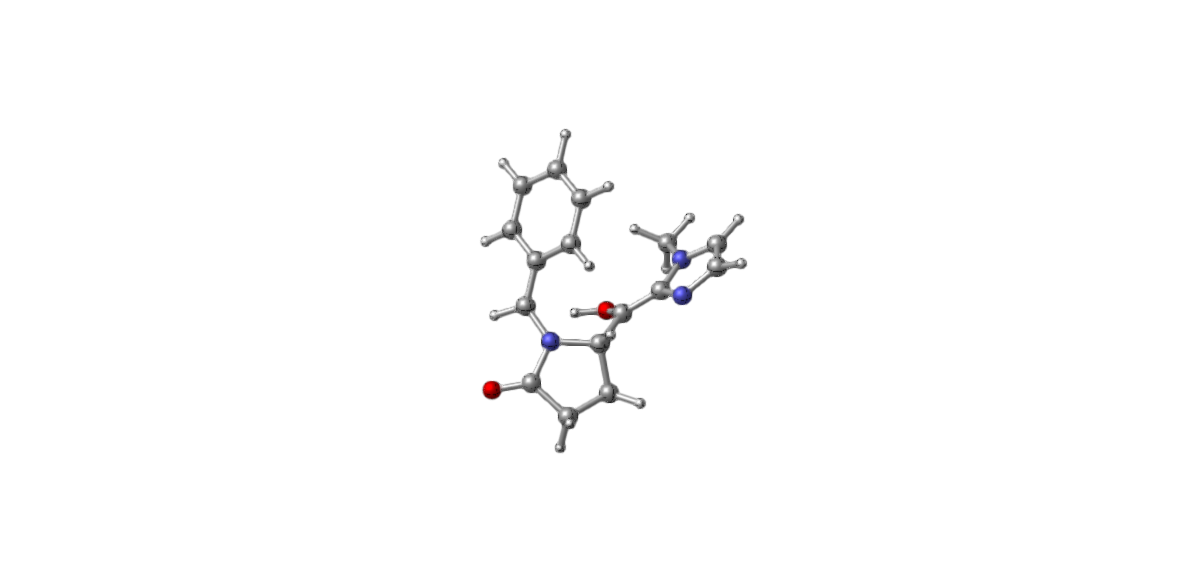


Zero-point correction= 0.306451 (Hartree/Particle)

Thermal correction to Energy= 0.325085

Thermal correction to Enthalpy= 0.326030

Thermal correction to Gibbs Free Energy= 0.258430

Sum of electronic and zero-point Energies= -933.659553

Sum of electronic and thermal Energies= -933.640919

Sum of electronic and thermal Enthalpies= -933.639975

Sum of electronic and thermal Free Energies= -933.707575

E(scf) = -933.964648282 a.u.

ν_min_ = 17.2619cm^-1^

| C | -1.107221 | -3.257791 | -3.373070 |
| --- | --- | --- | --- |
| C | -1.772444 | -1.895674 | -3.366522 |
| N | -1.688876 | -1.379267 | -2.072497 |
| C | -0.945165 | -2.279579 | -1.149053 |
| C | -0.220876 | -3.238937 | -2.130153 |
| O | -2.317326 | -1.328656 | -4.296288 |
| C | -2.211570 | -0.126720 | -1.787426 |
| C | -0.006294 | -1.525358 | -0.260403 |
| C | 0.111946 | -1.821191 | 1.115125 |
| O | 0.846012 | -0.627705 | -0.825736 |
| N | -0.607350 | -2.782812 | 1.734027 |
| C | -0.250432 | -2.730671 | 3.043979 |
| C | 0.694537 | -1.741281 | 3.253125 |
| N | 0.926632 | -1.163272 | 2.025436 |
| C | 1.818141 | -0.038842 | 1.780602 |
| C | -2.606379 | 0.407033 | -0.529244 |
| C | -2.784042 | -0.343743 | 0.670987 |
| C | -3.169645 | 0.280180 | 1.853407 |
| C | -3.407068 | 1.662611 | 1.898155 |
| C | -3.265943 | 2.418189 | 0.722257 |
| C | -2.880030 | 1.808686 | -0.465069 |
| H | -1.903152 | -4.019766 | -3.301793 |
| H | -0.574459 | -3.421337 | -4.320161 |
| H | -1.645298 | -2.843337 | -0.517562 |
| H | 0.765550 | -2.817236 | -2.377128 |
| H | -0.066024 | -4.223122 | -1.669414 |
| H | -2.354038 | 0.478372 | -2.685099 |
| H | 0.480383 | -0.312687 | -1.666834 |
| H | -0.679417 | -3.401509 | 3.788787 |
| H | 1.210652 | -1.405948 | 4.149564 |
| H | 2.269535 | 0.264248 | 2.733705 |
| H | 2.612561 | -0.315585 | 1.074575 |
| H | 1.261854 | 0.808829 | 1.357341 |
| H | -2.629338 | -1.420257 | 0.681362 |
| H | -3.291157 | -0.323701 | 2.756755 |
| H | -3.706249 | 2.143170 | 2.832588 |
| H | -3.458506 | 3.494296 | 0.738057 |
| H | -2.768151 | 2.409740 | -1.371682 |


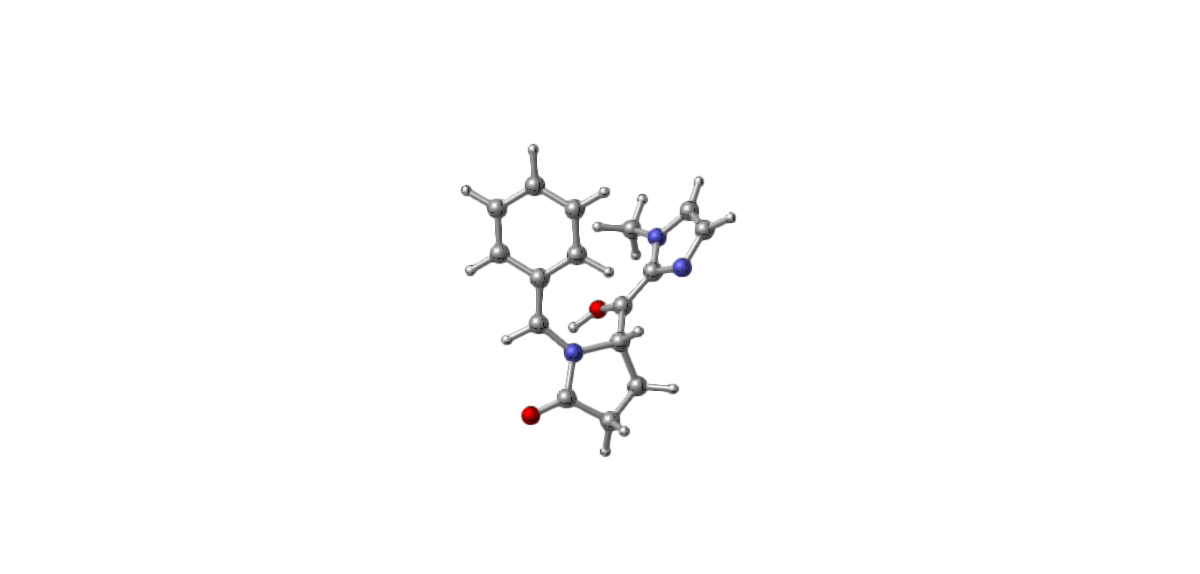


Zero-point correction= 0.306549 (Hartree/Particle)

Thermal correction to Energy= 0.325343

Thermal correction to Enthalpy= 0.326287

Thermal correction to Gibbs Free Energy= 0.257074

Sum of electronic and zero-point Energies= -933.658099

Sum of electronic and thermal Energies= -933.639305

Sum of electronic and thermal Enthalpies= -933.638361

Sum of electronic and thermal Free Energies= -933.707574

**C**

E(scf) = -933.998052811a.u.

ν_min_ = 26.9669cm^-1^

| C | -1.182515 | -3.324685 | -3.177958 |
| --- | --- | --- | --- |
| C | -2.054572 | -2.080648 | -3.131276 |
| N | -2.265832 | -1.562339 | -1.851615 |
| C | 0.133624 | -3.157738 | -0.986385 |
| C | 0.195342 | -3.186577 | -2.486427 |
| O | -2.587486 | -1.614104 | -4.122777 |
| C | -2.048054 | -0.326233 | -1.601712 |
| C | 0.389817 | -2.089971 | -0.202599 |
| C | 0.194059 | -2.126446 | 1.255624 |
| O | 0.782981 | -0.868609 | -0.667397 |
| N | -0.286826 | -3.183499 | 1.903242 |
| C | -0.356943 | -2.825073 | 3.218027 |
| C | 0.086939 | -1.529040 | 3.378057 |
| N | 0.444271 | -1.088668 | 2.124408 |
| C | 0.918429 | 0.263243 | 1.844194 |
| C | -2.287602 | 0.278997 | -0.287525 |
| C | -2.636941 | -0.509744 | 0.825988 |
| C | -2.824886 | 0.083497 | 2.072514 |
| C | -2.674303 | 1.469672 | 2.222112 |
| C | -2.331247 | 2.261717 | 1.120556 |
| C | -2.131794 | 1.668063 | -0.127947 |
| H | -1.746416 | -4.143347 | -2.697133 |
| H | -1.056269 | -3.584223 | -4.238826 |
| H | -0.205208 | -4.061964 | -0.477613 |
| H | 0.713238 | -2.302272 | -2.899358 |
| H | 0.803759 | -4.049532 | -2.807052 |
| H | -1.640276 | 0.345205 | -2.379726 |
| H | 0.924232 | -0.908644 | -1.623302 |
| H | -0.720324 | -3.506151 | 3.987538 |
| H | 0.177292 | -0.890316 | 4.253092 |
| H | 1.036163 | 0.790554 | 2.798793 |
| H | 1.881202 | 0.234487 | 1.320566 |
| H | 0.193946 | 0.800223 | 1.219837 |
| H | -2.726317 | -1.589757 | 0.701279 |
| H | -3.074395 | -0.535944 | 2.936537 |
| H | -2.817260 | 1.931011 | 3.202418 |
| H | -2.209725 | 3.341064 | 1.237597 |
| H | -1.850006 | 2.281868 | -0.987841 |


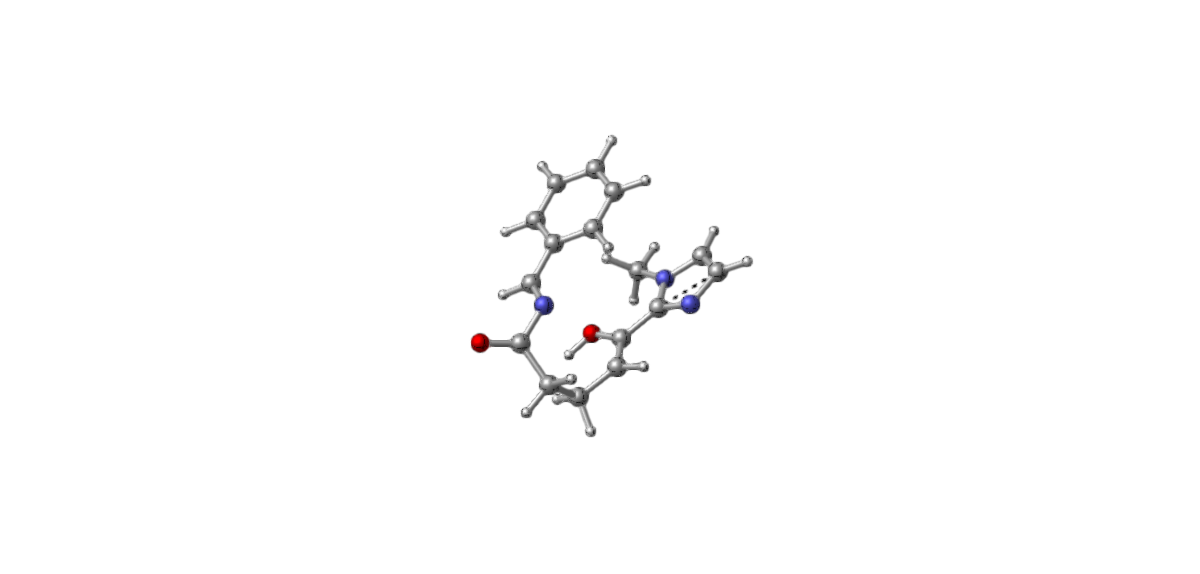


Zero-point correction= 0.307632 (Hartree/Particle)

Thermal correction to Energy= 0.326551

Thermal correction to Enthalpy= 0.327495

Thermal correction to Gibbs Free Energy= 0.259401

Sum of electronic and zero-point Energies= -933.690421

Sum of electronic and thermal Energies= -933.671502

Sum of electronic and thermal Enthalpies= -933.670557

Sum of electronic and thermal Free Energies= -933.738652

**D**

E(scf) = -933.999578498 a.u.

ν_min_ = 20.6894cm^-1^

| C | -1.403231 | -3.222650 | -3.481534 |
| --- | --- | --- | --- |
| C | -2.067932 | -1.905241 | -3.058780 |
| N | -1.845153 | -1.777650 | -1.704776 |
| C | -0.791801 | -2.686480 | -1.223104 |
| C | -0.232139 | -3.362503 | -2.484886 |
| O | -2.622533 | -1.085325 | -3.766117 |
| C | -1.301136 | -0.571167 | -1.051404 |
| C | -0.066103 | -1.469794 | -0.548239 |
| C | 0.082827 | -1.598927 | 0.937108 |
| O | 1.178609 | -1.097143 | -1.081549 |
| N | -0.385015 | -2.598637 | 1.666176 |
| C | -0.095755 | -2.283064 | 2.968725 |
| C | 0.553640 | -1.069232 | 3.024754 |
| N | 0.659062 | -0.634494 | 1.722412 |
| C | 1.318281 | 0.594251 | 1.300799 |
| C | -2.160485 | 0.092546 | -0.010761 |
| C | -3.065693 | -0.651328 | 0.762179 |
| C | -3.800582 | -0.036635 | 1.778906 |
| C | -3.641493 | 1.330620 | 2.035722 |
| C | -2.750063 | 2.081453 | 1.262399 |
| C | -2.017851 | 1.465195 | 0.242650 |
| H | -2.127951 | -4.047770 | -3.373649 |
| H | -1.107102 | -3.168813 | -4.537644 |
| H | -1.152914 | -3.405451 | -0.475383 |
| H | 0.649268 | -2.812941 | -2.851328 |
| H | 0.073686 | -4.402447 | -2.309593 |
| H | -0.969573 | 0.174123 | -1.793883 |
| H | 1.066687 | -0.752317 | -1.978341 |
| H | -0.368314 | -2.935404 | 3.797838 |
| H | 0.945623 | -0.486122 | 3.854810 |
| H | 1.298340 | 1.310341 | 2.132360 |
| H | 2.358878 | 0.398749 | 1.005764 |
| H | 0.791870 | 1.030779 | 0.445696 |
| H | -3.189489 | -1.717311 | 0.562086 |
| H | -4.501446 | -0.627153 | 2.374531 |
| H | -4.216604 | 1.810323 | 2.831726 |
| H | -2.628844 | 3.151692 | 1.447668 |
| H | -1.331919 | 2.060295 | -0.366839 |


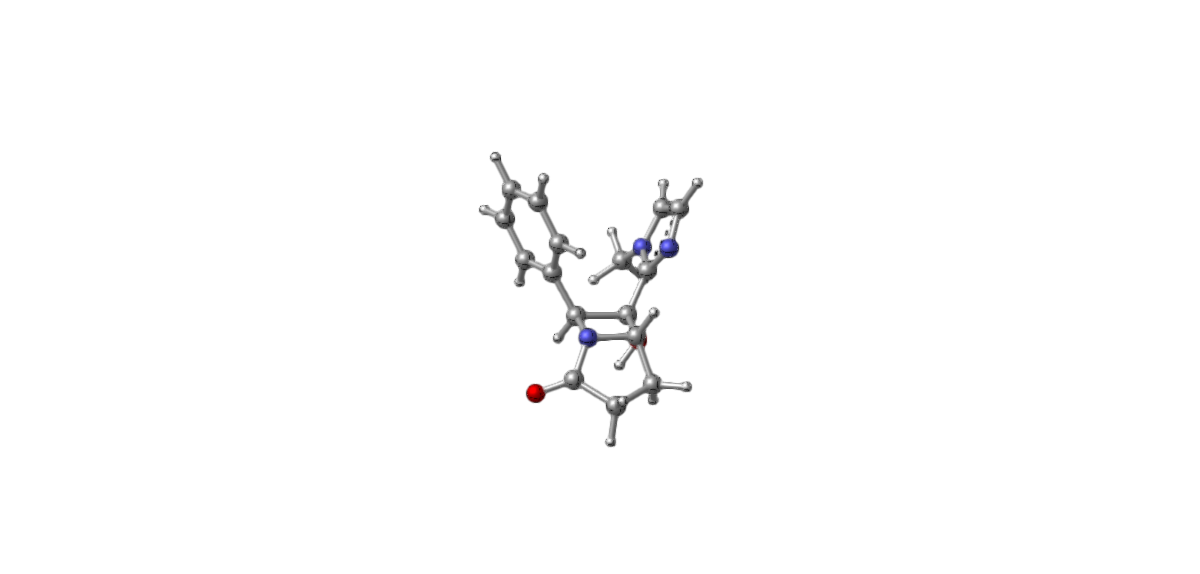


Zero-point correction= 0.310026 (Hartree/Particle)

Thermal correction to Energy= 0.327805

Thermal correction to Enthalpy= 0.328749

Thermal correction to Gibbs Free Energy= 0.262963

Sum of electronic and zero-point Energies= -933.689552

Sum of electronic and thermal Energies= -933.671773

Sum of electronic and thermal Enthalpies= -933.670829

Sum of electronic and thermal Free Energies= -933.736616

**^3^B’**

E(scf) = -933.963568494 a.u.

ν_min_ = 20.2057cm^-1^

| C | -0.096616 | -2.918467 | -3.063504 |
| --- | --- | --- | --- |
| C | -1.046675 | -1.747409 | -3.226073 |
| N | -1.452161 | -1.336021 | -1.948788 |
| C | -0.857406 | -2.162657 | -0.878465 |
| C | -0.378858 | -3.425007 | -1.650899 |
| O | -1.394294 | -1.208655 | -4.260984 |
| C | -2.260026 | -0.222376 | -1.806654 |
| C | 0.244393 | -1.420924 | -0.168820 |
| C | 0.486636 | -1.626272 | 1.206749 |
| O | 1.127305 | -0.688360 | -0.902172 |
| N | -0.300049 | -2.402103 | 1.985594 |
| C | 0.222547 | -2.324569 | 3.237615 |
| C | 1.338790 | -1.506834 | 3.250040 |
| N | 1.508068 | -1.062912 | 1.957973 |
| C | 2.556989 | -0.158820 | 1.508819 |
| C | -2.817666 | 0.384870 | -0.648309 |
| C | -2.704218 | -0.055501 | 0.706995 |
| C | -3.307031 | 0.654337 | 1.742134 |
| C | -4.046855 | 1.819765 | 1.494705 |
| C | -4.179065 | 2.270874 | 0.170359 |
| C | -3.583693 | 1.575588 | -0.872635 |
| H | -0.261659 | -3.656348 | -3.860433 |
| H | 0.934045 | -2.538066 | -3.170748 |
| H | -1.615949 | -2.447767 | -0.142788 |
| H | 0.486444 | -3.886569 | -1.158473 |
| H | -1.196308 | -4.161740 | -1.667030 |
| H | -2.477388 | 0.231242 | -2.775279 |
| H | 0.680787 | -0.316944 | -1.678894 |
| H | -0.216957 | -2.854623 | 4.082926 |
| H | 2.013170 | -1.209954 | 4.049710 |
| H | 2.123536 | 0.758009 | 1.086305 |
| H | 3.187272 | 0.101375 | 2.368525 |
| H | 3.176329 | -0.633848 | 0.736060 |
| H | -2.143733 | -0.948287 | 0.969773 |
| H | -3.195139 | 0.288124 | 2.766436 |
| H | -4.513338 | 2.366918 | 2.317188 |
| H | -4.753408 | 3.176025 | -0.044593 |
| H | -3.695512 | 1.942499 | -1.896596 |


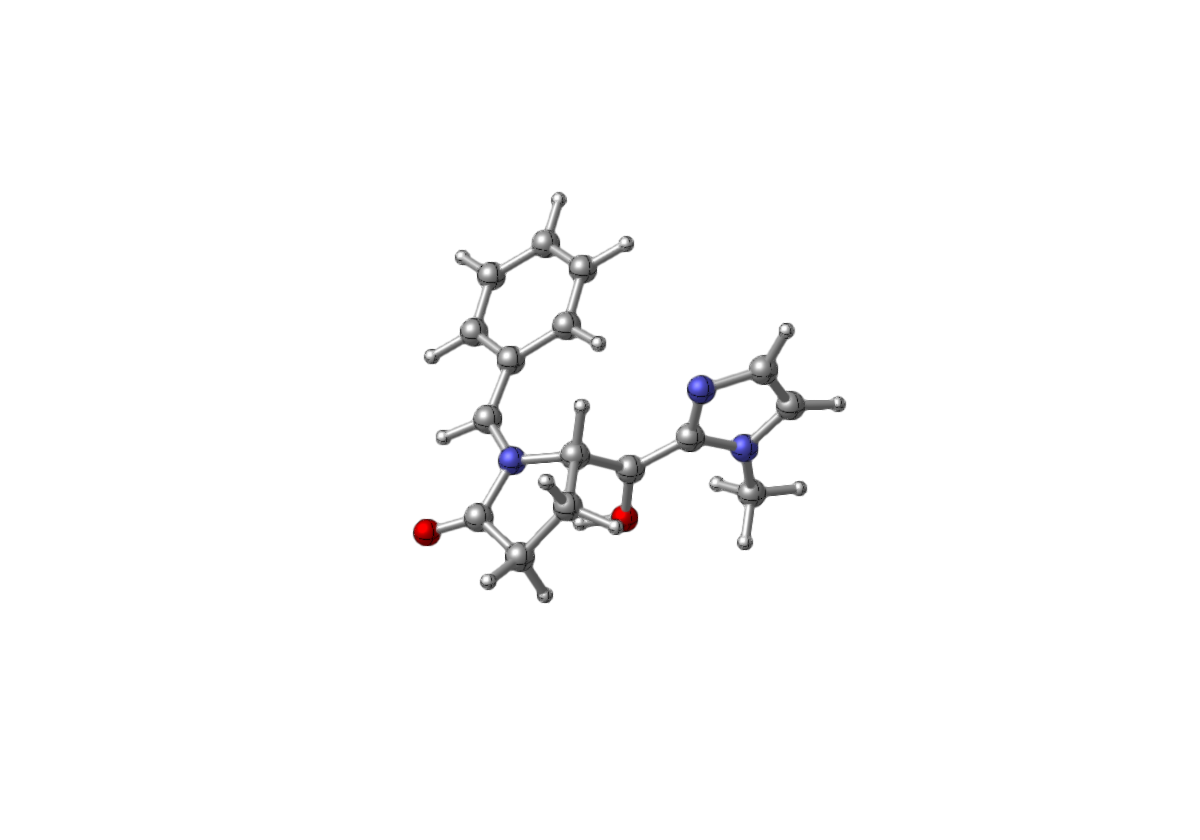


Zero-point correction= 0.306860 (Hartree/Particle)

Thermal correction to Energy= 0.325583

Thermal correction to Enthalpy= 0.326527

Thermal correction to Gibbs Free Energy= 0.257286

Sum of electronic and zero-point Energies= -933.656708

Sum of electronic and thermal Energies= -933.637986

Sum of electronic and thermal Enthalpies= -933.637042

Sum of electronic and thermal Free Energies= -933.7062

E(scf) = -933.926607668 a.u.

ν_min_ = -490.5493cm^-1^

| C | -0.485368 | -3.290868 | -2.350337 |
| --- | --- | --- | --- |
| C | -1.136218 | -1.991465 | -2.776667 |
| N | -1.500029 | -1.298576 | -1.615735 |
| C | -1.002740 | -1.932673 | -0.406930 |
| C | -0.786227 | -3.396512 | -0.853546 |
| O | -1.312214 | -1.562105 | -3.897462 |
| C | -1.713033 | 0.099936 | -1.631404 |
| C | 0.268632 | -1.171450 | 0.082216 |
| C | 1.034752 | -1.730478 | 1.151252 |
| O | 0.448406 | 0.086669 | -0.271373 |
| N | 0.790051 | -2.910955 | 1.749993 |
| C | 1.748892 | -3.049938 | 2.710644 |
| C | 2.593529 | -1.959134 | 2.717601 |
| N | 2.141004 | -1.115916 | 1.723058 |
| C | 2.736246 | 0.160666 | 1.365118 |
| C | -2.826968 | 0.644235 | -0.789355 |
| C | -3.881738 | -0.169137 | -0.341797 |
| C | -4.917551 | 0.375197 | 0.422318 |
| C | -4.911047 | 1.735379 | 0.751053 |
| C | -3.863519 | 2.552491 | 0.307097 |
| C | -2.827884 | 2.010434 | -0.455532 |
| H | -0.855762 | -4.128377 | -2.957598 |
| H | 0.594655 | -3.189459 | -2.553770 |
| H | -1.752157 | -1.868155 | 0.400298 |
| H | -0.004520 | -3.879172 | -0.261081 |
| H | -1.724756 | -3.948408 | -0.691432 |
| H | -1.706370 | 0.475021 | -2.666170 |
| H | -0.684630 | 0.458253 | -1.124036 |
| H | 1.793866 | -3.926010 | 3.358415 |
| H | 3.459737 | -1.711003 | 3.326315 |
| H | 2.053905 | 0.992694 | 1.590375 |
| H | 3.664143 | 0.289886 | 1.937573 |
| H | 2.969848 | 0.190820 | 0.291654 |
| H | -3.898915 | -1.227584 | -0.610693 |
| H | -5.736430 | -0.266019 | 0.757840 |
| H | -5.720747 | 2.158859 | 1.350322 |
| H | -3.852720 | 3.615470 | 0.560170 |
| H | -2.006718 | 2.648376 | -0.793722 |


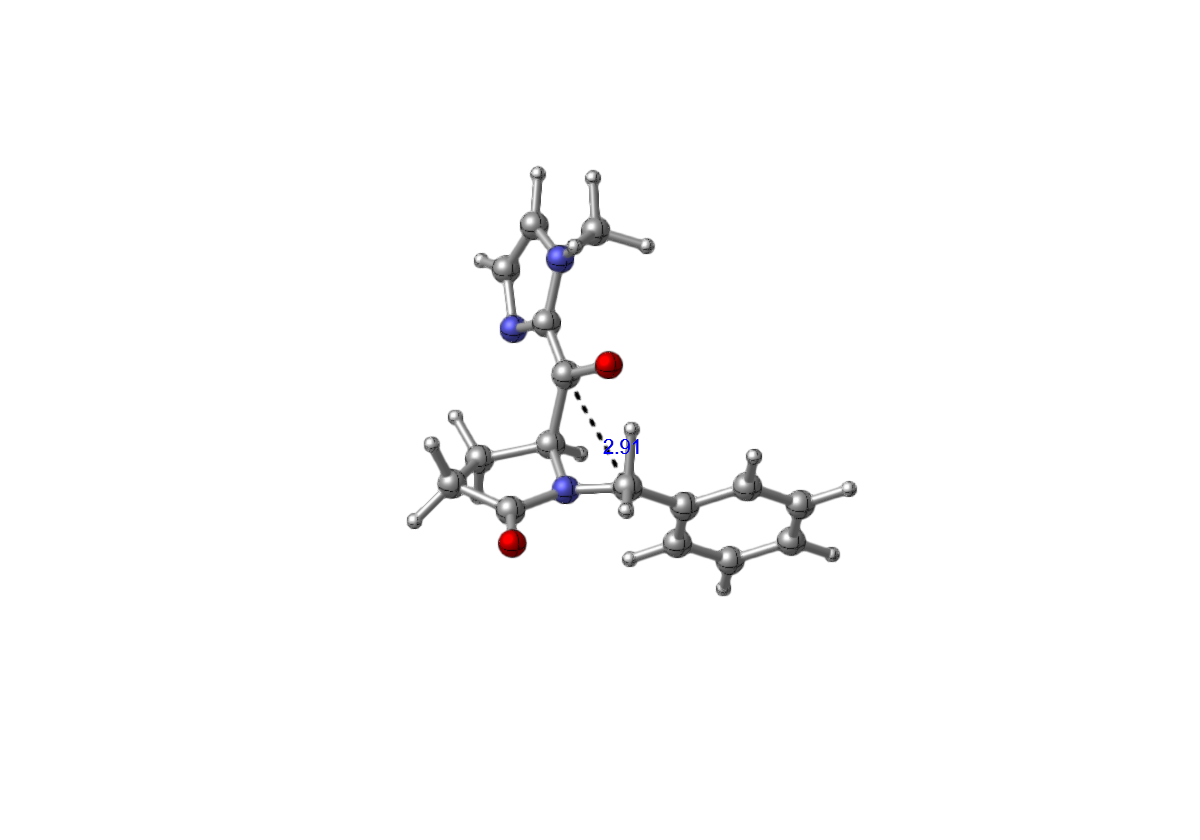


Zero-point correction= 0.302705 (Hartree/Particle)

Thermal correction to Energy= 0.320586

Thermal correction to Enthalpy= 0.321530

Thermal correction to Gibbs Free Energy= 0.253556

Sum of electronic and zero-point Energies= -933.623903

Sum of electronic and thermal Energies= -933.606022

Sum of electronic and thermal Enthalpies= -933.605078

Sum of electronic and thermal Free Energies= -933.673052

**TS2**

E(scf) = -933.953161835 a.u.

ν_min_ = -649.5272cm^-1^

| C | -1.111335 | -3.418793 | -3.319334 |
| --- | --- | --- | --- |
| C | -1.916498 | -2.141487 | -3.199696 |
| N | -1.814361 | -1.617302 | -1.914890 |
| C | -0.633471 | -2.707332 | -1.019994 |
| C | -0.003723 | -3.285527 | -2.282226 |
| O | -2.590348 | -1.635496 | -4.080728 |
| C | -1.871283 | -0.287948 | -1.717331 |
| C | 0.125258 | -1.894932 | -0.136457 |
| C | 0.107827 | -2.064584 | 1.292276 |
| O | 0.794813 | -0.842154 | -0.656962 |
| N | -0.326522 | -3.174901 | 1.901378 |
| C | -0.215315 | -2.934983 | 3.237015 |
| C | 0.304298 | -1.671694 | 3.456722 |
| N | 0.515003 | -1.122145 | 2.214725 |
| C | 0.945801 | 0.247481 | 1.965863 |
| C | -2.178462 | 0.284411 | -0.439216 |
| C | -2.556704 | -0.526425 | 0.660971 |
| C | -2.776180 | 0.038582 | 1.918617 |
| C | -2.642482 | 1.417790 | 2.108379 |
| C | -2.285058 | 2.238777 | 1.023513 |
| C | -2.051293 | 1.684526 | -0.228866 |
| H | -1.777915 | -4.270476 | -3.094989 |
| H | -0.754182 | -3.540596 | -4.350606 |
| H | -1.280405 | -3.416184 | -0.494427 |
| H | 0.784053 | -2.607874 | -2.646479 |
| H | 0.469178 | -4.249511 | -2.038134 |
| H | -1.677463 | 0.367854 | -2.576335 |
| H | 0.232383 | -0.399958 | -1.334371 |
| H | -0.498176 | -3.674597 | 3.986245 |
| H | 0.540800 | -1.129532 | 4.369154 |
| H | 1.073297 | 0.752787 | 2.931280 |
| H | 1.893754 | 0.265435 | 1.413395 |
| H | 0.186607 | 0.781833 | 1.378692 |
| H | -2.695786 | -1.597020 | 0.513439 |
| H | -3.055819 | -0.604957 | 2.756187 |
| H | -2.815531 | 1.856987 | 3.093703 |
| H | -2.183085 | 3.317353 | 1.167289 |
| H | -1.757076 | 2.323669 | -1.065893 |


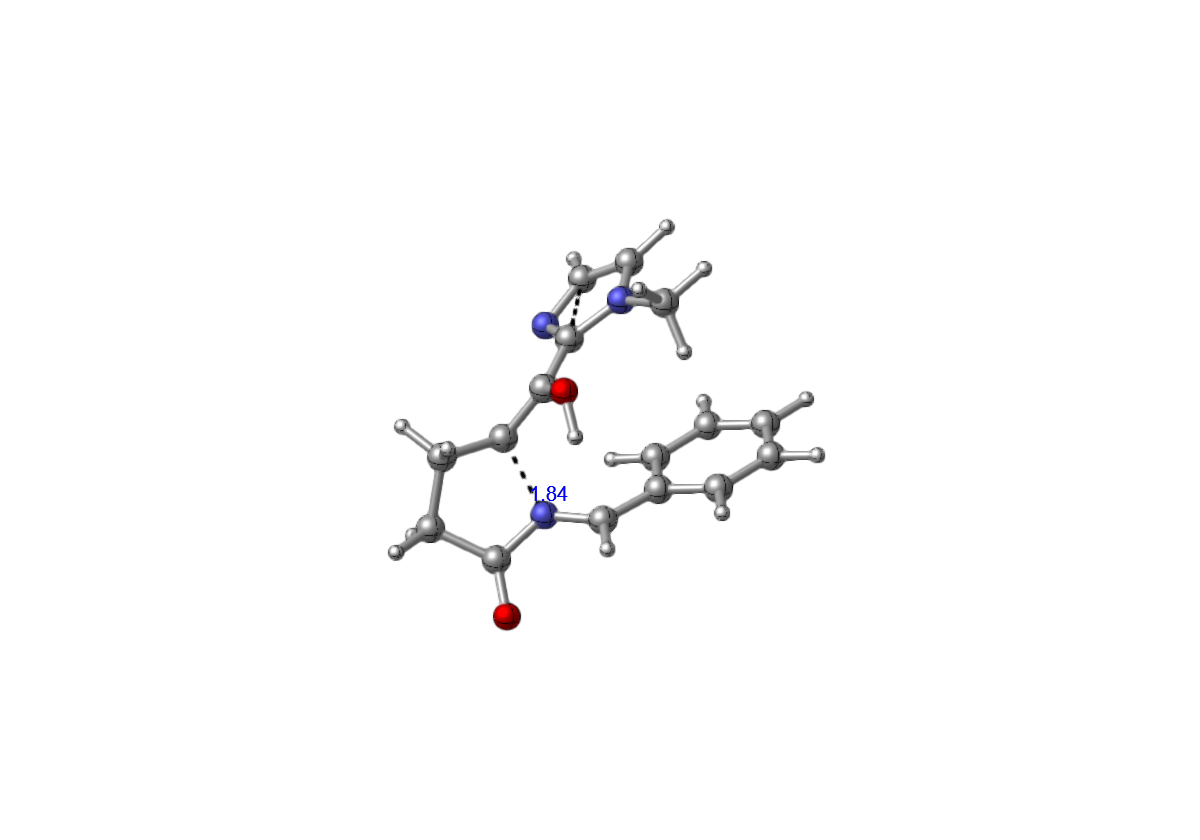


Zero-point correction= 0.305173 (Hartree/Particle)

Thermal correction to Energy= 0.323157

Thermal correction to Enthalpy= 0.324101

Thermal correction to Gibbs Free Energy= 0.259319

Sum of electronic and zero-point Energies= -933.647989

Sum of electronic and thermal Energies= -933.630005

Sum of electronic and thermal Enthalpies= -933.629061

Sum of electronic and thermal Free Energies= -933.693843

UB3LYP-D3/def2TZVPP-CPCM(ACN)

E(scf)= -935.447073459

UM062X-D3/def2TZVPP-CPCM(ACN)

E(scf)= -935.015016948

UTPSSH/def2TZVPP-CPCM(ACN)/

E(scf)= -935.457445623

UM06-D3/def2TZVPP-CPCM(ACN)

E(scf)= -934.298802056

**TS2’**

E(scf) = -933.954500042 a.u.

ν_min_ = -353.8910cm^-1^

| C | -1.386883 | -3.230818 | -3.404495 |
| --- | --- | --- | --- |
| C | -1.915956 | -1.808288 | -3.223183 |
| N | -1.680972 | -1.464809 | -1.918316 |
| C | -0.861049 | -2.423621 | -1.175760 |
| C | -0.369468 | -3.408048 | -2.263352 |
| O | -2.441954 | -1.091447 | -4.059784 |
| C | -1.707756 | -0.156177 | -1.373243 |
| C | 0.171655 | -1.558884 | -0.466922 |
| C | 0.201357 | -1.484209 | 0.950975 |
| O | 1.262481 | -1.121746 | -1.145506 |
| N | -0.611612 | -2.190128 | 1.758682 |
| C | -0.340929 | -1.770481 | 3.021059 |
| C | 0.641961 | -0.794038 | 3.006026 |
| N | 0.987008 | -0.613911 | 1.690681 |
| C | 1.925448 | 0.381525 | 1.191346 |
| C | -2.434027 | 0.183440 | -0.192947 |
| C | -3.250289 | -0.747172 | 0.505280 |
| C | -3.899779 | -0.384025 | 1.682867 |
| C | -3.753845 | 0.905350 | 2.212299 |
| C | -2.959426 | 1.843244 | 1.530104 |
| C | -2.316703 | 1.495549 | 0.348025 |
| H | -2.236165 | -3.928938 | -3.309399 |
| H | -0.968760 | -3.353741 | -4.413390 |
| H | -1.447069 | -2.938279 | -0.401679 |
| H | 0.635291 | -3.116447 | -2.606632 |
| H | -0.302627 | -4.437498 | -1.886864 |
| H | -1.338192 | 0.639778 | -2.027219 |
| H | 1.039236 | -0.953952 | -2.073843 |
| H | -0.854574 | -2.178377 | 3.891618 |
| H | 1.113165 | -0.225334 | 3.804388 |
| H | 2.263910 | 0.998183 | 2.033237 |
| H | 2.791264 | -0.098235 | 0.716544 |
| H | 1.433622 | 1.023328 | 0.447081 |
| H | -3.380138 | -1.751918 | 0.100268 |
| H | -4.525197 | -1.117166 | 2.199487 |
| H | -4.258502 | 1.181801 | 3.141173 |
| H | -2.846323 | 2.854656 | 1.929904 |
| H | -1.696852 | 2.230203 | -0.173808 |


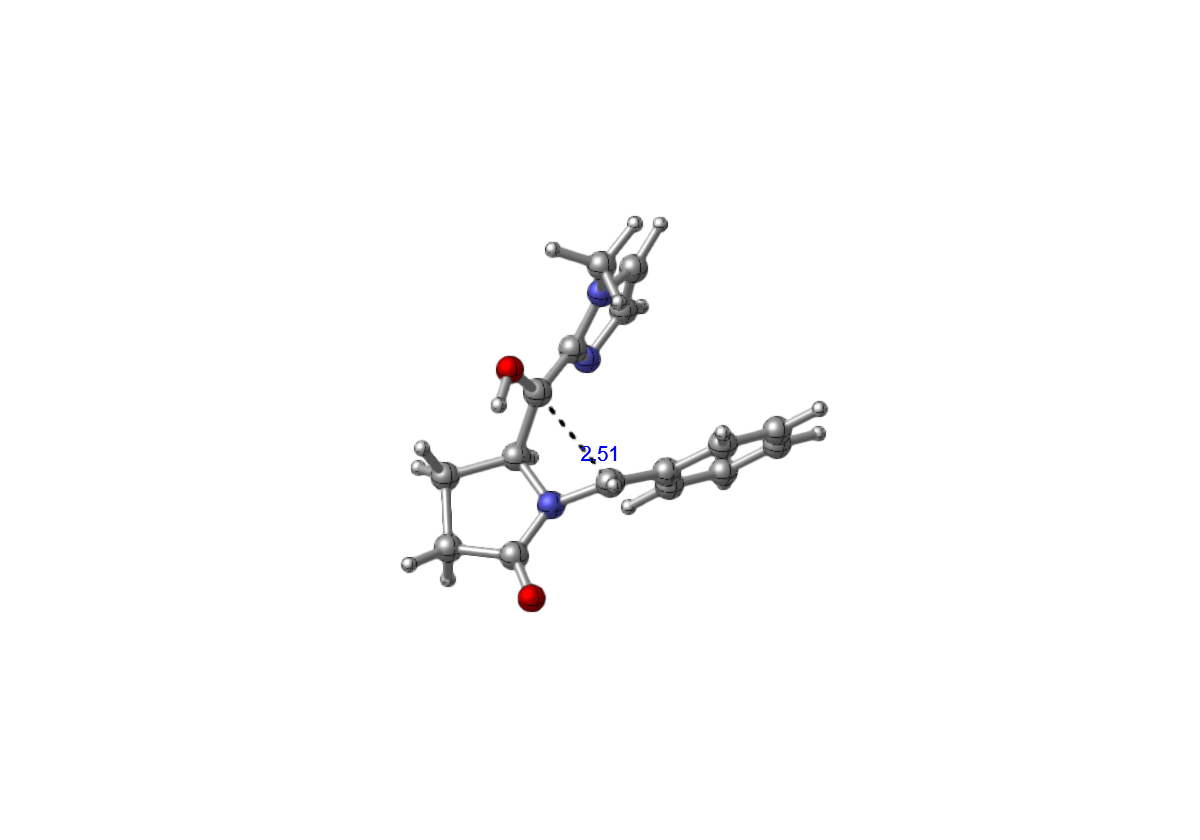


Zero-point correction= 0.305902 (Hartree/Particle)

Thermal correction to Energy= 0.324171

Thermal correction to Enthalpy= 0.325115

Thermal correction to Gibbs Free Energy= 0.257818

Sum of electronic and zero-point Energies= -933.648598

Sum of electronic and thermal Energies= -933.630329

Sum of electronic and thermal Enthalpies= -933.629385

Sum of electronic and thermal Free Energies= -933.696682

UB3LYP-D3/def2TZVPP-CPCM(ACN)

E(scf)= -934.987183183

UM062X-D3/def2TZVPP-CPCM(ACN)

E(scf)= -934.562051688

UTPSSH/def2TZVPP-CPCM(ACN)

E(scf)= -934.992603348

UM06-D3/def2TZVPP-CPCM(ACN)

E(scf)= -934.302921839

E(scf) = -934.486076647a.u.

ν_min_ = 16.9025 cm^-1^

| C | -0.888355 | -3.042566 | -2.742014 |
| --- | --- | --- | --- |
| C | -1.422164 | -1.641451 | -3.014967 |
| N | -1.564078 | -1.007868 | -1.804623 |
| C | -1.099174 | -1.787988 | -0.681670 |
| C | -1.090972 | -3.242647 | -1.236725 |
| O | -1.668806 | -1.145056 | -4.099929 |
| C | -1.911195 | 0.398299 | -1.674569 |
| C | 0.314982 | -1.357112 | -0.276606 |
| C | 0.757449 | -1.631209 | 1.115063 |
| O | 1.077724 | -0.835747 | -1.062800 |
| N | 0.008118 | -2.110463 | 2.129112 |
| C | 0.762104 | -2.224251 | 3.258947 |
| C | 2.025934 | -1.798896 | 2.923605 |
| N | 2.003030 | -1.438100 | 1.601809 |
| C | 3.161018 | -0.927020 | 0.857111 |
| C | -2.981482 | 0.628031 | -0.627804 |
| C | -4.212873 | -0.041626 | -0.720379 |
| C | -5.200476 | 0.150905 | 0.248321 |
| C | -4.969826 | 1.018033 | 1.324470 |
| C | -3.746090 | 1.686007 | 1.425808 |
| C | -2.756063 | 1.487397 | 0.455655 |
| H | -1.407049 | -3.781849 | -3.367298 |
| H | 0.176972 | -3.053009 | -3.025520 |
| H | -1.791156 | -1.681744 | 0.168063 |
| H | -0.324523 | -3.872994 | -0.765228 |
| H | -2.072131 | -3.692149 | -1.028554 |
| H | -2.254748 | 0.715836 | -2.670616 |
| H | -1.009197 | 0.986961 | -1.434796 |
| H | 0.358374 | -2.587799 | 4.199354 |
| H | 2.927220 | -1.728535 | 3.525980 |
| H | 4.023108 | -0.930012 | 1.532290 |
| H | 3.352451 | -1.570669 | -0.009132 |
| H | 2.950685 | 0.090085 | 0.507446 |
| H | -4.391631 | -0.723318 | -1.556626 |
| H | -6.154821 | -0.375368 | 0.164524 |
| H | -5.742425 | 1.168710 | 2.082693 |
| H | -3.556447 | 2.359837 | 2.265223 |
| H | -1.796083 | 2.003975 | 0.543602 |
| H | -0.979883 | -2.346692 | 2.066901 |


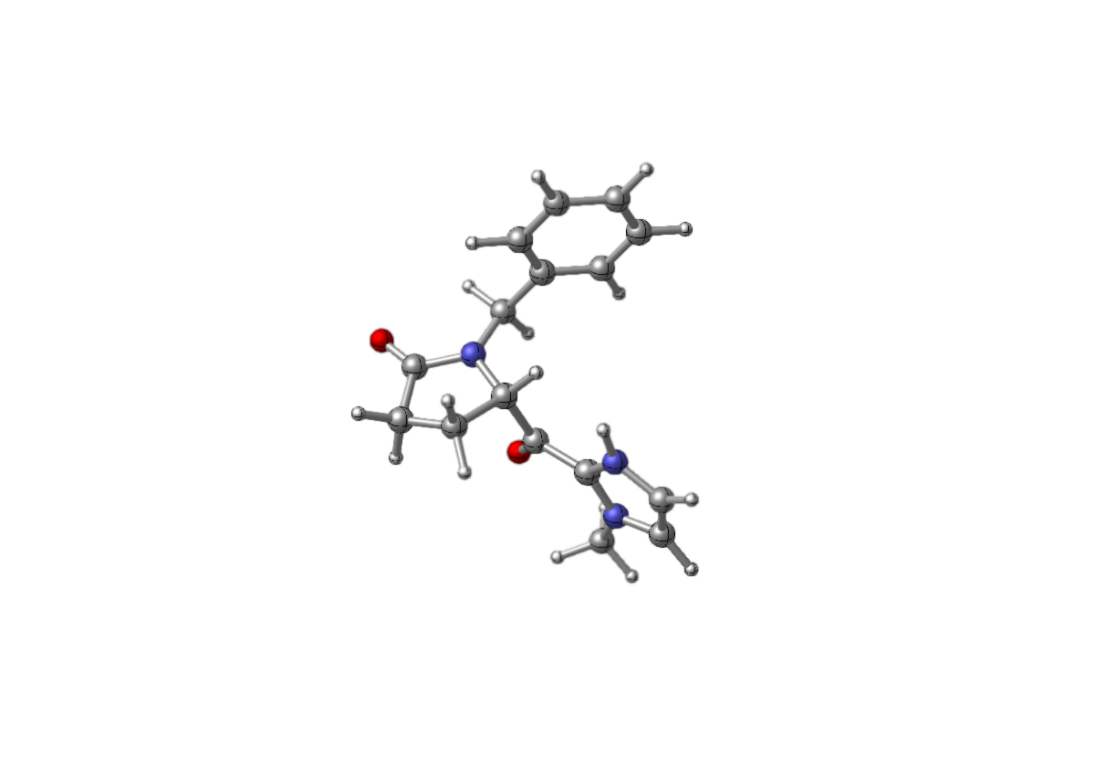


Zero-point correction= 0.323729 (Hartree/Particle)

Thermal correction to Energy= 0.342081

Thermal correction to Enthalpy= 0.343025

Thermal correction to Gibbs Free Energy= 0.273815

Sum of electronic and zero-point Energies= -934.162348

Sum of electronic and thermal Energies= -934.143995

Sum of electronic and thermal Enthalpies= -934.143051

Sum of electronic and thermal Free Energies= -934.212262

UB3LYP-D3/def2TZVPP-CPCM(ACN)

E(scf)= -935.516145791

UM062X-D3/def2TZVPP-CPCM(ACN)/

E(scf)= -935.091432381

UTPSSH/def2TZVPP-CPCM(ACN)

E(scf)= -935.522864696

UM06-D3/def2TZVPP-CPCM(ACN)

E(scf)= -934.826546284

E(scf) = -934.398324717 a.u.

ν_min_ = 25.5289cm^-1^

| C | -0.528247 | -2.999578 | -2.996047 |
| --- | --- | --- | --- |
| C | -1.068726 | -1.588398 | -3.079751 |
| N | -1.229886 | -1.128248 | -1.754518 |
| C | -0.801448 | -2.079660 | -0.795792 |
| C | -0.804983 | -3.418589 | -1.550969 |
| O | -1.323419 | -0.914679 | -4.049636 |
| C | -1.624141 | 0.238409 | -1.452295 |
| C | 0.668820 | -1.713588 | -0.241600 |
| C | 0.790590 | -1.013230 | 0.972974 |
| O | 1.626500 | -2.098743 | -0.966226 |
| N | -0.203181 | -0.555846 | 1.799597 |
| C | 0.350405 | 0.076087 | 2.890917 |
| C | 1.705743 | 0.007191 | 2.749242 |
| N | 1.977704 | -0.658678 | 1.569165 |
| C | 3.306538 | -0.954966 | 1.048240 |
| C | -2.690184 | 0.331531 | -0.379385 |
| C | -3.790514 | -0.541843 | -0.379513 |
| C | -4.774394 | -0.438923 | 0.607258 |
| C | -4.674767 | 0.540898 | 1.602125 |
| C | -3.586640 | 1.418887 | 1.604451 |
| C | -2.598102 | 1.313447 | 0.619261 |
| H | -0.978568 | -3.638149 | -3.767166 |
| H | 0.557661 | -2.942401 | -3.189622 |
| H | -1.441452 | -2.077051 | 0.098117 |
| H | -0.074026 | -4.125618 | -1.143245 |
| H | -1.809058 | -3.858025 | -1.457670 |
| H | -1.973203 | 0.667249 | -2.403998 |
| H | -0.727182 | 0.810387 | -1.155346 |
| H | -0.261374 | 0.522630 | 3.668661 |
| H | 2.499673 | 0.375982 | 3.392133 |
| H | 3.437593 | -0.494138 | 0.059696 |
| H | 4.049679 | -0.546117 | 1.742702 |
| H | 3.446636 | -2.040905 | 0.959973 |
| H | -3.875980 | -1.309589 | -1.153001 |
| H | -5.623437 | -1.126558 | 0.598440 |
| H | -5.443464 | 0.617962 | 2.374665 |
| H | -3.499803 | 2.185605 | 2.377842 |
| H | -1.744594 | 1.996467 | 0.631538 |
| H | -1.198986 | -0.587019 | 1.604535 |


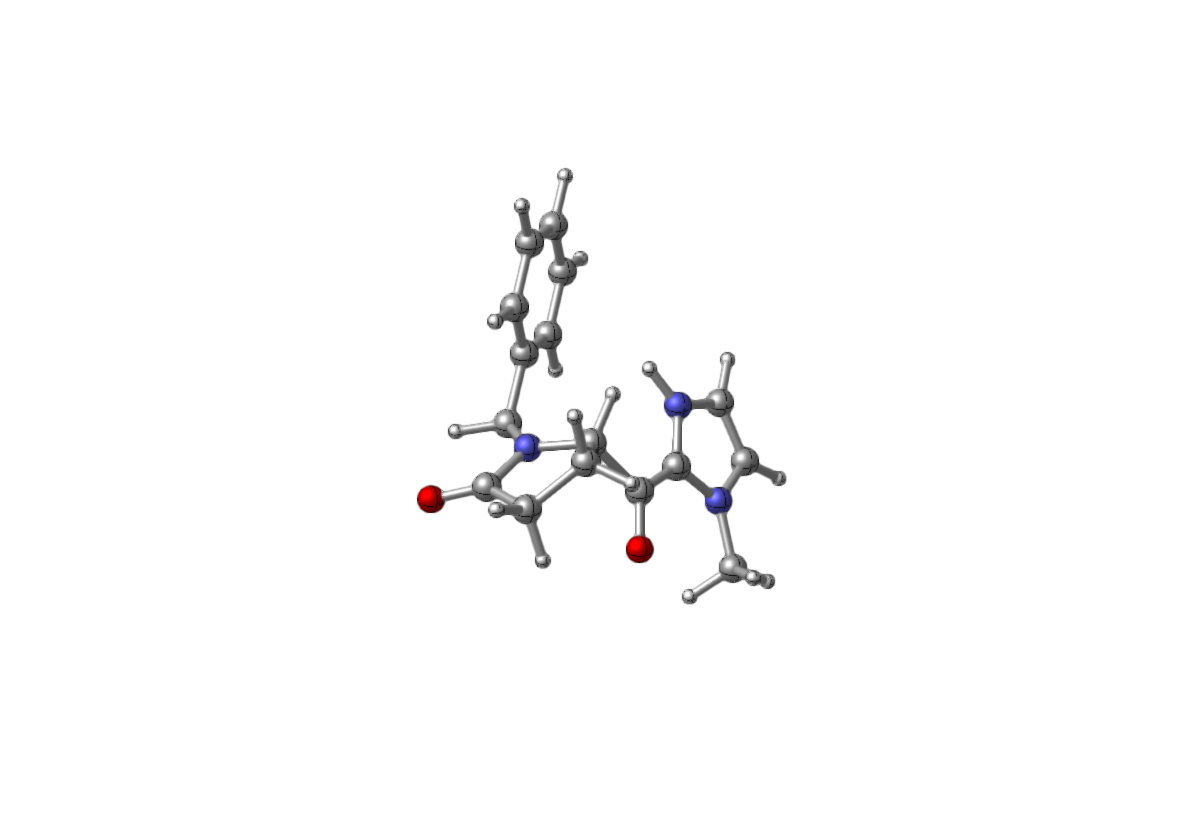


Zero-point correction= 0.319755 (Hartree/Particle)

Thermal correction to Energy= 0.338513

Thermal correction to Enthalpy= 0.339457

Thermal correction to Gibbs Free Energy= 0.269989

Sum of electronic and zero-point Energies= -934.078569

Sum of electronic and thermal Energies= -934.059812

Sum of electronic and thermal Enthalpies= -934.058868

Sum of electronic and thermal Free Energies= -934.128336

UB3LYP-D3/def2TZVPP-CPCM(ACN)

E(scf)= -935.4263748

UM062X-D3/def2TZVPP-CPCM(ACN)

E(scf)= -934.988375357

UTPSSH/def2TZVPP-CPCM(ACN)

E(scf)= -935.435143208

UM06-D3/def2TZVPP-CPCM(ACN)

E(scf)= -934.731360349

**2aH^+^**

E(scf) = -934.494545374 a.u.

ν_min_ = 22.2739cm^-1^

| C | -1.046414 | -3.521902 | -3.211446 |
| --- | --- | --- | --- |
| C | -2.383825 | -2.803903 | -3.140457 |
| N | -2.521308 | -1.825145 | -2.195590 |
| C | -0.318024 | -2.201054 | -1.139450 |
| C | 0.113643 | -2.858334 | -2.461815 |
| O | -3.321378 | -3.128264 | -3.858187 |
| C | -1.463294 | -1.180019 | -1.431563 |
| C | -0.749469 | -3.215652 | -0.091132 |
| C | -0.450817 | -2.931031 | 1.342462 |
| O | -1.362815 | -4.229063 | -0.362534 |
| N | 0.232611 | -1.885137 | 1.848663 |
| C | 0.260735 | -1.950430 | 3.210218 |
| C | -0.429444 | -3.087456 | 3.554366 |
| N | -0.858130 | -3.678246 | 2.393160 |
| C | -1.658335 | -4.908000 | 2.340290 |
| C | -2.001009 | -0.544089 | -0.160367 |
| C | -3.011020 | -1.162941 | 0.593555 |
| C | -3.400623 | -0.630951 | 1.826900 |
| C | -2.789621 | 0.528147 | 2.317666 |
| C | -1.795433 | 1.160048 | 1.563034 |
| C | -1.406010 | 0.627809 | 0.329512 |
| H | -1.241133 | -4.528733 | -2.813358 |
| H | -0.807653 | -3.646446 | -4.278022 |
| H | 0.529310 | -1.615285 | -0.756512 |
| H | 0.550402 | -2.059647 | -3.081777 |
| H | 0.915006 | -3.588796 | -2.276510 |
| H | -0.990932 | -0.384681 | -2.035693 |
| H | -3.423178 | -1.354817 | -2.209932 |
| H | 0.754964 | -1.202538 | 3.822951 |
| H | -0.644133 | -3.511646 | 4.530912 |
| H | -1.817950 | -5.248217 | 3.369044 |
| H | -2.618645 | -4.697872 | 1.855026 |
| H | -1.124930 | -5.670831 | 1.762503 |
| H | -3.485585 | -2.073108 | 0.219703 |
| H | -4.182076 | -1.127288 | 2.407124 |
| H | -3.091695 | 0.940918 | 3.283051 |
| H | -1.321231 | 2.071669 | 1.934185 |
| H | -0.626544 | 1.124970 | -0.254854 |
| H | 0.643994 | -1.136263 | 1.297201 |


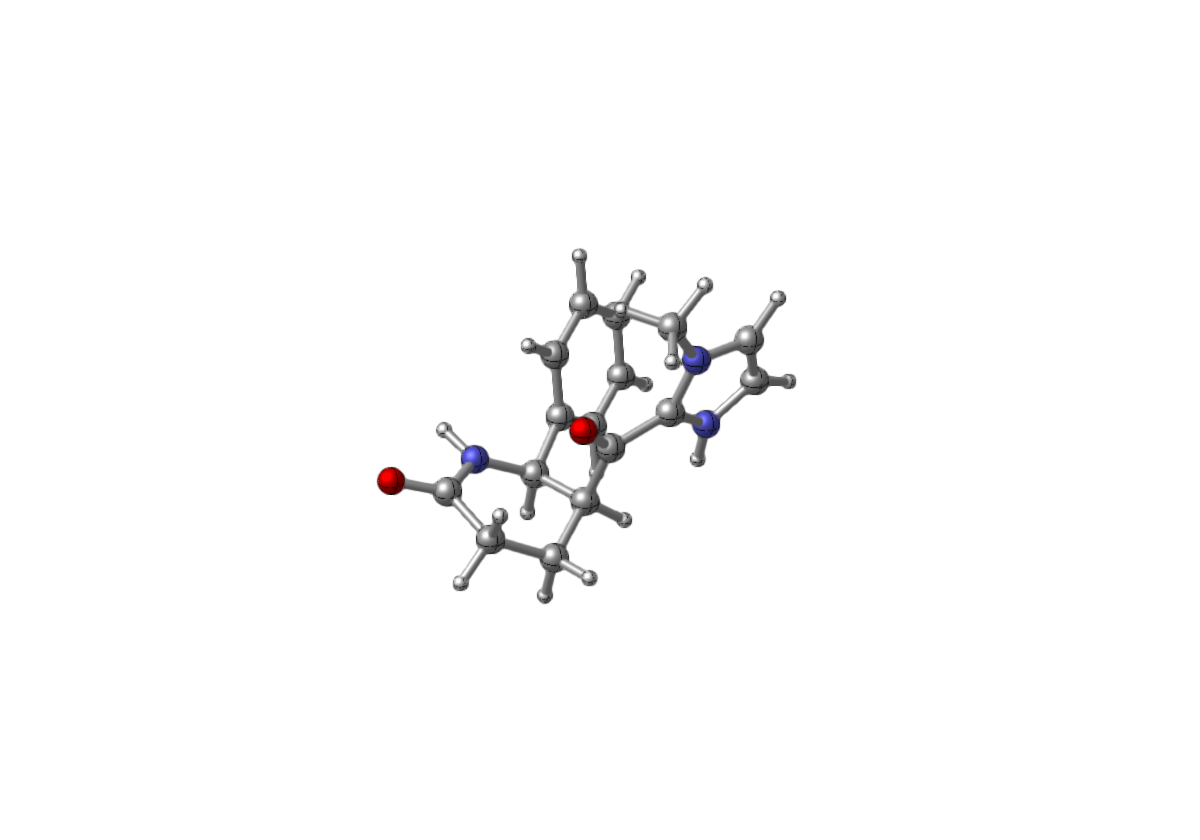


Zero-point correction= 0.324950 (Hartree/Particle)

Thermal correction to Energy= 0.342841

Thermal correction to Enthalpy= 0.343785

Thermal correction to Gibbs Free Energy= 0.277908

Sum of electronic and zero-point Energies= -934.169595

Sum of electronic and thermal Energies= -934.151704

Sum of electronic and thermal Enthalpies= -934.150760

Sum of electronic and thermal Free Energies= -934.216637

UB3LYP-D3/def2TZVPP-CPCM(ACN)

E(scf)= -935.523412503

UM062X-D3/def2TZVPP-CPCM(ACN)

E(scf)= -935.100504164

UTPSSH/def2TZVPP-CPCM(ACN)

E(scf)= -935.521371051

UM06-D3/def2TZVPP-CPCM(ACN)

E(scf)= -934.835311669

E(scf) = -934.425212632 a.u.

ν_min_ = 26.4431cm^-1^

| C | -0.410235 | -3.394778 | -3.795381 |
| --- | --- | --- | --- |
| C | -1.169541 | -2.099825 | -3.995068 |
| N | -1.539833 | -1.614030 | -2.729207 |
| C | -1.042466 | -2.500254 | -1.632993 |
| C | 0.068561 | -3.312287 | -2.348734 |
| O | -1.447415 | -1.541419 | -5.034764 |
| C | -2.079951 | -0.349005 | -2.597012 |
| C | -0.566733 | -1.708396 | -0.459371 |
| C | -0.846598 | -2.041884 | 0.881616 |
| O | 0.177371 | -0.611339 | -0.679074 |
| N | -1.340052 | -3.231842 | 1.332316 |
| C | -1.509841 | -3.187035 | 2.696692 |
| C | -1.111414 | -1.945676 | 3.101960 |
| N | -0.702743 | -1.243892 | 1.983833 |
| C | -0.330871 | 0.170839 | 1.982642 |
| C | -2.782881 | 0.139877 | -1.462828 |
| C | -3.329108 | -0.699700 | -0.449431 |
| C | -3.915930 | -0.151831 | 0.692618 |
| C | -3.993629 | 1.234715 | 0.856022 |
| C | -3.501728 | 2.082515 | -0.155824 |
| C | -2.915785 | 1.551843 | -1.295033 |
| H | -1.111078 | -4.232185 | -3.956133 |
| H | 0.394466 | -3.483654 | -4.537144 |
| H | -1.855454 | -3.176175 | -1.327834 |
| H | 1.012661 | -2.750287 | -2.289506 |
| H | 0.213227 | -4.286244 | -1.864444 |
| H | -1.915858 | 0.290131 | -3.467231 |
| H | -0.035353 | -0.209784 | -1.542456 |
| H | -1.887317 | -4.033123 | 3.262465 |
| H | -1.091361 | -1.502325 | 4.093100 |
| H | -0.451047 | 0.554186 | 3.002052 |
| H | 0.708438 | 0.293850 | 1.656550 |
| H | -0.991814 | 0.724400 | 1.302676 |
| H | -3.356735 | -1.779028 | -0.588377 |
| H | -4.324937 | -0.817635 | 1.456333 |
| H | -4.446776 | 1.658718 | 1.754857 |
| H | -3.576725 | 3.166589 | -0.041216 |
| H | -2.520351 | 2.216030 | -2.067796 |
| H | -1.502738 | -4.052890 | 0.758763 |


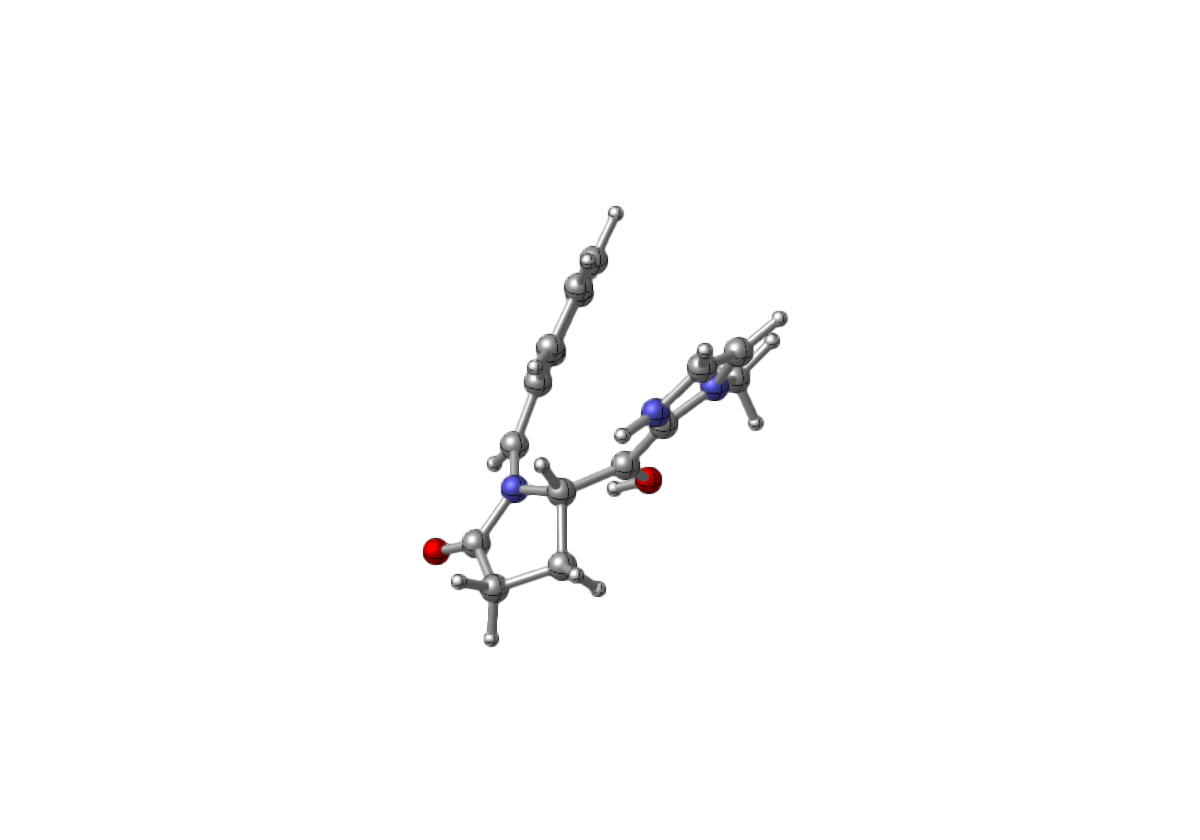


Zero-point correction= 0.320241 (Hartree/Particle)

Thermal correction to Energy= 0.338894

Thermal correction to Enthalpy= 0.339839

Thermal correction to Gibbs Free Energy= 0.273114

Sum of electronic and zero-point Energies= -934.104972

Sum of electronic and thermal Energies= -934.086318

Sum of electronic and thermal Enthalpies= -934.085374

Sum of electronic and thermal Free Energies= -934.152099

UB3LYP-D3/def2TZVPP-CPCM(ACN)

E(scf)= -935.453582254

UM062X-D3/ def2TZVPP-CPCM(ACN)

E(scf)= -935.024220792

UTPSSH/ def2TZVPP-CPCM(ACN)

E(scf)= -935.457445623

UM06-D3/def2TZVPP-CPCM(ACN)

E(scf)= -934.765304243

E(scf) = -934.422310130a.u.

ν_min_ = 23.7622cm^-1^

| C | -1.145406 | -3.309590 | -3.481202 |
| --- | --- | --- | --- |
| C | -1.792673 | -1.939831 | -3.428922 |
| N | -1.669903 | -1.455086 | -2.121563 |
| C | -0.928089 | -2.392131 | -1.243750 |
| C | -0.229676 | -3.335070 | -2.257974 |
| O | -2.345833 | -1.334312 | -4.324310 |
| C | -2.109643 | -0.180284 | -1.785412 |
| C | 0.021048 | -1.638536 | -0.354916 |
| C | 0.067349 | -1.789282 | 1.045345 |
| O | 0.839766 | -0.723161 | -0.895092 |
| N | -0.451931 | -2.828742 | 1.760341 |
| C | -0.284483 | -2.607628 | 3.104021 |
| C | 0.353079 | -1.403819 | 3.228096 |
| N | 0.570950 | -0.906383 | 1.961298 |
| C | 1.090080 | 0.432031 | 1.675280 |
| C | -2.392320 | 0.304022 | -0.474552 |
| C | -2.729114 | -0.525749 | 0.634339 |
| C | -2.930604 | 0.015589 | 1.900312 |
| C | -2.826624 | 1.398704 | 2.111948 |
| C | -2.546775 | 2.240542 | 1.023364 |
| C | -2.341891 | 1.710864 | -0.245968 |
| H | -1.946912 | -4.065206 | -3.411800 |
| H | -0.630663 | -3.455772 | -4.440362 |
| H | -1.638331 | -2.963968 | -0.628002 |
| H | 0.758039 | -2.922796 | -2.514293 |
| H | -0.080067 | -4.332865 | -1.826469 |
| H | -2.192247 | 0.485821 | -2.646595 |
| H | 0.554106 | -0.486047 | -1.792880 |
| H | -0.618498 | -3.315714 | 3.856110 |
| H | 0.659189 | -0.859670 | 4.116693 |
| H | 1.290096 | 0.929230 | 2.630704 |
| H | 2.011772 | 0.366134 | 1.086381 |
| H | 0.337985 | 1.003185 | 1.114278 |
| H | -2.860179 | -1.597970 | 0.491568 |
| H | -3.180948 | -0.647036 | 2.732528 |
| H | -2.977433 | 1.817111 | 3.109489 |
| H | -2.481651 | 3.321261 | 1.172792 |
| H | -2.108002 | 2.374669 | -1.082245 |
| H | -0.840939 | -3.675978 | 1.358537 |


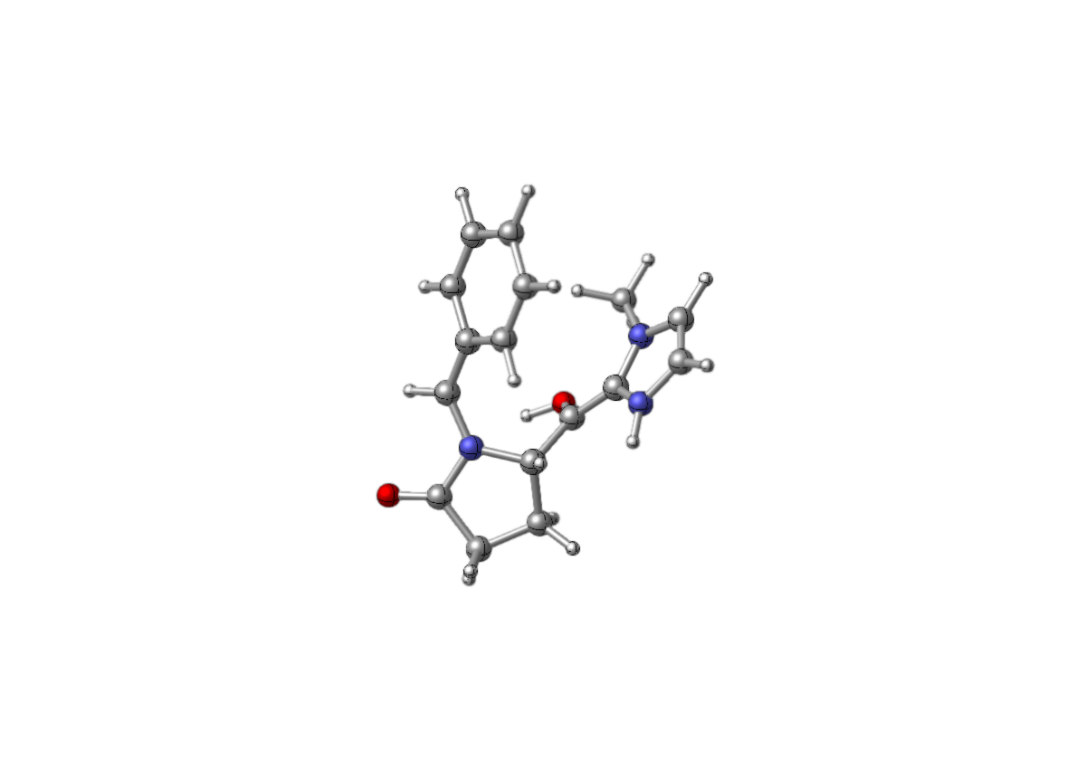


Zero-point correction= 0.320582 (Hartree/Particle)

Thermal correction to Energy= 0.339309

Thermal correction to Enthalpy= 0.340253

Thermal correction to Gibbs Free Energy= 0.271910

Sum of electronic and zero-point Energies= -934.101728

Sum of electronic and thermal Energies= -934.083001

Sum of electronic and thermal Enthalpies= -934.082057

Sum of electronic and thermal Free Energies= -934.150400

UB3LYP-D3/def2TZVPP-CPCM(ACN)

E(scf)= -935.450971223

UM062X-D3/def2TZVPP-CPCM(ACN)

E(scf)= -935.02268836

UTPSSH/def2TZVPP-CPCM(ACN)

E(scf)= -935.453462197

UM06-D3/def2TZVPP-CPCM(ACN)

E(scf)= -934.762931717

E(scf) = -934.458583284 a.u.

ν_min_ = 15.7565cm^-1^

| C | -1.001148 | -3.386567 | -3.103049 |
| --- | --- | --- | --- |
| C | -1.769000 | -2.081361 | -3.097900 |
| N | -2.105120 | -1.631553 | -1.788121 |
| C | 0.564142 | -3.399934 | -0.956877 |
| C | 0.396099 | -3.303250 | -2.448623 |
| O | -2.077561 | -1.482350 | -4.105871 |
| C | -2.420548 | -0.388356 | -1.630578 |
| C | -0.346904 | -3.543098 | 0.032725 |
| C | 0.097186 | -3.669920 | 1.431326 |
| O | -1.692137 | -3.574635 | -0.099213 |
| N | 1.262310 | -3.229747 | 1.944607 |
| C | 1.337127 | -3.522043 | 3.286672 |
| C | 0.173877 | -4.160265 | 3.603127 |
| N | -0.582398 | -4.244965 | 2.446199 |
| C | -1.900146 | -4.886343 | 2.379470 |
| C | -2.790945 | 0.218608 | -0.362784 |
| C | -2.818191 | -0.506920 | 0.850320 |
| C | -3.178562 | 0.130284 | 2.033488 |
| C | -3.516391 | 1.492541 | 2.026932 |
| C | -3.491293 | 2.221234 | 0.832428 |
| C | -3.128081 | 1.588895 | -0.356457 |
| H | -1.607658 | -4.161931 | -2.608663 |
| H | -0.878994 | -3.672248 | -4.155868 |
| H | 1.615322 | -3.401582 | -0.654486 |
| H | 0.907846 | -2.386957 | -2.797282 |
| H | 0.999607 | -4.124317 | -2.875509 |
| H | -2.406797 | 0.273611 | -2.512016 |
| H | -1.962863 | -2.823534 | -0.738743 |
| H | 2.193482 | -3.249346 | 3.895594 |
| H | -0.178801 | -4.562910 | 4.547870 |
| H | -2.017002 | -5.511304 | 3.271998 |
| H | -2.686718 | -4.122770 | 2.347431 |
| H | -1.966621 | -5.505462 | 1.479498 |
| H | -2.551872 | -1.564001 | 0.858696 |
| H | -3.196582 | -0.432409 | 2.969536 |
| H | -3.798550 | 1.986695 | 2.959878 |
| H | -3.753234 | 3.281415 | 0.830025 |
| H | -3.104378 | 2.153420 | -1.292102 |
| H | 1.959509 | -2.710849 | 1.418651 |


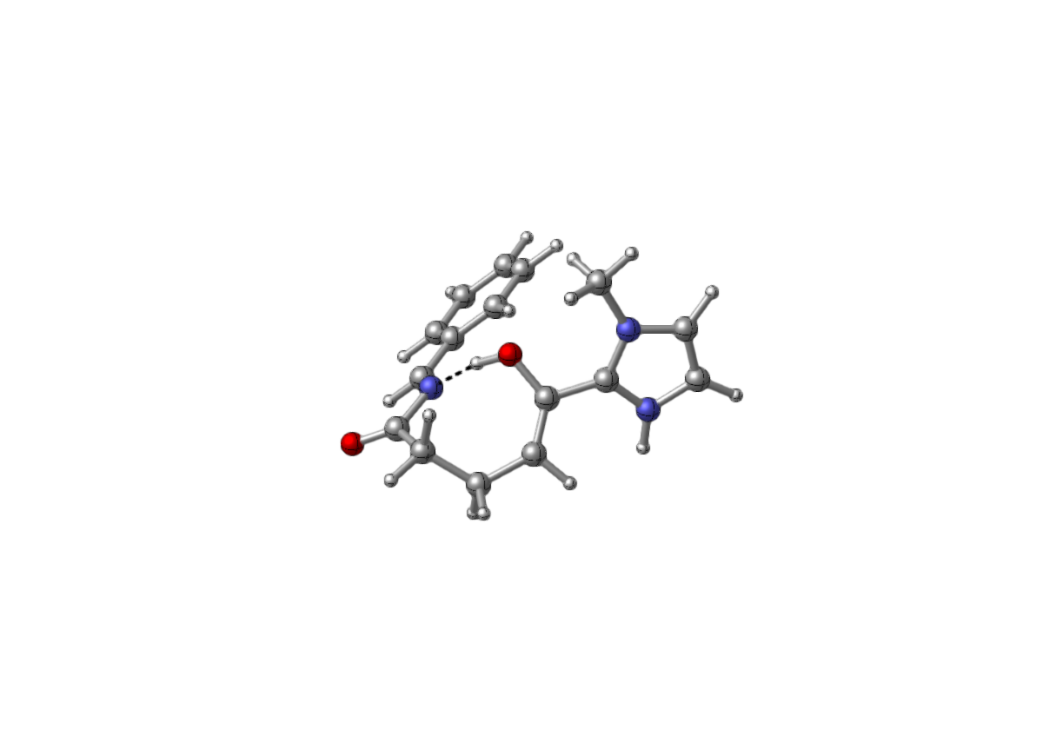


Zero-point correction= 0.321045 (Hartree/Particle)

Thermal correction to Energy= 0.339972

Thermal correction to Enthalpy= 0.340916

Thermal correction to Gibbs Free Energy= 0.271095

Sum of electronic and zero-point Energies= -934.137539

Sum of electronic and thermal Energies= -934.118612

Sum of electronic and thermal Enthalpies= -934.117668

Sum of electronic and thermal Free Energies= -934.187488

UB3LYP-D3/def2TZVPP-CPCM(ACN)

E(scf)= -935.48921791

UM062X-D3/def2TZVPP-CPCM(ACN)

E(scf)= -935.059478643

UTPSSH/def2TZVPP-CPCM(ACN)

E(scf)= -935.491025038

UM06-D3/def2TZVPP-CPCM(ACN)

E(scf)= -934.79195757

E(scf) = -934.454813757a.u.

ν_min_ = 30.3226cm^-1^

| C | -1.216791 | -3.289347 | -3.220192 |
| --- | --- | --- | --- |
| C | -2.024525 | -2.001918 | -3.199958 |
| N | -2.221639 | -1.472551 | -1.922140 |
| C | -0.027299 | -3.174565 | -0.954561 |
| C | 0.118698 | -3.234000 | -2.446431 |
| O | -2.524658 | -1.519896 | -4.198960 |
| C | -1.990134 | -0.238492 | -1.672935 |
| C | 0.373851 | -2.155710 | -0.165700 |
| C | 0.193106 | -2.170863 | 1.287499 |
| O | 0.931110 | -0.995806 | -0.597578 |
| N | -0.222043 | -3.229980 | 2.011254 |
| C | -0.317499 | -2.895741 | 3.340043 |
| C | 0.060737 | -1.588387 | 3.432800 |
| N | 0.379006 | -1.153562 | 2.158524 |
| C | 0.809102 | 0.219336 | 1.865983 |
| C | -2.228299 | 0.360117 | -0.355977 |
| C | -2.641817 | -0.428569 | 0.736768 |
| C | -2.833898 | 0.153920 | 1.987760 |
| C | -2.623903 | 1.529855 | 2.162181 |
| C | -2.220448 | 2.321971 | 1.081406 |
| C | -2.017121 | 1.739238 | -0.171753 |
| H | -1.854713 | -4.083331 | -2.793200 |
| H | -1.034038 | -3.542208 | -4.273660 |
| H | -0.521696 | -4.032449 | -0.494618 |
| H | 0.730777 | -2.402864 | -2.835010 |
| H | 0.679975 | -4.150281 | -2.697373 |
| H | -1.573429 | 0.429191 | -2.449200 |
| H | 1.009726 | -0.990675 | -1.562014 |
| H | -0.635490 | -3.605750 | 4.097072 |
| H | 0.130151 | -0.930777 | 4.293757 |
| H | 0.779733 | 0.783272 | 2.804012 |
| H | 1.826257 | 0.213587 | 1.460211 |
| H | 0.129186 | 0.673640 | 1.139103 |
| H | -2.794721 | -1.498820 | 0.586374 |
| H | -3.141334 | -0.463702 | 2.834585 |
| H | -2.771402 | 1.982931 | 3.145415 |
| H | -2.055289 | 3.392908 | 1.218294 |
| H | -1.690165 | 2.353079 | -1.015156 |
| H | -0.417922 | -4.150895 | 1.630237 |


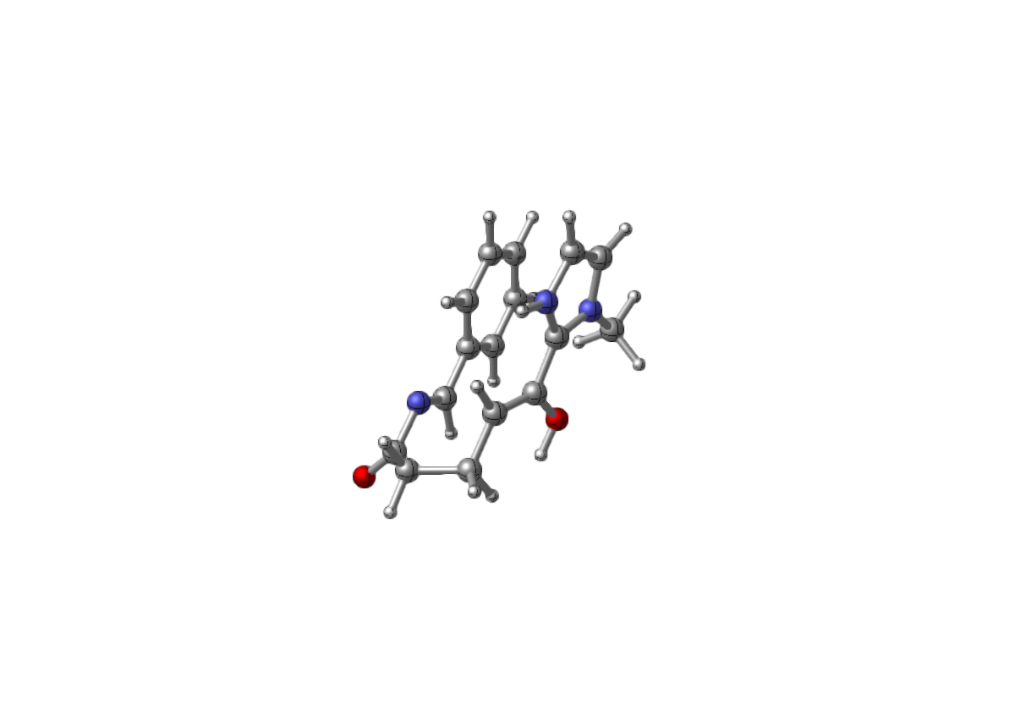


Zero-point correction= 0.321305 (Hartree/Particle)

Thermal correction to Energy= 0.340421

Thermal correction to Enthalpy= 0.341365

Thermal correction to Gibbs Free Energy= 0.273214

Sum of electronic and zero-point Energies= -934.133508

Sum of electronic and thermal Energies= -934.114393

Sum of electronic and thermal Enthalpies= -934.113449

Sum of electronic and thermal Free Energies= -934.181600

**C’’H^+^**

E(scf) = -934.450524157a.u.

ν_min_ = 13.4037cm^-1^

| C | -0.237479 | -3.221815 | -2.956611 |
| --- | --- | --- | --- |
| C | -1.503595 | -2.406171 | -3.154764 |
| N | -1.991045 | -1.834512 | -1.975559 |
| C | 0.743535 | -2.902689 | -0.533882 |
| C | 0.812513 | -2.613523 | -2.008857 |
| O | -2.116698 | -2.367254 | -4.202200 |
| C | -2.165129 | -0.613057 | -1.628107 |
| C | -0.254089 | -3.421022 | 0.219360 |
| C | -0.015984 | -3.708271 | 1.641484 |
| O | -1.513244 | -3.736198 | -0.159646 |
| N | 1.130400 | -4.161253 | 2.180574 |
| C | 0.992756 | -4.308278 | 3.541064 |
| C | -0.288435 | -3.939579 | 3.837170 |
| N | -0.899376 | -3.572601 | 2.651600 |
| C | -2.272405 | -3.070758 | 2.541130 |
| C | -1.788013 | 0.634192 | -2.303178 |
| C | -1.114249 | 0.697584 | -3.542133 |
| C | -0.778005 | 1.932092 | -4.092950 |
| C | -1.109487 | 3.119484 | -3.425339 |
| C | -1.778875 | 3.070534 | -2.198247 |
| C | -2.113134 | 1.836353 | -1.640579 |
| H | -0.545612 | -4.221561 | -2.605264 |
| H | 0.191999 | -3.359787 | -3.958393 |
| H | 1.658099 | -2.639359 | 0.005473 |
| H | 0.860823 | -1.517670 | -2.156873 |
| H | 1.801551 | -2.971092 | -2.341413 |
| H | -2.644654 | -0.470473 | -0.647967 |
| H | -1.844144 | -3.061611 | -0.820953 |
| H | 1.798820 | -4.666155 | 4.174005 |
| H | -0.813522 | -3.905524 | 4.787149 |
| H | -2.280557 | -2.146909 | 1.950454 |
| H | -2.904573 | -3.818685 | 2.049358 |
| H | -2.642151 | -2.864473 | 3.551709 |
| H | -0.860610 | -0.210243 | -4.086710 |
| H | -0.256515 | 1.971860 | -5.051798 |
| H | -0.844159 | 4.083868 | -3.865246 |
| H | -2.037314 | 3.993685 | -1.674998 |
| H | -2.630315 | 1.796861 | -0.678423 |
| H | 1.957224 | -4.397972 | 1.638621 |


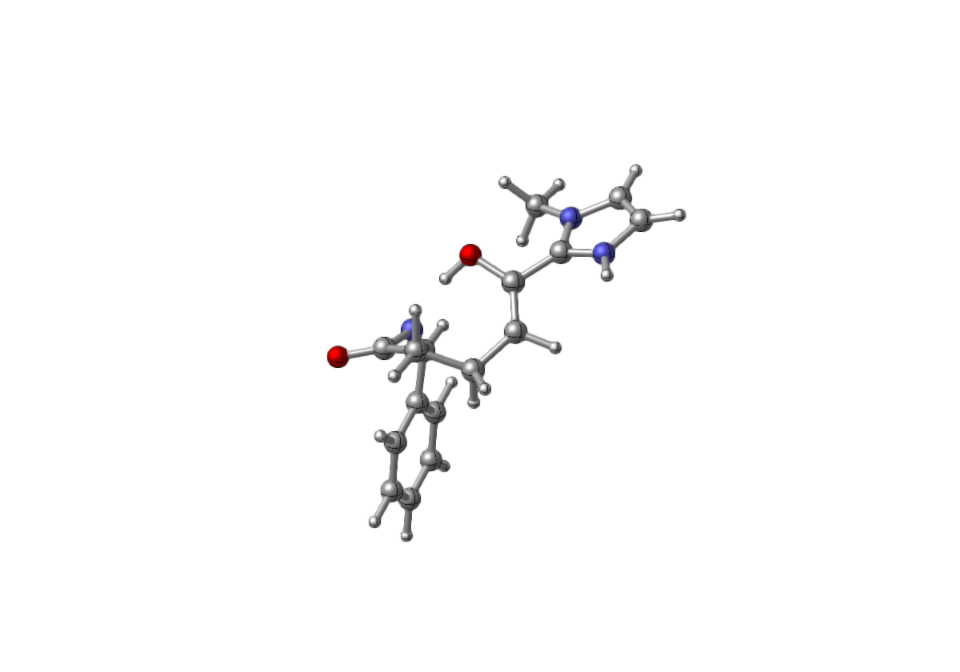


Zero-point correction= 0.321134 (Hartree/Particle)

Thermal correction to Energy= 0.340154

Thermal correction to Enthalpy= 0.341098

Thermal correction to Gibbs Free Energy= 0.270760

Sum of electronic and zero-point Energies= -934.129390

Sum of electronic and thermal Energies= -934.110370

Sum of electronic and thermal Enthalpies= -934.109426

Sum of electronic and thermal Free Energies= -934.179764

**DH^+^**

E(scf) = -934.451950276a.u.

ν_min_ = 33.3587cm^-1^

| C | -1.339237 | -3.237656 | -3.472956 |
| --- | --- | --- | --- |
| C | -2.012009 | -1.908358 | -3.111775 |
| N | -1.863709 | -1.760060 | -1.743626 |
| C | -0.842666 | -2.657960 | -1.194260 |
| C | -0.236677 | -3.392048 | -2.402362 |
| O | -2.523718 | -1.095443 | -3.852929 |
| C | -1.373676 | -0.549535 | -1.067514 |
| C | -0.113462 | -1.411179 | -0.560371 |
| C | 0.080492 | -1.421330 | 0.926395 |
| O | 1.112429 | -1.068901 | -1.130036 |
| N | -0.523745 | -2.239802 | 1.801438 |
| C | -0.185602 | -1.898350 | 3.089505 |
| C | 0.657811 | -0.830309 | 2.989525 |
| N | 0.807921 | -0.544906 | 1.644383 |
| C | 1.655504 | 0.539791 | 1.134574 |
| C | -2.265232 | 0.045362 | -0.009102 |
| C | -3.355982 | -0.666825 | 0.508422 |
| C | -4.119223 | -0.130068 | 1.550099 |
| C | -3.797815 | 1.121661 | 2.084862 |
| C | -2.715430 | 1.841832 | 1.565720 |
| C | -1.956488 | 1.308216 | 0.521361 |
| H | -2.084258 | -4.048715 | -3.410205 |
| H | -0.969744 | -3.197501 | -4.505933 |
| H | -1.253917 | -3.367131 | -0.458967 |
| H | 0.686619 | -2.887067 | -2.722364 |
| H | 0.012937 | -4.436369 | -2.176197 |
| H | -1.049924 | 0.227930 | -1.779858 |
| H | 0.984293 | -0.644517 | -1.990356 |
| H | -0.564688 | -2.436252 | 3.952755 |
| H | 1.163037 | -0.251944 | 3.757203 |
| H | 1.850117 | 1.231966 | 1.961135 |
| H | 2.596310 | 0.127004 | 0.752381 |
| H | 1.141924 | 1.068461 | 0.326655 |
| H | -3.607770 | -1.641518 | 0.084461 |
| H | -4.968845 | -0.692844 | 1.944406 |
| H | -4.393533 | 1.539515 | 2.900042 |
| H | -2.465553 | 2.825500 | 1.970428 |
| H | -1.122818 | 1.883882 | 0.110295 |
| H | -1.145427 | -3.003068 | 1.550888 |


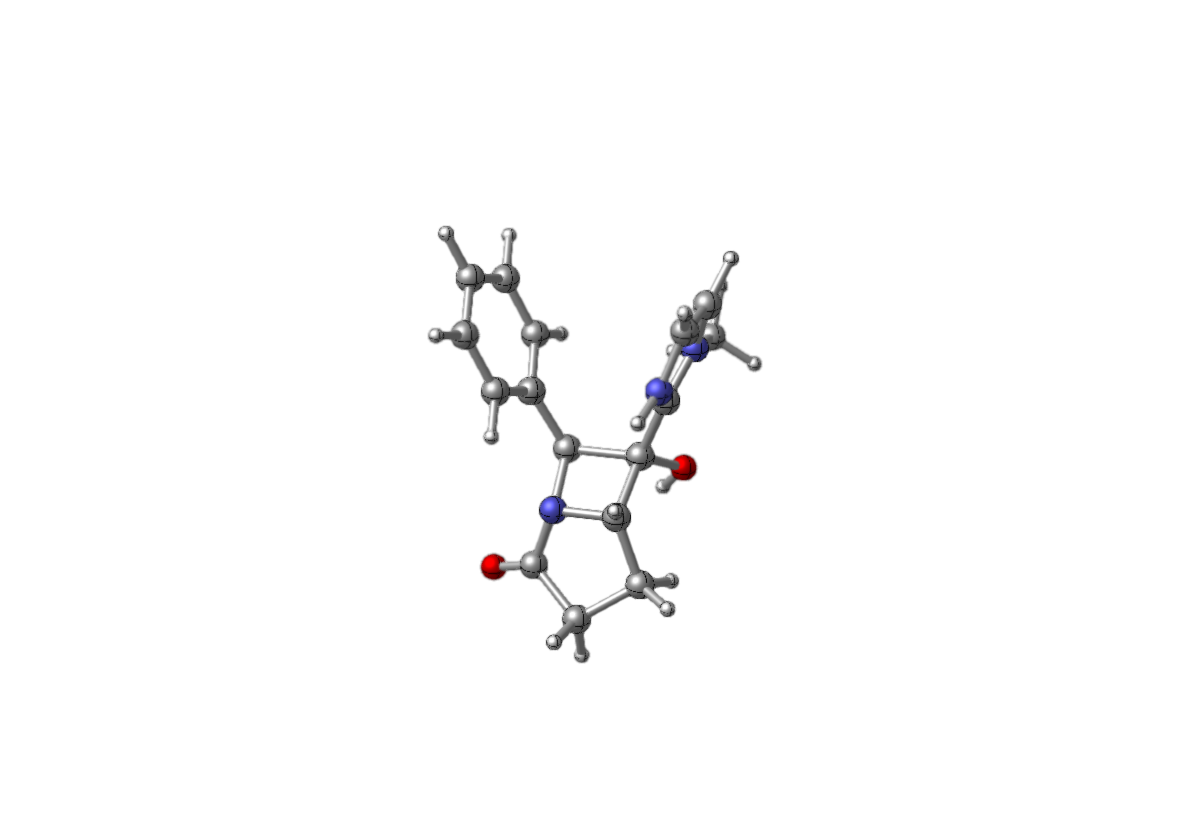


Zero-point correction= 0.324175 (Hartree/Particle)

Thermal correction to Energy= 0.341887

Thermal correction to Enthalpy= 0.342831

Thermal correction to Gibbs Free Energy= 0.278087

Sum of electronic and zero-point Energies= -934.127776

Sum of electronic and thermal Energies= -934.110064

Sum of electronic and thermal Enthalpies= -934.109119

Sum of electronic and thermal Free Energies= -934.173863

UB3LYP-D3/def2TZVPP-CPCM(ACN)

E(scf)= -935.480449236

UM062X-D3/def2TZVPP-CPCM(ACN)

E(scf)= -935.063520212

UTPSSH/def2TZVPP-CPCM(ACN)

E(scf)= -935.485384561

UM06-D3/def2TZVPP-CPCM(ACN)

E(scf)= -934.796461918

**EH^+^**

E(scf) = -934.491427674 a.u.

ν_min_ = 27.7992cm^-1^

| C | -0.514772 | -3.432271 | -2.385590 |
| --- | --- | --- | --- |
| C | -1.770238 | -2.931574 | -3.083798 |
| N | -2.304122 | -1.764397 | -2.629678 |
| C | -0.772705 | -1.580077 | -0.692276 |
| C | 0.223100 | -2.373383 | -1.568998 |
| O | -2.291231 | -3.565627 | -3.994130 |
| C | -1.818619 | -0.871454 | -1.585541 |
| C | -1.438928 | -2.495185 | 0.321908 |
| C | -0.610785 | -2.950526 | 1.476128 |
| O | -2.581472 | -2.892582 | 0.224158 |
| N | 0.608978 | -2.498443 | 1.830600 |
| C | 1.045344 | -3.144764 | 2.949946 |
| C | 0.053866 | -4.032543 | 3.292064 |
| N | -0.956911 | -3.897558 | 2.374617 |
| C | -2.203411 | -4.674234 | 2.390748 |
| C | -1.254399 | 0.434328 | -2.142227 |
| C | -0.715752 | 0.518127 | -3.433094 |
| C | -0.164974 | 1.718953 | -3.896166 |
| C | -0.149896 | 2.849480 | -3.074075 |
| C | -0.692330 | 2.775428 | -1.785195 |
| C | -1.242309 | 1.576578 | -1.325248 |
| H | -0.831208 | -4.275913 | -1.747611 |
| H | 0.132586 | -3.865644 | -3.161166 |
| H | -0.227710 | -0.795413 | -0.143916 |
| H | 0.723571 | -1.651935 | -2.232980 |
| H | 1.012211 | -2.838648 | -0.960181 |
| H | -2.678451 | -0.619539 | -0.944949 |
| H | -3.138131 | -1.463784 | -3.127570 |
| H | 2.004235 | -2.933153 | 3.413792 |
| H | -0.002571 | -4.742101 | 4.112728 |
| H | -3.050870 | -4.002323 | 2.568769 |
| H | -2.337789 | -5.172019 | 1.423788 |
| H | -2.127022 | -5.416196 | 3.192610 |
| H | -0.734555 | -0.356821 | -4.085925 |
| H | 0.251256 | 1.769182 | -4.905460 |
| H | 0.277832 | 3.787485 | -3.436690 |
| H | -0.692577 | 3.656574 | -1.138651 |
| H | -1.669569 | 1.526589 | -0.319164 |
| H | 1.124683 | -1.766597 | 1.347484 |


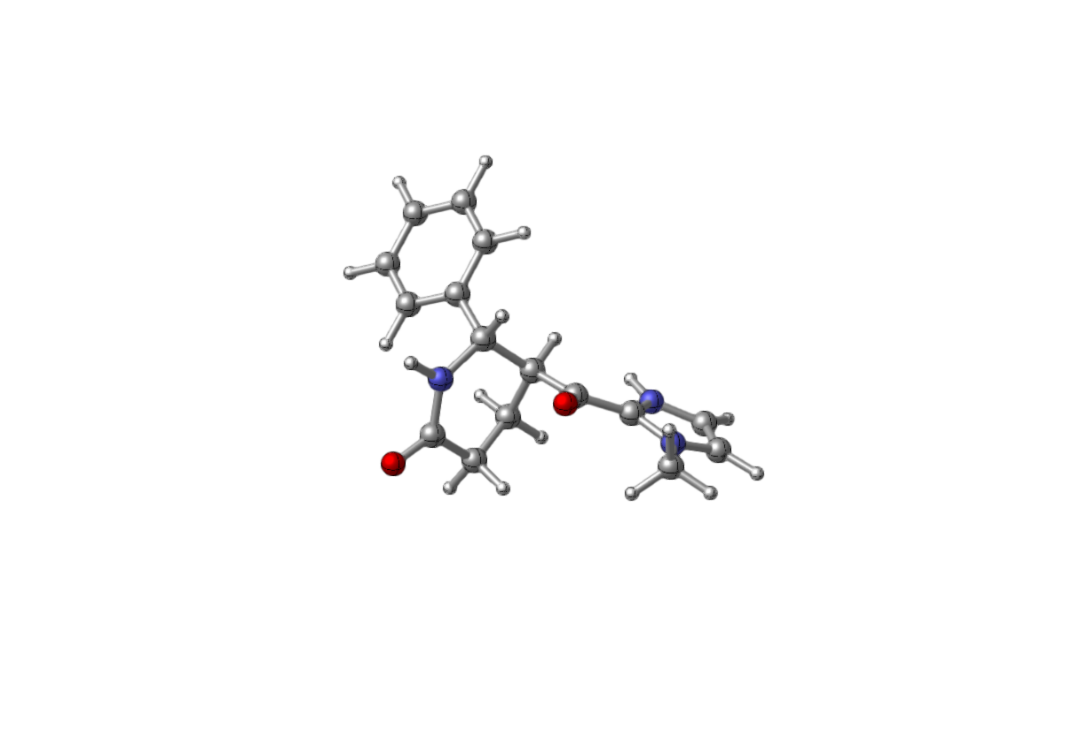


Zero-point correction= 0.324822 (Hartree/Particle)

Thermal correction to Energy= 0.342802

Thermal correction to Enthalpy= 0.343747

Thermal correction to Gibbs Free Energy= 0.277106

Sum of electronic and zero-point Energies= -934.166605

Sum of electronic and thermal Energies= -934.148625

Sum of electronic and thermal Enthalpies= -934.147681

Sum of electronic and thermal Free Energies= -934.214321

UB3LYP-D3/def2TZVPP-CPCM(ACN)

E(scf)= -935.522276275

UM062X-D3/def2TZVPP-CPCM(ACN)

E(scf)= -935.098953022

UTPSSH/def2TZVPP-CPCM(ACN)

E(scf)= -935.524429619

UM06-D3/def2TZVPP-CPCM(ACN)

E(scf)= -934.83318788

**^3^B’H^+^**

E(scf) = -934.420209280a.u.

ν_min_ = 18.6351cm^-1^

| C | -0.026962 | -2.953196 | -2.828697 |
| --- | --- | --- | --- |
| C | -0.907649 | -1.757315 | -3.128219 |
| N | -1.379476 | -1.247799 | -1.899346 |
| C | -0.926468 | -2.054280 | -0.762930 |
| C | -0.458110 | -3.381265 | -1.427120 |
| O | -1.183077 | -1.277601 | -4.206698 |
| C | -2.302823 | -0.207551 | -1.891387 |
| C | 0.184287 | -1.344180 | -0.005468 |
| C | 0.626502 | -1.795246 | 1.247656 |
| O | 0.786100 | -0.274457 | -0.537004 |
| N | 0.126789 | -2.877216 | 1.922082 |
| C | 0.783452 | -3.030515 | 3.117447 |
| C | 1.711268 | -2.028615 | 3.190514 |
| N | 1.613757 | -1.271444 | 2.042720 |
| C | 2.434044 | -0.096412 | 1.749042 |
| C | -2.864514 | 0.518769 | -0.808507 |
| C | -2.528832 | 0.404791 | 0.575181 |
| C | -3.155606 | 1.194570 | 1.534658 |
| C | -4.137311 | 2.128521 | 1.174791 |
| C | -4.482709 | 2.266179 | -0.180631 |
| C | -3.864880 | 1.486981 | -1.147928 |
| H | -0.160786 | -3.723395 | -3.599586 |
| H | 1.027160 | -2.627798 | -2.862780 |
| H | -1.772860 | -2.241411 | -0.084322 |
| H | 0.337533 | -3.870391 | -0.850583 |
| H | -1.316027 | -4.067142 | -1.480023 |
| H | -2.598088 | 0.061824 | -2.906477 |
| H | 0.331153 | -0.009754 | -1.355768 |
| H | 0.544178 | -3.828965 | 3.813127 |
| H | 2.431935 | -1.795598 | 3.968931 |
| H | 1.794098 | 0.785200 | 1.620530 |
| H | 3.116969 | 0.060745 | 2.591257 |
| H | 3.009958 | -0.258937 | 0.829677 |
| H | -1.761664 | -0.287235 | 0.912717 |
| H | -2.868614 | 1.082144 | 2.583381 |
| H | -4.622826 | 2.743019 | 1.936182 |
| H | -5.242822 | 2.992552 | -0.479662 |
| H | -4.144801 | 1.606900 | -2.197786 |
| H | -0.625668 | -3.476593 | 1.598918 |


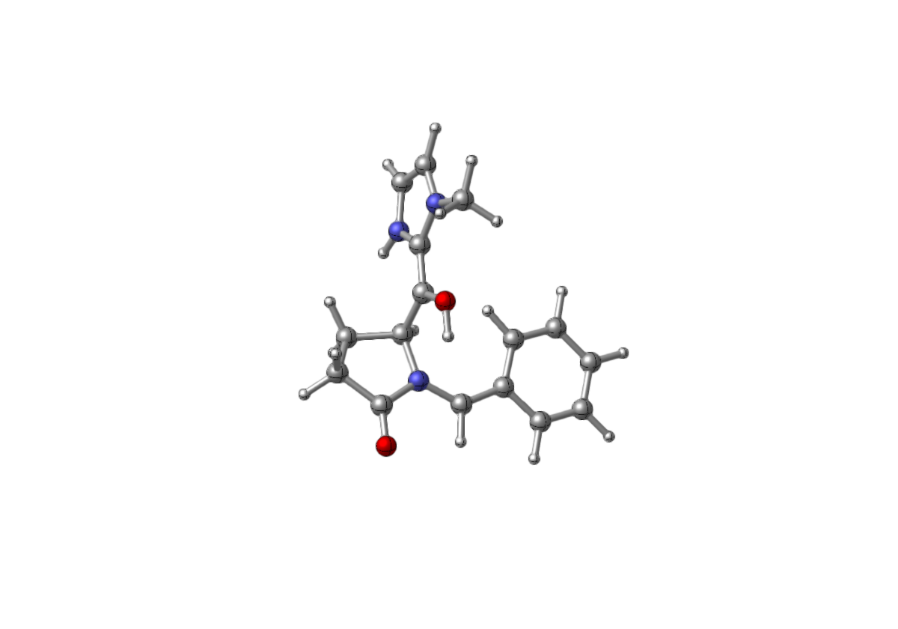


Zero-point correction= 0.320500 (Hartree/Particle)

Thermal correction to Energy= 0.339278

Thermal correction to Enthalpy= 0.340222

Thermal correction to Gibbs Free Energy= 0.270948

Sum of electronic and zero-point Energies= -934.099709

Sum of electronic and thermal Energies= -934.080931

Sum of electronic and thermal Enthalpies= -934.079987

Sum of electronic and thermal Free Energies= -934.149261

E(scf) = -934.390356524 a.u.

ν_min_ = -224.8752cm^-1^

| C | -0.459278 | -3.274337 | -2.434730 |
| --- | --- | --- | --- |
| C | -1.154392 | -1.996507 | -2.841281 |
| N | -1.508799 | -1.306444 | -1.664445 |
| C | -1.009949 | -1.938496 | -0.464814 |
| C | -0.692089 | -3.387954 | -0.925563 |
| O | -1.371789 | -1.564367 | -3.949026 |
| C | -1.750299 | 0.086651 | -1.686754 |
| C | 0.209801 | -1.117221 | 0.089866 |
| C | 0.931740 | -1.633086 | 1.188749 |
| O | 0.481030 | 0.059194 | -0.371507 |
| N | 0.656189 | -2.766785 | 1.910369 |
| C | 1.581799 | -2.919981 | 2.919827 |
| C | 2.451197 | -1.874262 | 2.823113 |
| N | 2.052646 | -1.082664 | 1.761827 |
| C | 2.738138 | 0.126487 | 1.317718 |
| C | -2.817027 | 0.626839 | -0.788135 |
| C | -3.861960 | -0.184624 | -0.310618 |
| C | -4.859152 | 0.360002 | 0.500488 |
| C | -4.821097 | 1.715630 | 0.849718 |
| C | -3.783318 | 2.529461 | 0.377044 |
| C | -2.786104 | 1.989059 | -0.434544 |
| H | -0.839635 | -4.124142 | -3.017421 |
| H | 0.607586 | -3.153409 | -2.687724 |
| H | -1.777854 | -1.925692 | 0.326097 |
| H | 0.170192 | -3.819488 | -0.405441 |
| H | -1.565972 | -4.020117 | -0.713446 |
| H | -1.799523 | 0.446196 | -2.726225 |
| H | -0.710039 | 0.465953 | -1.251297 |
| H | 1.545128 | -3.750985 | 3.617429 |
| H | 3.324208 | -1.629692 | 3.421091 |
| H | 2.076573 | 0.996613 | 1.416480 |
| H | 3.628537 | 0.265493 | 1.941968 |
| H | 3.038083 | 0.026737 | 0.266334 |
| H | -3.907909 | -1.236987 | -0.599256 |
| H | -5.674471 | -0.274864 | 0.855281 |
| H | -5.601260 | 2.138430 | 1.487146 |
| H | -3.750658 | 3.587694 | 0.646311 |
| H | -1.971266 | 2.622136 | -0.795415 |
| H | -0.156598 | -3.358690 | 1.786575 |


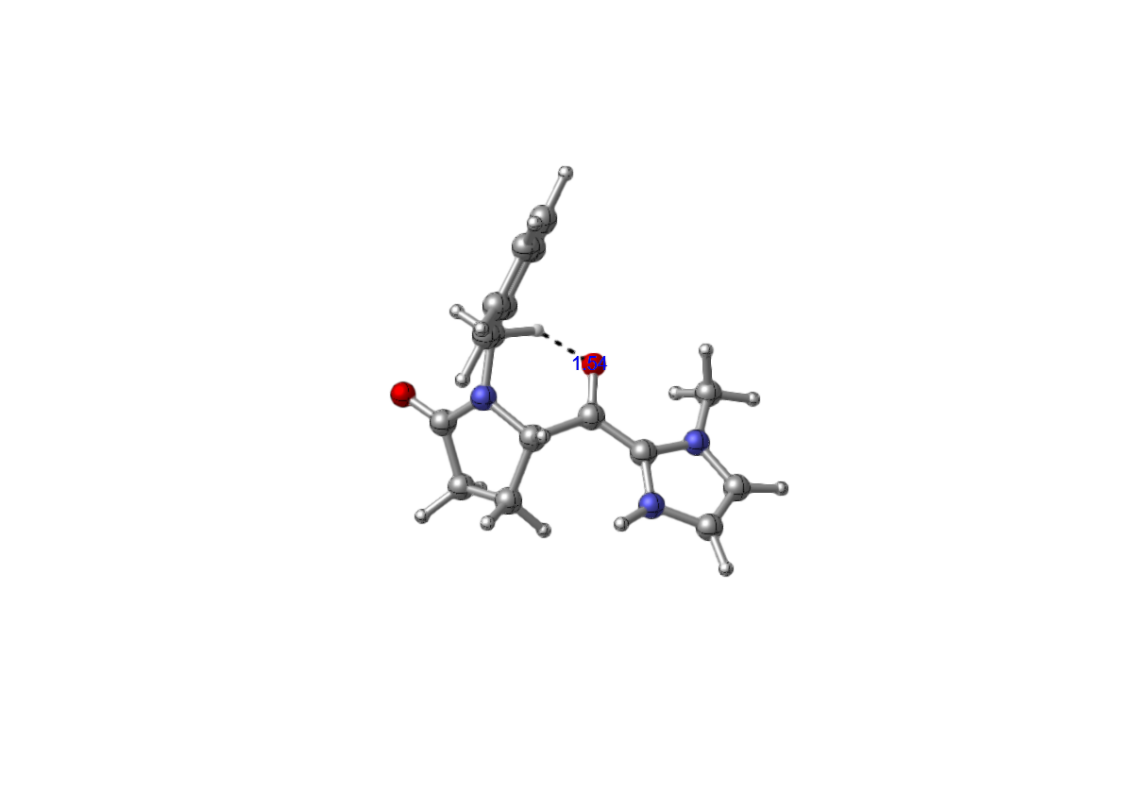


Zero-point correction= 0.317170 (Hartree/Particle)

Thermal correction to Energy= 0.335118

Thermal correction to Enthalpy= 0.336062

Thermal correction to Gibbs Free Energy= 0.268569

Sum of electronic and zero-point Energies= -934.073187

Sum of electronic and thermal Energies= -934.055238

Sum of electronic and thermal Enthalpies= -934.054294

Sum of electronic and thermal Free Energies= -934.121787

UB3LYP-D3/def2TZVPP-CPCM(ACN)

E(scf)= -935.418269009

UM062X-D3/def2TZVPP-CPCM(ACN)

E(scf)= -934.980095316

UTPSSH/def2TZVPP-CPCM(ACN)

E(scf)= -935.430909514

UM06-D3/def2TZVPP-CPCM(ACN)

E(scf)= -934.721830471

**TS2H^+^**

E(scf) = -934.417160416 a.u.

ν_min_ = -351.0111cm^-1^

| C | -1.033467 | -3.389541 | -3.372040 |
| --- | --- | --- | --- |
| C | -1.827450 | -2.110479 | -3.301756 |
| N | -1.798690 | -1.612561 | -1.972862 |
| C | -0.732434 | -2.655742 | -1.035116 |
| C | -0.021843 | -3.282803 | -2.239112 |
| O | -2.442726 | -1.567045 | -4.189569 |
| C | -1.975777 | -0.315136 | -1.760252 |
| C | 0.035261 | -1.871039 | -0.122431 |
| C | -0.002772 | -2.000275 | 1.290461 |
| O | 0.830036 | -0.888814 | -0.647308 |
| N | -0.498617 | -3.060281 | 1.989030 |
| C | -0.348285 | -2.852974 | 3.349057 |
| C | 0.267413 | -1.650166 | 3.492653 |
| N | 0.479977 | -1.126089 | 2.221618 |
| C | 1.028206 | 0.202426 | 1.961737 |
| C | -2.211962 | 0.242514 | -0.465502 |
| C | -2.547586 | -0.579263 | 0.639171 |
| C | -2.692932 | -0.023429 | 1.911793 |
| C | -2.514140 | 1.349173 | 2.098008 |
| C | -2.204935 | 2.183063 | 1.006133 |
| C | -2.059529 | 1.641263 | -0.262317 |
| H | -1.734815 | -4.228891 | -3.218671 |
| H | -0.590309 | -3.505686 | -4.369670 |
| H | -1.434377 | -3.351010 | -0.562067 |
| H | 0.819820 | -2.638183 | -2.531352 |
| H | 0.383382 | -4.258274 | -1.934132 |
| H | -1.920650 | 0.346343 | -2.634964 |
| H | 0.303307 | -0.289976 | -1.215607 |
| H | -0.678876 | -3.578940 | 4.085035 |
| H | 0.571952 | -1.113569 | 4.386021 |
| H | 1.189187 | 0.697648 | 2.926345 |
| H | 1.976519 | 0.130275 | 1.416355 |
| H | 0.319100 | 0.793383 | 1.368164 |
| H | -2.758633 | -1.635507 | 0.479430 |
| H | -2.951286 | -0.663789 | 2.757400 |
| H | -2.624374 | 1.781882 | 3.095010 |
| H | -2.077710 | 3.256611 | 1.159422 |
| H | -1.808065 | 2.281328 | -1.111511 |
| H | -0.870779 | -3.904542 | 1.570256 |


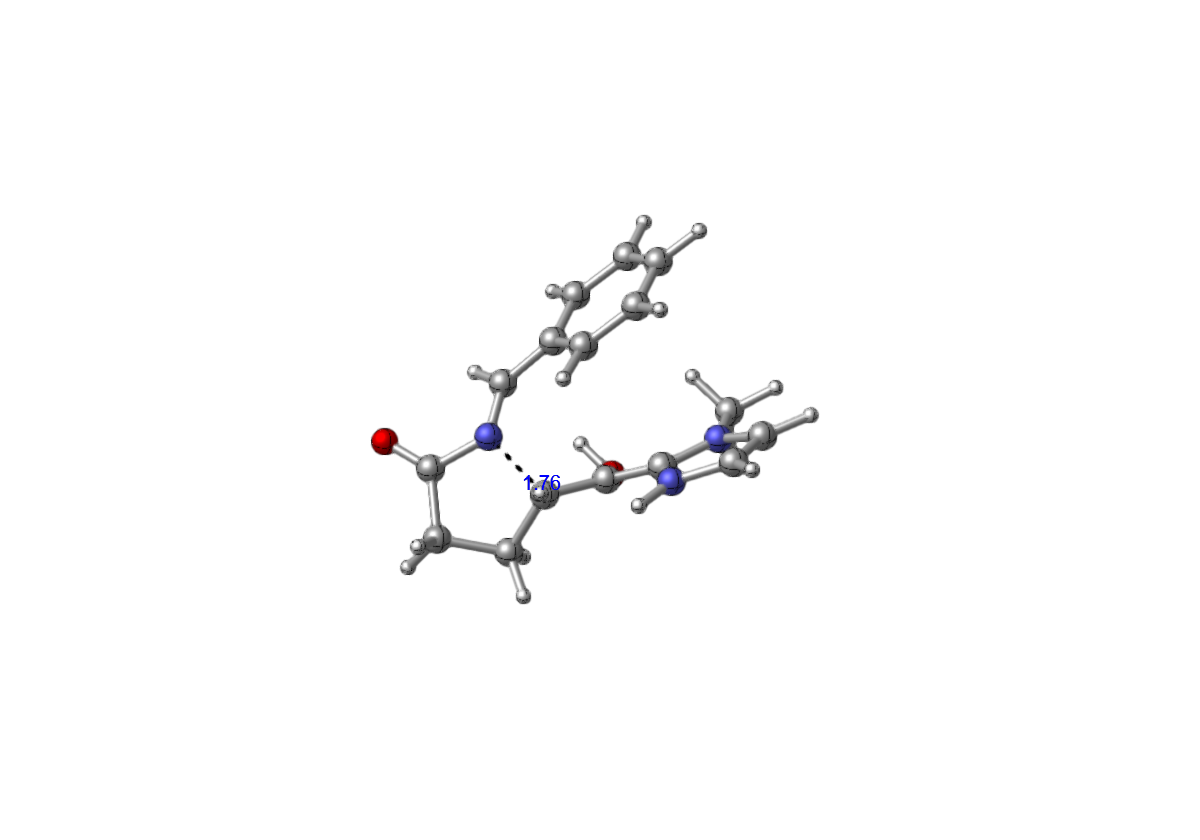


Zero-point correction= 0.319617 (Hartree/Particle)

Thermal correction to Energy= 0.337777

Thermal correction to Enthalpy= 0.338721

Thermal correction to Gibbs Free Energy= 0.273763

Sum of electronic and zero-point Energies= -934.097544

Sum of electronic and thermal Energies= -934.079383

Sum of electronic and thermal Enthalpies= -934.078439

Sum of electronic and thermal Free Energies= -934.143397

UB3LYP-D3/def2TZVPP-CPCM(ACN)

E(scf) = -935.447073459

UM062X-D3/def2TZVPP-CPCM(ACN)

E(scf)= -935.015016948

UTPSSH/def2TZVPP-CPCM(ACN)

E(scf)= -935.448339214

UM06-D3/def2TZVPP-CPCM(ACN)

E(scf)= -934.756367735

**TS2’H^+^**

E(scf) = -934.412535787a.u.

ν_min_ = -409.0632cm^-1^

| C | -1.361383 | -3.192402 | -3.471583 |
| --- | --- | --- | --- |
| C | -1.851405 | -1.761983 | -3.263892 |
| N | -1.681775 | -1.480628 | -1.924861 |
| C | -0.896632 | -2.470754 | -1.194706 |
| C | -0.414333 | -3.457695 | -2.286179 |
| O | -2.304324 | -0.988333 | -4.084920 |
| C | -1.655380 | -0.198193 | -1.327621 |
| C | 0.158375 | -1.616882 | -0.481752 |
| C | 0.146753 | -1.388753 | 0.913449 |
| O | 1.291320 | -1.275467 | -1.128579 |
| N | -0.737290 | -1.916961 | 1.805954 |
| C | -0.549732 | -1.365255 | 3.049076 |
| C | 0.478550 | -0.473370 | 2.927774 |
| N | 0.902746 | -0.489111 | 1.615341 |
| C | 1.929920 | 0.394859 | 1.065825 |
| C | -2.390292 | 0.106635 | -0.141343 |
| C | -3.308889 | -0.809470 | 0.436769 |
| C | -3.925476 | -0.523416 | 1.652797 |
| C | -3.638387 | 0.669666 | 2.329047 |
| C | -2.751322 | 1.598386 | 1.757691 |
| C | -2.142163 | 1.330372 | 0.538586 |
| H | -2.238331 | -3.860959 | -3.454248 |
| H | -0.889406 | -3.289712 | -4.458584 |
| H | -1.516149 | -3.000537 | -0.455670 |
| H | 0.624005 | -3.231454 | -2.570121 |
| H | -0.435026 | -4.495632 | -1.930720 |
| H | -1.230502 | 0.609709 | -1.931528 |
| H | 1.137229 | -1.155827 | -2.079102 |
| H | -1.150378 | -1.649184 | 3.907157 |
| H | 0.936790 | 0.175639 | 3.668103 |
| H | 2.262544 | 1.067606 | 1.864214 |
| H | 2.777758 | -0.191009 | 0.692321 |
| H | 1.508020 | 0.982271 | 0.239913 |
| H | -3.546141 | -1.734507 | -0.093693 |
| H | -4.632275 | -1.237992 | 2.081433 |
| H | -4.112844 | 0.884104 | 3.289314 |
| H | -2.535702 | 2.535297 | 2.276970 |
| H | -1.442237 | 2.048657 | 0.103863 |
| H | -1.413889 | -2.640949 | 1.592925 |


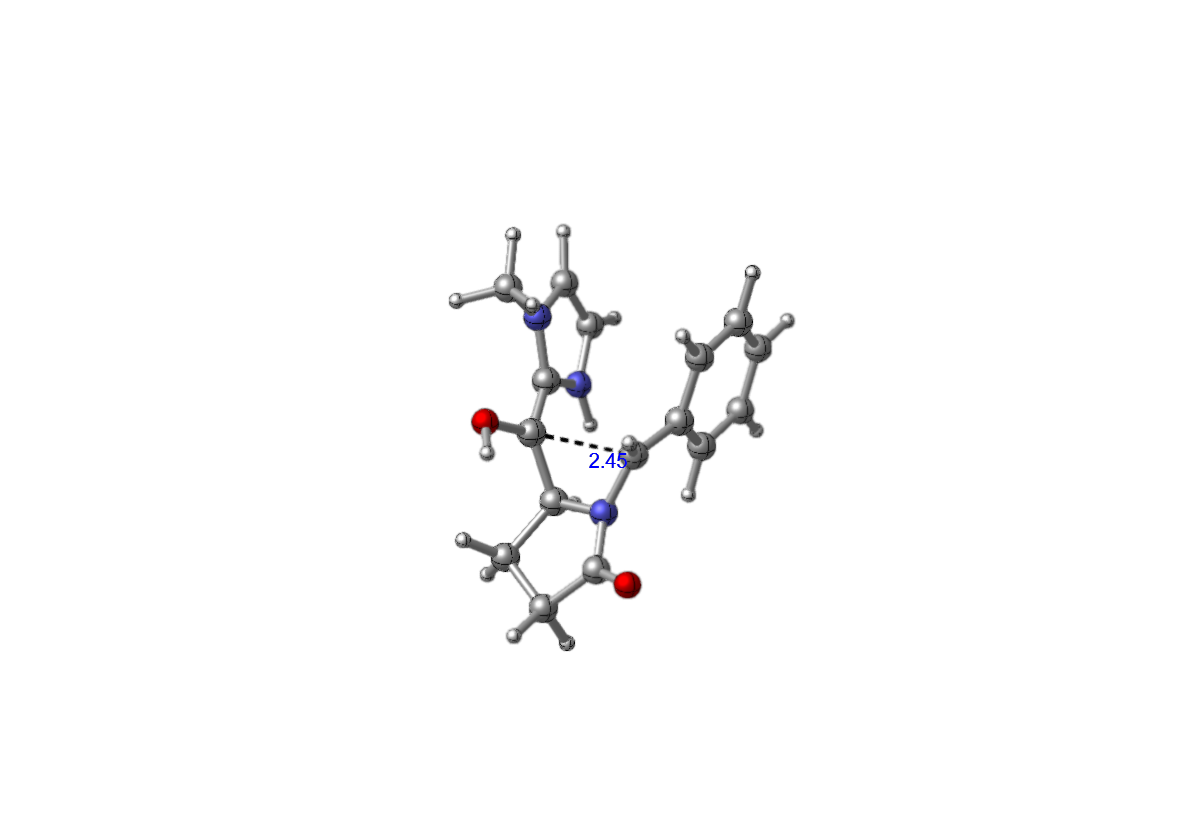


Zero-point correction= 0.319678 (Hartree/Particle)

Thermal correction to Energy= 0.338043

Thermal correction to Enthalpy= 0.338988

Thermal correction to Gibbs Free Energy= 0.272260

Sum of electronic and zero-point Energies= -934.092858

Sum of electronic and thermal Energies= -934.074492

Sum of electronic and thermal Enthalpies= -934.073548

Sum of electronic and thermal Free Energies= -934.140276

UB3LYP-D3/def2TZVPP-CPCM(ACN)

E(scf)= -935.442185656

UM062X-D3/def2TZVPP-CPCM(ACN)

E(scf)= -935.010545693

UTPSSH/def2TZVPP-CPCM(ACN)

E(scf)= -935.445089270

UM06-D3/def2TZVPP-CPCM(ACN)

E(scf)= -934.753393563

**TS3H^+^**

E(scf) = -934.434519147a.u.

ν_min_ = -194.1584cm^-1^

| C | -1.079713 | -3.579218 | -3.326926 |
| --- | --- | --- | --- |
| C | -2.072727 | -2.460005 | -3.361779 |
| N | -2.582760 | -2.147769 | -2.044099 |
| C | 0.101283 | -2.503969 | -1.207456 |
| C | 0.208265 | -3.047155 | -2.617064 |
| O | -2.317520 | -1.738483 | -4.293509 |
| C | -1.948621 | -1.135333 | -1.427779 |
| C | -0.585081 | -3.177312 | -0.184626 |
| C | -0.306522 | -2.785216 | 1.228961 |
| O | -1.513823 | -4.014811 | -0.374482 |
| N | 0.611309 | -1.901129 | 1.668522 |
| C | 0.570773 | -1.802660 | 3.035113 |
| C | -0.405474 | -2.665177 | 3.449542 |
| N | -0.932573 | -3.267222 | 2.324686 |
| C | -2.003243 | -4.269697 | 2.368087 |
| C | -2.289902 | -0.693919 | -0.087108 |
| C | -3.311530 | -1.317128 | 0.660110 |
| C | -3.561627 | -0.915649 | 1.971757 |
| C | -2.805327 | 0.111402 | 2.549769 |
| C | -1.808520 | 0.756224 | 1.804283 |
| C | -1.553459 | 0.360613 | 0.494218 |
| H | -1.475893 | -4.438951 | -2.770682 |
| H | -0.830388 | -3.873108 | -4.354663 |
| H | 0.870185 | -1.773425 | -0.947430 |
| H | 0.662801 | -2.276093 | -3.258368 |
| H | 0.921647 | -3.891568 | -2.604328 |
| H | -1.390277 | -0.448369 | -2.068789 |
| H | -2.701593 | -2.981236 | -1.430115 |
| H | 1.224224 | -1.139280 | 3.593011 |
| H | -0.764232 | -2.898835 | 4.447300 |
| H | -2.211228 | -4.490421 | 3.421055 |
| H | -2.900281 | -3.874233 | 1.880157 |
| H | -1.684116 | -5.173702 | 1.840459 |
| H | -3.921252 | -2.102976 | 0.211316 |
| H | -4.351642 | -1.403765 | 2.546406 |
| H | -2.999928 | 0.417909 | 3.579964 |
| H | -1.226067 | 1.564167 | 2.251883 |
| H | -0.763987 | 0.848255 | -0.082964 |
| H | 1.246830 | -1.384980 | 1.067897 |


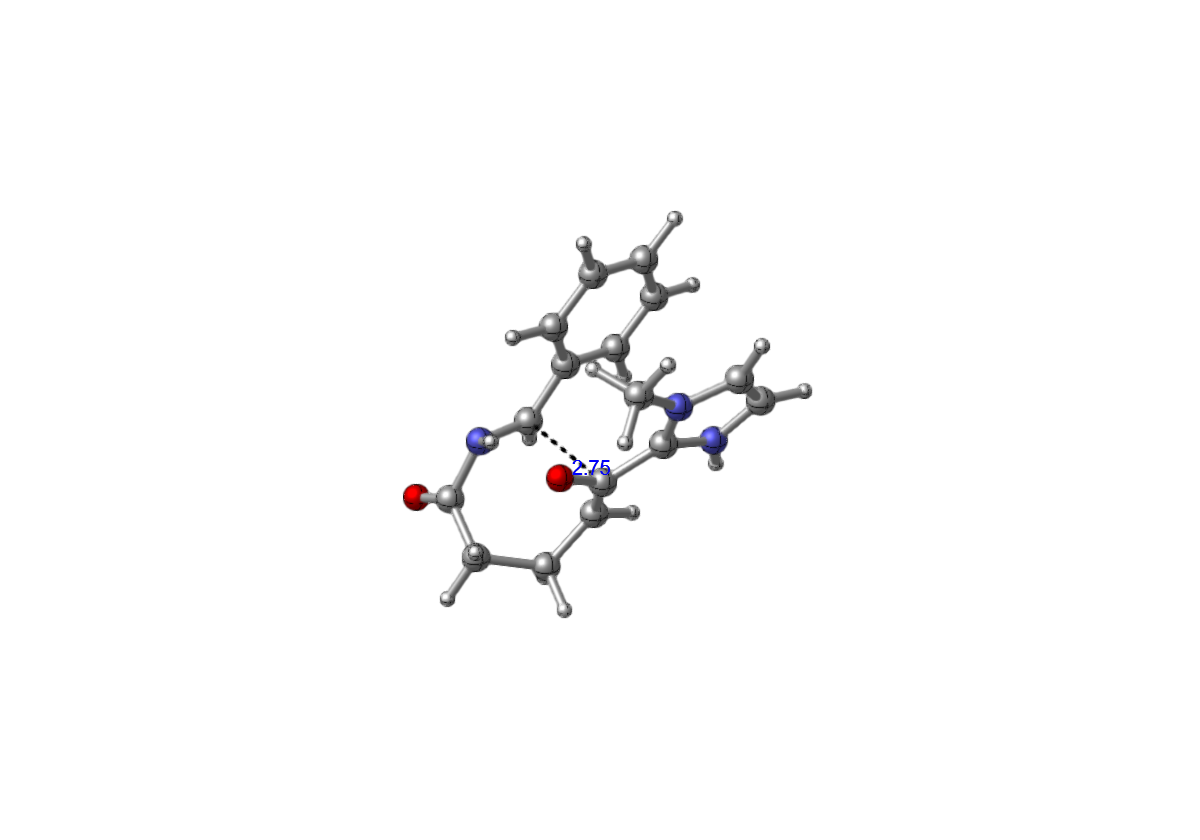


Zero-point correction= 0.321095 (Hartree/Particle)

Thermal correction to Energy= 0.338971

Thermal correction to Enthalpy= 0.339915

Thermal correction to Gibbs Free Energy= 0.274801

Sum of electronic and zero-point Energies= -934.113424

Sum of electronic and thermal Energies= -934.095548

Sum of electronic and thermal Enthalpies= -934.094604

Sum of electronic and thermal Free Energies= -934.159719

UB3LYP-D3/def2TZVPP-CPCM(ACN)

E(scf)= -935.465606457

UM062X-D3/def2TZVPP-CPCM(ACN)

E(scf)= -935.032032331

UTPSSH/def2TZVPP-CPCM(ACN)

E(scf)= -935.461734054

UM06-D3/def2TZVPP-CPCM(ACN)

E(scf)= -934.770568315

**TS4H^+^**

E(scf) = -934.424070675 a.u.

ν_min_ = -190.0357cm^-1^

| C | -0.592672 | -3.408496 | -3.250491 |
| --- | --- | --- | --- |
| C | -1.861930 | -2.614529 | -3.361660 |
| N | -2.384174 | -2.229352 | -2.050454 |
| C | 0.278972 | -2.376378 | -0.985960 |
| C | 0.511468 | -2.664520 | -2.449520 |
| O | -2.431724 | -2.301118 | -4.371420 |
| C | -1.910042 | -1.192898 | -1.356319 |
| C | -0.454427 | -3.169688 | -0.102533 |
| C | -0.318527 | -2.875943 | 1.356913 |
| O | -1.326283 | -4.039707 | -0.428617 |
| N | 0.671706 | -2.188935 | 1.960098 |
| C | 0.469764 | -2.143194 | 3.317328 |
| C | -0.685817 | -2.832782 | 3.552846 |
| N | -1.157752 | -3.281697 | 2.333785 |
| C | -2.385980 | -4.070187 | 2.177005 |
| C | -1.372560 | 0.072353 | -1.836547 |
| C | -1.341544 | 0.476011 | -3.189284 |
| C | -0.789184 | 1.709655 | -3.537296 |
| C | -0.263569 | 2.554865 | -2.554721 |
| C | -0.304532 | 2.172135 | -1.206510 |
| C | -0.859748 | 0.948246 | -0.850146 |
| H | -0.825856 | -4.359111 | -2.746682 |
| H | -0.242984 | -3.616086 | -4.270575 |
| H | 0.925179 | -1.595847 | -0.579242 |
| H | 0.744209 | -1.714257 | -2.960704 |
| H | 1.428334 | -3.274435 | -2.542612 |
| H | -2.183945 | -1.209619 | -0.298864 |
| H | -2.419697 | -3.077170 | -1.427417 |
| H | 1.154184 | -1.640610 | 3.993812 |
| H | -1.207287 | -3.042369 | 4.482107 |
| H | -3.097624 | -3.534452 | 1.538946 |
| H | -2.149022 | -5.031004 | 1.709964 |
| H | -2.816539 | -4.218606 | 3.173683 |
| H | -1.779040 | -0.145754 | -3.966577 |
| H | -0.777897 | 2.015658 | -4.585630 |
| H | 0.169567 | 3.517479 | -2.836464 |
| H | 0.095979 | 2.833350 | -0.435145 |
| H | -0.884252 | 0.647425 | 0.200146 |
| H | 1.469599 | -1.790417 | 1.473818 |


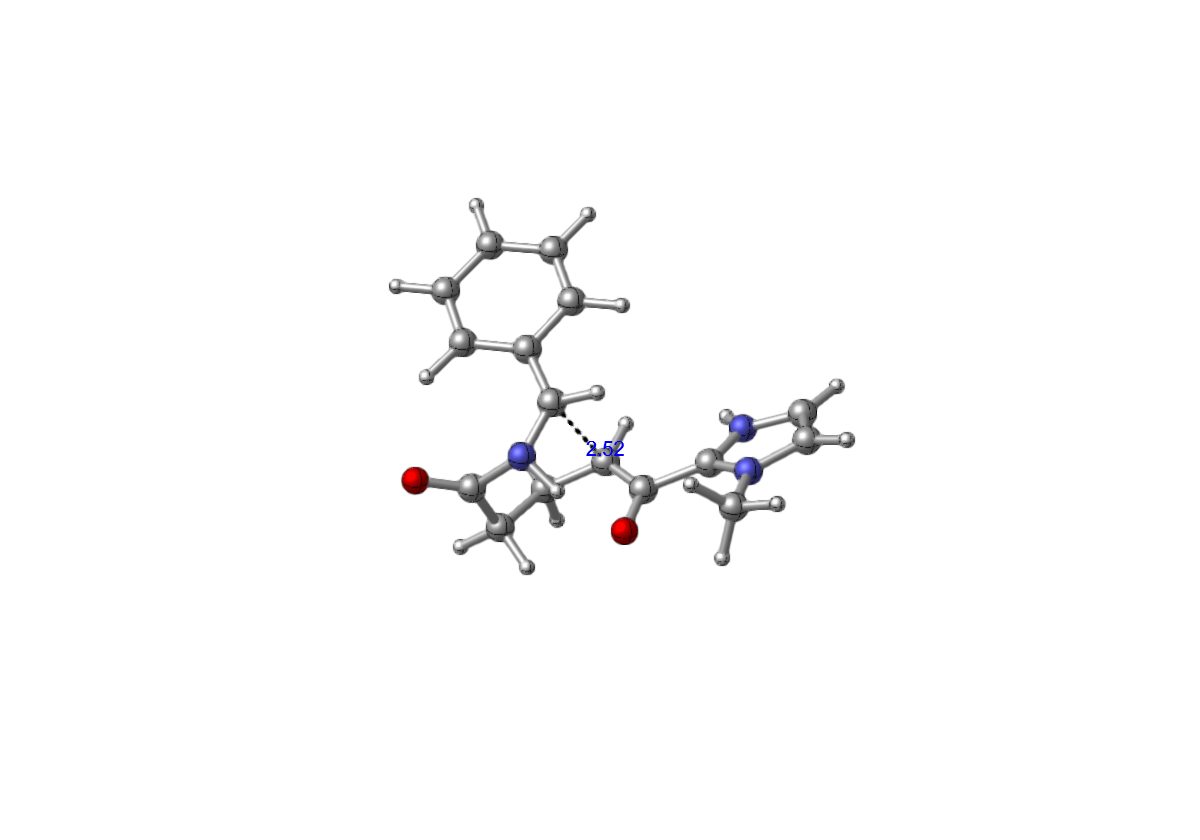


Zero-point correction= 0.320829 (Hartree/Particle)

Thermal correction to Energy= 0.338804

Thermal correction to Enthalpy= 0.339748

Thermal correction to Gibbs Free Energy= 0.273764

Sum of electronic and zero-point Energies= -934.103241

Sum of electronic and thermal Energies= -934.085267

Sum of electronic and thermal Enthalpies= -934.084322

Sum of electronic and thermal Free Energies= -934.150306

UB3LYP-D3/def2TZVPP-CPCM(ACN)

E(scf)= -935.455200895

UM062X-D3/def2TZVPP-CPCM(ACN)

E(scf)= -935.020143007

UTPSSH/def2TZVPP-CPCM(ACN)

E(scf)= -935.456770798

UM06-D3/def2TZVPP-CPCM(ACN)

E(scf)= -934.759035061

**^3-1^MECP (protonated)**

E(scf) = -934.420658832 a.u.

ν_min_ = 23.1232cm^-1^

| C | -1.109960 | -3.338609 | -3.436158 |
| --- | --- | --- | --- |
| C | -1.767930 | -1.972571 | -3.446277 |
| N | -1.709052 | -1.463906 | -2.122952 |
| C | -0.983341 | -2.361612 | -1.209203 |
| C | -0.237568 | -3.317892 | -2.182216 |
| O | -2.267598 | -1.372959 | -4.360571 |
| C | -2.149757 | -0.180588 | -1.846546 |
| C | -0.074909 | -1.575099 | -0.308334 |
| C | 0.067093 | -1.831723 | 1.063727 |
| O | 0.631628 | -0.560038 | -0.823865 |
| N | -0.366407 | -2.952572 | 1.721452 |
| C | -0.108480 | -2.841110 | 3.070805 |
| C | 0.495755 | -1.630169 | 3.253499 |
| N | 0.606457 | -1.014258 | 2.022069 |
| C | 1.050494 | 0.367074 | 1.827768 |
| C | -2.414230 | 0.357622 | -0.555325 |
| C | -2.676849 | -0.430070 | 0.601477 |
| C | -2.858442 | 0.164594 | 1.846542 |
| C | -2.796637 | 1.557007 | 1.986719 |
| C | -2.585771 | 2.355823 | 0.850912 |
| C | -2.407911 | 1.773598 | -0.396890 |
| H | -1.903830 | -4.103343 | -3.382445 |
| H | -0.557771 | -3.503874 | -4.370511 |
| H | -1.700476 | -2.932581 | -0.594525 |
| H | 0.749688 | -2.889551 | -2.410483 |
| H | -0.078340 | -4.305214 | -1.728333 |
| H | -2.305272 | 0.417770 | -2.747198 |
| H | 0.173473 | -0.197862 | -1.604952 |
| H | -0.360578 | -3.622516 | 3.781860 |
| H | 0.853746 | -1.157073 | 4.163454 |
| H | 1.345557 | 0.765407 | 2.804984 |
| H | 1.901334 | 0.403902 | 1.138214 |
| H | 0.223514 | 0.967253 | 1.423378 |
| H | -2.807796 | -1.507353 | 0.506124 |
| H | -3.078854 | -0.460331 | 2.715216 |
| H | -2.945722 | 2.019855 | 2.964275 |
| H | -2.567644 | 3.444020 | 0.946514 |
| H | -2.241529 | 2.402989 | -1.274401 |
| H | -0.763460 | -3.767501 | 1.269313 |


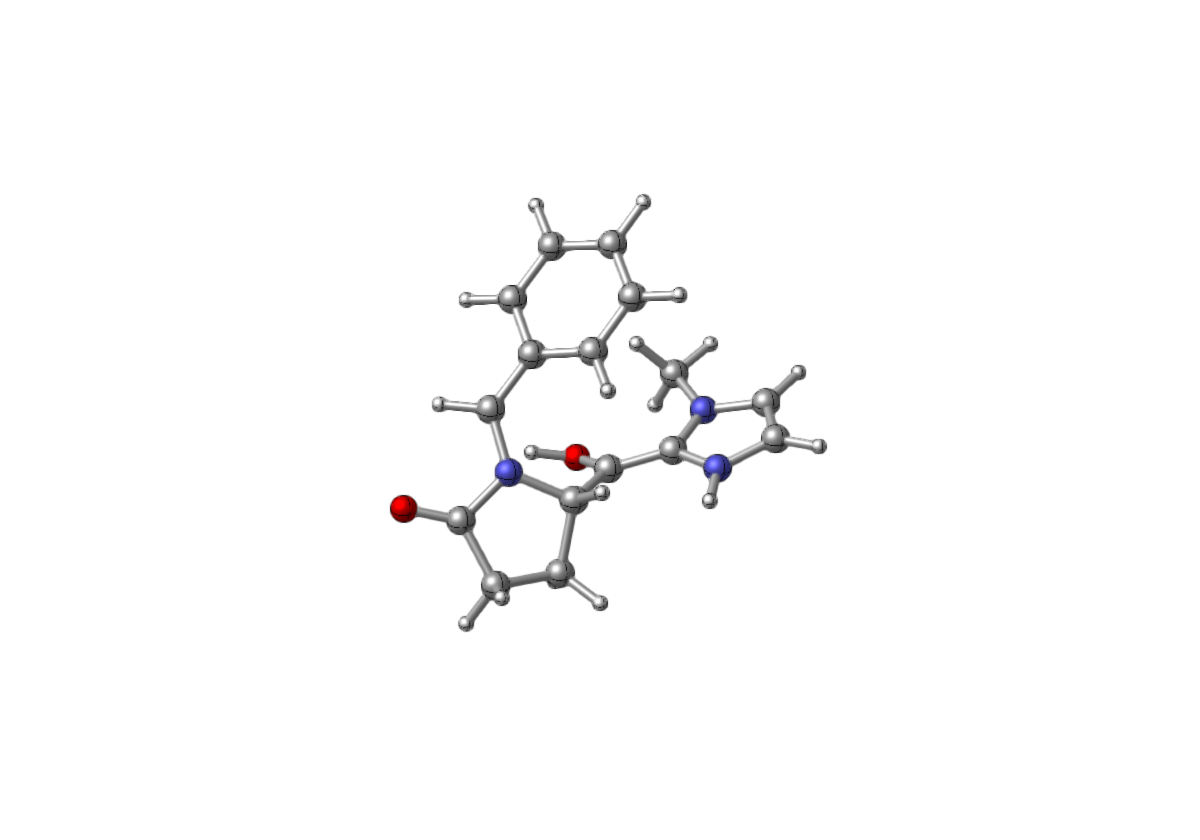


Zero-point correction= 0.320494 (Hartree/Particle)

Thermal correction to Energy= 0.339256

Thermal correction to Enthalpy= 0.340201

Thermal correction to Gibbs Free Energy= 0.271374

Sum of electronic and zero-point Energies= -934.100165

Sum of electronic and thermal Energies= -934.081402

Sum of electronic and thermal Enthalpies= -934.080458

Sum of electronic and thermal Free Energies= -934.149285

UB3LYP-D3/def2TZVPP-CPCM(ACN)

E(scf)= -935.449073848

UM062X-D3/def2TZVPP-CPCM(ACN)

E(scf)= -935.021091726

UTPSSH/def2TZVPP-CPCM(ACN)

E(scf)= -935.452758797

UM06-D3/def2TZVPP-CPCM(ACN)

E(scf)= -934.761222525

**C’’’H+**

E(scf) = -934.448462104 a.u.

ν_min_ = 23.4221cm^-1^

| C | -1.400003 | -3.225819 | -3.410219 |
| --- | --- | --- | --- |
| C | -2.417687 | -2.106255 | -3.584919 |
| N | -2.978134 | -1.633324 | -2.417322 |
| C | -0.248082 | -2.655288 | -1.183882 |
| C | -0.105820 | -2.843952 | -2.664384 |
| O | -2.808663 | -1.745105 | -4.683987 |
| C | -2.812148 | -0.564605 | -1.746707 |
| C | 0.386047 | -1.723904 | -0.440495 |
| C | 0.316073 | -1.738925 | 1.023676 |
| O | 1.171052 | -0.716441 | -0.905315 |
| N | 0.002640 | -2.812204 | 1.775884 |
| C | 0.020993 | -2.484127 | 3.109241 |
| C | 0.369540 | -1.165916 | 3.173285 |
| N | 0.549111 | -0.718030 | 1.876969 |
| C | 0.938893 | 0.656300 | 1.541082 |
| C | -1.842444 | 0.529959 | -1.951676 |
| C | -0.984743 | 0.616316 | -3.069093 |
| C | -0.059467 | 1.656200 | -3.167091 |
| C | 0.014082 | 2.633618 | -2.164428 |
| C | -0.852768 | 2.575354 | -1.069429 |
| C | -1.772979 | 1.529270 | -0.963500 |
| H | -1.892512 | -4.059592 | -2.881874 |
| H | -1.150874 | -3.570549 | -4.422989 |
| H | -0.892441 | -3.371602 | -0.668716 |
| H | 0.366890 | -1.974299 | -3.147026 |
| H | 0.604197 | -3.677916 | -2.816701 |
| H | -3.459925 | -0.434951 | -0.865154 |
| H | 0.936479 | -0.483829 | -1.817429 |
| H | -0.202326 | -3.204857 | 3.889716 |
| H | 0.505963 | -0.511132 | 4.028718 |
| H | 0.789369 | 1.276230 | 2.431830 |
| H | 1.991737 | 0.680959 | 1.235816 |
| H | 0.318603 | 1.027569 | 0.721378 |
| H | -1.055189 | -0.109403 | -3.878401 |
| H | 0.600945 | 1.711877 | -4.035252 |
| H | 0.740242 | 3.445543 | -2.246135 |
| H | -0.806361 | 3.340323 | -0.291301 |
| H | -2.439082 | 1.476870 | -0.098385 |
| H | -0.181168 | -3.739704 | 1.403305 |


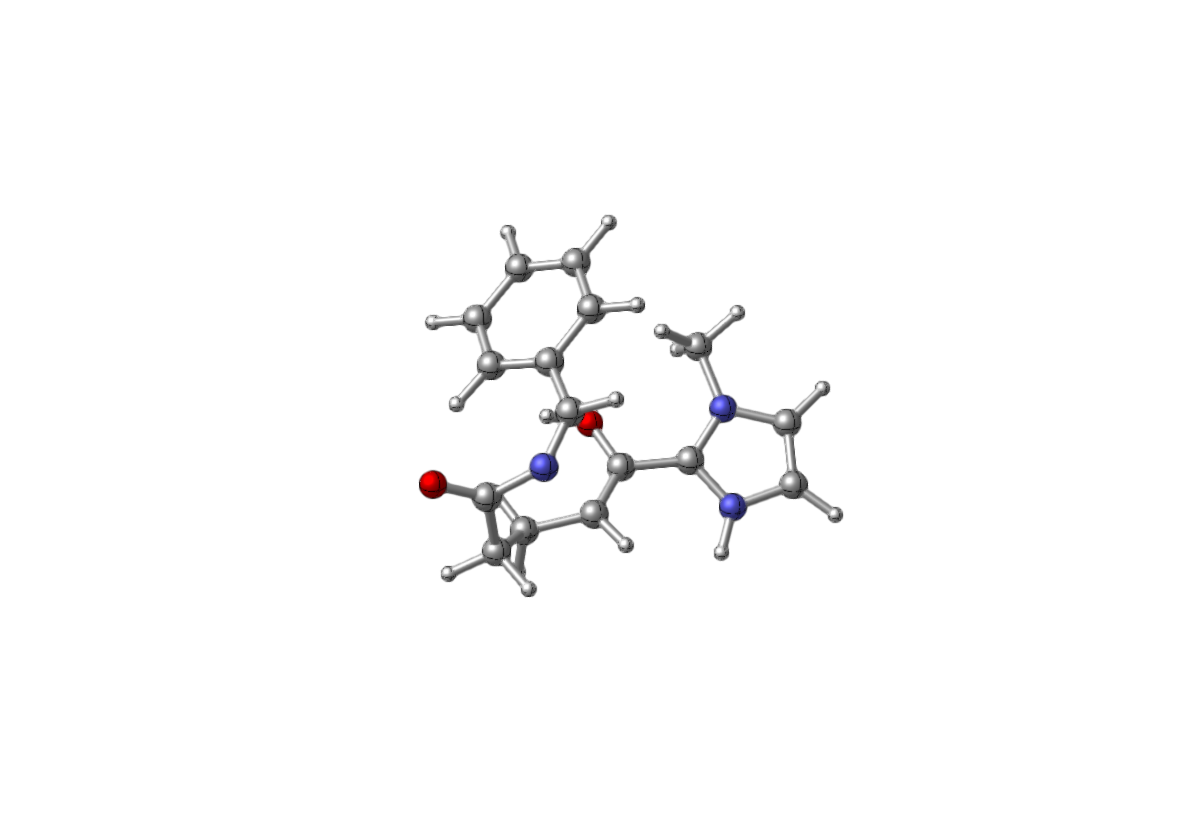


Zero-point correction= 0.321095 (Hartree/Particle)

Thermal correction to Energy= 0.340377

Thermal correction to Enthalpy= 0.341322

Thermal correction to Gibbs Free Energy= 0.272435

Sum of electronic and zero-point Energies= -934.127367

Sum of electronic and thermal Energies= -934.108085

Sum of electronic and thermal Enthalpies= -934.107141

Sum of electronic and thermal Free Energies= -934.176027

**C’’’’H+**

E(scf) = -934.444748458 a.u.

ν_min_ = 14.9065cm^-1^

| C | -1.827900 | -3.098217 | -2.967036 |
| --- | --- | --- | --- |
| C | -1.715658 | -1.583627 | -3.033517 |
| N | -2.355967 | -0.904029 | -1.995528 |
| C | 0.095859 | -3.172274 | -1.284278 |
| C | -0.467741 | -3.725894 | -2.575068 |
| O | -1.122038 | -1.011724 | -3.928648 |
| C | -1.700115 | -0.123005 | -1.221589 |
| C | -0.402554 | -3.348771 | -0.046930 |
| C | -1.534887 | -4.247369 | 0.243163 |
| O | 0.018684 | -2.720826 | 1.083992 |
| N | -1.610994 | -5.553656 | -0.061100 |
| C | -2.818010 | -6.066254 | 0.350380 |
| C | -3.496552 | -5.030295 | 0.929899 |
| N | -2.685131 | -3.915240 | 0.858332 |
| C | -3.074802 | -2.563711 | 1.270943 |
| C | -2.318172 | 0.660317 | -0.148196 |
| C | -3.718231 | 0.740077 | -0.005814 |
| C | -4.273843 | 1.468590 | 1.043111 |
| C | -3.440890 | 2.122970 | 1.963475 |
| C | -2.050347 | 2.051770 | 1.828315 |
| C | -1.490351 | 1.325951 | 0.774266 |
| H | -2.610939 | -3.388749 | -2.252235 |
| H | -2.117762 | -3.466574 | -3.963360 |
| H | 0.939212 | -2.477921 | -1.369274 |
| H | 0.253471 | -3.525614 | -3.379993 |
| H | -0.591468 | -4.818634 | -2.538601 |
| H | -0.602346 | -0.033562 | -1.313450 |
| H | 0.643689 | -2.011870 | 0.865207 |
| H | -3.087335 | -7.108564 | 0.210189 |
| H | -4.482955 | -4.992909 | 1.382688 |
| H | -3.852176 | -2.643875 | 2.038663 |
| H | -2.200359 | -2.043874 | 1.675083 |
| H | -3.461427 | -2.016334 | 0.401568 |
| H | -4.354713 | 0.224176 | -0.727915 |
| H | -5.359401 | 1.529913 | 1.149804 |
| H | -3.880404 | 2.690666 | 2.787352 |
| H | -1.402568 | 2.561771 | 2.544919 |
| H | -0.403937 | 1.264986 | 0.666479 |
| H | -0.856163 | -6.085782 | -0.487151 |


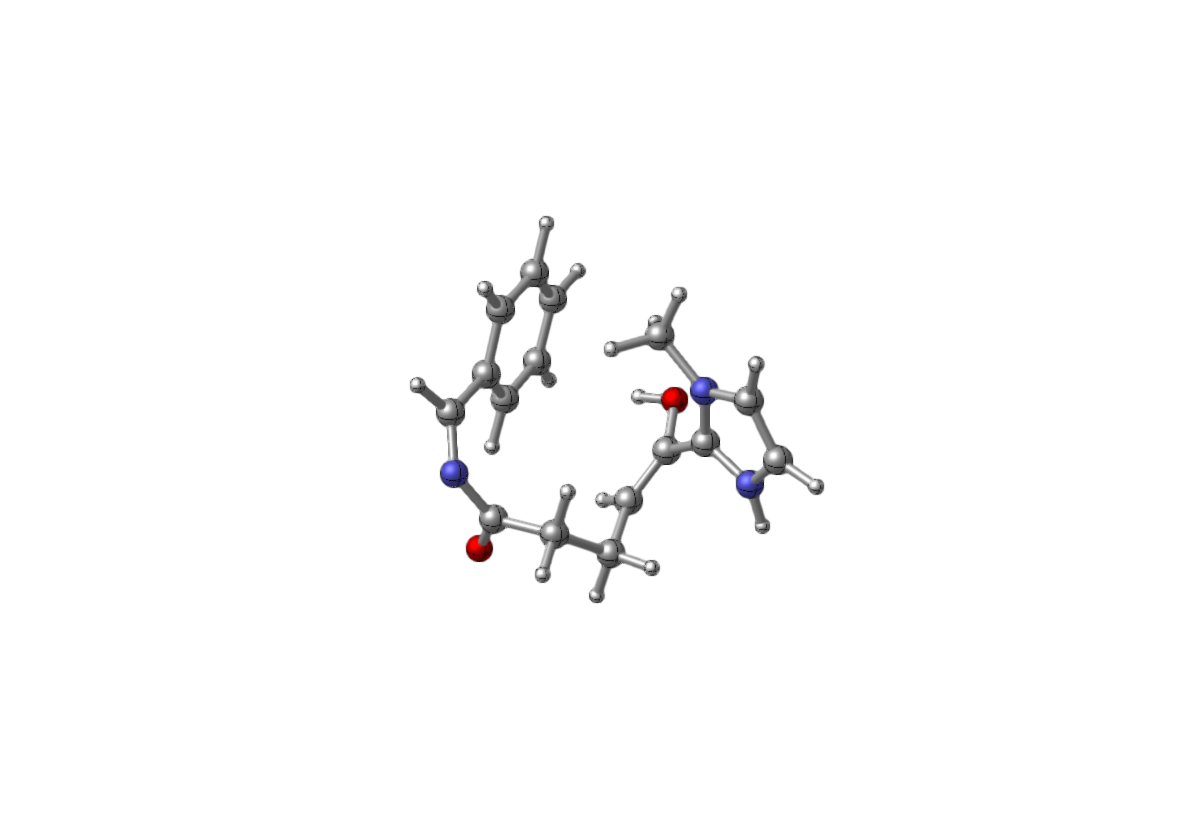


Zero-point correction= 0.320878 (Hartree/Particle)

Thermal correction to Energy= 0.340462

Thermal correction to Enthalpy= 0.341406

Thermal correction to Gibbs Free Energy= 0.269878

Sum of electronic and zero-point Energies= -934.123871

Sum of electronic and thermal Energies= -934.104287

Sum of electronic and thermal Enthalpies= -934.103342

Sum of electronic and thermal Free Energies= -934.174870

**^3-1^MECP (non-protonated)**

E(scf) = -933.964520633 a.u

.ν_min_ = 25.5387 cm^-1^

| C | -1.136522 | -3.380439 | -3.322602 |
| --- | --- | --- | --- |
| C | -1.860378 | -2.044205 | -3.374907 |
| N | -1.798654 | -1.478646 | -2.095093 |
| C | -1.006377 | -2.322181 | -1.149966 |
| C | -0.210010 | -3.228134 | -2.120782 |
| O | -2.398382 | -1.519559 | -4.323852 |
| C | -2.148768 | -0.152543 | -1.898562 |
| C | -0.170091 | -1.493965 | -0.243429 |
| C | 0.049627 | -1.873549 | 1.100724 |
| O | 0.422611 | -0.407551 | -0.763420 |
| N | -0.358731 | -3.048535 | 1.610762 |
| C | 0.002426 | -3.032453 | 2.918591 |
| C | 0.638373 | -1.841313 | 3.232700 |
| N | 0.671124 | -1.104839 | 2.070414 |
| C | 1.123910 | 0.270299 | 1.946753 |
| C | -2.471746 | 0.415942 | -0.641366 |
| C | -2.627846 | -0.354276 | 0.547965 |
| C | -2.783612 | 0.271197 | 1.788214 |
| C | -2.819055 | 1.661372 | 1.886729 |
| C | -2.728394 | 2.440386 | 0.713283 |
| C | -2.563853 | 1.837928 | -0.519788 |
| H | -1.883963 | -4.180102 | -3.178711 |
| H | -0.627031 | -3.569777 | -4.276729 |
| H | -1.671398 | -2.947295 | -0.537779 |
| H | 0.711853 | -2.703860 | -2.415548 |
| H | 0.067457 | -4.166989 | -1.624798 |
| H | -2.276836 | 0.408879 | -2.825959 |
| H | -0.230226 | 0.048286 | -1.358187 |
| H | -0.201879 | -3.870148 | 3.584840 |
| H | 1.061346 | -1.463831 | 4.160048 |
| H | 1.502224 | 0.601041 | 2.922702 |
| H | 1.921378 | 0.356014 | 1.196777 |
| H | 0.290885 | 0.921693 | 1.644591 |
| H | -2.707939 | -1.436538 | 0.492924 |
| H | -2.893518 | -0.345762 | 2.683399 |
| H | -2.943397 | 2.144986 | 2.858157 |
| H | -2.787644 | 3.529987 | 0.779336 |
| H | -2.475607 | 2.447851 | -1.422733 |

|  |  |  |  |
| --- | --- | --- | --- |
|  |  |  |  |


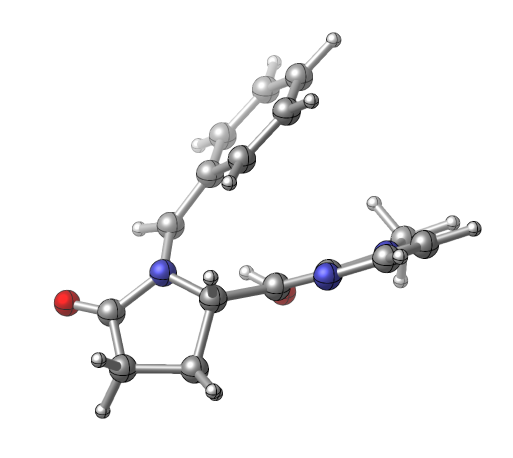


Zero-point correction= 0.306871 (Hartree/Particle)

Thermal correction to Energy= 0.325149

Thermal correction to Enthalpy= 0.326093

Thermal correction to Gibbs Free Energy= 0.259592

Sum of electronic and zero-point Energies= -933.657650

Sum of electronic and thermal Energies= -933.639372

Sum of electronic and thermal Enthalpies= -933.638427

Sum of electronic and thermal Free Energies= -933.704929

# NMR Spectra

^1^H NMR spectrum of **1a**

^13^C NMR spectrum of **1a**

^1^H NMR spectrum of **1b**

^13^C NMR spectrum of **1b**

^1^H NMR spectrum of **1c**

^13^C NMR spectrum of **1c**

^1^H NMR spectrum of **1d**

^13^C NMR spectrum of **1d**

^1^H NMR spectrum of **1e**

^13^C NMR spectrum of **1e**

^1^H NMR spectrum of **1f**

^13^C NMR spectrum of **1f**

^1^H NMR spectrum of **1g**

^13^C NMR spectrum of **1g**

^1^H NMR spectrum of **1h**

^13^C NMR spectrum of **1h**

^1^H NMR spectrum of **1i**

^13^C NMR spectrum of **1i**

^1^H NMR spectrum of **1j**

^13^C NMR spectrum of **1j**

^1^H NMR spectrum of **1k**

^13^C NMR spectrum of **1k**

^1^H NMR spectrum of **1l**

^13^C NMR spectrum of **1l**

^1^H NMR spectrum of **1m**

^13^C NMR spectrum of **1m**

^1^H NMR spectrum of **1n**

^13^C NMR spectrum of **1n**

^1^H NMR spectrum of **1o**

^13^C NMR spectrum of **1o**

^1^H NMR spectrum of **1p**

^13^C NMR spectrum of **1p**

^1^H NMR spectrum of **1q**

^13^C NMR spectrum of **1q**

^1^H NMR spectrum of **1r**

^13^C NMR spectrum of **1r**

^1^H NMR spectrum of **1a-d_7_**

^13^C NMR spectrum of **1a-d_7_**

^1^H NMR spectrum of **2a**

^13^C NMR spectrum of **2a**

^1^H NMR spectrum of **2b**

^13^C NMR spectrum of **2b**

^1^H NMR spectrum of **2c**

^13^C NMR spectrum of **2c**

^1^H NMR spectrum of **cis-2d**

^13^C NMR spectrum of **cis-2d**

^1^H NMR spectrum of ***trans*-2d**

^13^C NMR spectrum of **trans-2d**

^1^H NMR spectrum of **2e**

^13^C NMR spectrum of **2e**

^1^H NMR spectrum of **2f**

^13^C NMR spectrum of **2f**

^1^H NMR spectrum of **2g**

^13^C NMR spectrum of **2g**

^1^H NMR spectrum of **2h**

^13^C NMR spectrum of **2h**

^1^H NMR spectrum of **2i**

^13^C NMR spectrum of **2i**

^1^H NMR spectrum of **2j**

^13^C NMR spectrum of **2j**

^1^H NMR spectrum of **2k**

^13^C NMR spectrum of **2j**

^1^H NMR spectrum of **2l**

^13^C NMR spectrum of **2l**

^1^H NMR spectrum of **2m**

^13^C NMR spectrum of **2m**

^1^H NMR spectrum of **2n**

^13^C NMR spectrum of **2n**

^1^H NMR spectrum of **2o**

^13^C NMR spectrum of **2o**

^1^H NMR spectrum of **2p**

^13^C NMR spectrum of **2p**

^1^H NMR spectrum of **2q**

^13^C NMR spectrum of **2q**

^1^H NMR spectrum of **2r**

^13^C NMR spectrum of **2r**

^1^H NMR spectrum of **3**

^13^C NMR spectrum of **3**

^1^H NMR spectrum of **4**

^13^C NMR spectrum of **4**

^1^H NMR spectrum of **5**

z

^13^C NMR spectrum of **5**

# HPLC Spectra

The HPLC Spectra of *cis*-rac-**2d**

| Peak# | Ret. Time | Area | Height | Peak Start | Peak End | Area% |
| --- | --- | --- | --- | --- | --- | --- |
| 1 | 33.380 | 1791144 | 23702 | 31.975 | 37.000 | 50.8364 |
| 2 | 46.868 | 1732204 | 17851 | 44.908 | 50.058 | 49.1636 |

The HPLC Spectra of *cis-(5S,6R)*-**2d**

| Peak# | Ret. Time | Area | Height | Peak Start | Peak End | Area% |
| --- | --- | --- | --- | --- | --- | --- |
| 1 | 32.759 | 50654589 | 684219 | 31.175 | 35.925 | 94.2268 |
| 2 | 46.693 | 3103582 | 38159 | 45.633 | 48.217 | 5.7732 |

The HPLC Spectra of *cis-(5R,6S)*-**2d**

| Peak# | Ret. Time | Area | Height | Area% |
| --- | --- | --- | --- | --- |
| 1 | 33.180 | 2263902 | 38632 | 4.6030 |
| 2 | 46.250 | 46919583 | 462144 | 95.3970 |

The HPLC Spectra of *trans*-rac-**2d**

| Peak# | Ret. Time | Area | Height | Peak Start | Peak End | Area% |
| --- | --- | --- | --- | --- | --- | --- |
| 1 | 27.922 | 74036 | 1304 | 26.883 | 29.275 | 51.1338 |
| 2 | 35.183 | 70753 | 1006 | 34.042 | 36.825 | 48.8662 |

The HPLC Spectra of *trans*-(*5R,6R*)-**2d**

| Peak# | Ret. Time | Area | Height | Peak Start | Peak End | Area% |
| --- | --- | --- | --- | --- | --- | --- |
| 1 | 27.861 | 436412 | 7517 | 26.675 | 29.900 | 94.3356 |
| 2 | 35.155 | 26204 | 431 | 34.333 | 36.258 | 5.6644 |

The HPLC Spectra of *trans*-(*5S,6S*)-**2d**

| Peak# | Ret. Time | Area | Height | Peak Start | Peak End | Area% |
| --- | --- | --- | --- | --- | --- | --- |
| 1 | 27.942 | 25569 | 526 | 27.225 | 28.758 | 5.2237 |
| 2 | 35.131 | 463909 | 6351 | 33.692 | 37.375 | 94.7763 |

The HPLC Spectra of *cis*-*rac*-**2s**

| Peak# | Ret. Time | Area | Height | Peak Start | Peak End | Area% |
| --- | --- | --- | --- | --- | --- | --- |
| 1 | 35.734 | 4519247 | 53472 | 33.917 | 39.950 | 50.2047 |
| 2 | 52.378 | 4482387 | 40219 | 50.175 | 56.392 | 49.7953 |

The HPLC Spectra of *cis*-*(5S,6R)*-**2s**

| Peak# | Ret. Time | Area | Height | Peak Start | Peak End | Area% |
| --- | --- | --- | --- | --- | --- | --- |
| 1 | 35.632 | 6633982 | 80297 | 34.192 | 39.683 | 86.7392 |
| 2 | 52.540 | 1014208 | 9924 | 51.008 | 54.542 | 13.2608 |

# References

[72] J. L. Zhu, C. R. Schull, A. T. Tam, Á. Rentería-Gómez, A. R. Gogoi, O. Gutierrez, K. A. Scheidt, Photoinduced Acylations Via Azolium-Promoted Intermolecular Hydrogen Atom Transfer, *J. Am. Chem. Soc.* **2023**, *145*, 1535-1541.

[73] C. Lee, W. Yang, R. G. Parr, Development of the Colle-Salvetti correlation-energy formula into a functional of the electron density, *Phys. Rev. B* **1988**, *37*, 785.

[74] A. Becke, **1993**.

[75] S. Grimme, Accurate description of van der Waals complexes by density functional theory including empirical corrections, *J. Comput. Chem.* **2004**, *25*, 1463-1473.

[76] S. Grimme, J. Antony, S. Ehrlich, H. Krieg, A consistent and accurate ab initio parametrization of density functional dispersion correction (DFT-D) for the 94 elements H-Pu, *J. Chem. Phys.* **2010**, *132*, 154104.

[77] S. Grimme, Density functional theory with London dispersion corrections, *WIRES Comput. Mol. Sci.* **2011**, *1*, 211-228.

[78] S. Ehrlich, J. Moellmann, S. Grimme, Dispersion-corrected density functional theory for aromatic interactions in complex systems, *Acc. Chem. Res.* **2013**, *46*, 916-926.

[79] F. Weigend, R. Ahlrichs, Balanced basis sets of split valence, triple zeta valence and quadruple zeta valence quality for H to Rn: Design and assessment of accuracy, *Phys. Chem. Chem. Phys.* **2005**, *7*, 3297-3305.

[80] F. Weigend, Accurate Coulomb-fitting basis sets for H to Rn, *Phys. Chem. Chem. Phys.* **2006**, *8*, 1057-1065.

[81] A. Klamt, G. Schüürmann, COSMO: a new approach to dielectric screening in solvents with explicit expressions for the screening energy and its gradient, *J. Chem. Soc., Perkin Trans. 2* **1993**, 799-805.

[82] J. Tomasi, M. Persico, Molecular interactions in solution: an overview of methods based on continuous distributions of the solvent, *Chem. Rev.* **1994**, *94*, 2027-2094.

[83] J. Andzelm, C. Kölmel, A. Klamt, Incorporation of solvent effects into density functional calculations of molecular energies and geometries, *J. Chem. Phys.* **1995**, *103*, 9312-9320.

[84] V. Barone, M. Cossi, Quantum calculation of molecular energies and energy gradients in solution by a conductor solvent model, *J. Phys. Chem. A* **1998**, *102*, 1995-2001.

[85] M. Cossi, N. Rega, G. Scalmani, V. Barone, Energies, structures, and electronic properties of molecules in solution with the C‐PCM solvation model, *J. Comput. Chem.* **2003**, *24*, 669-681.

[86] M. e. Frisch, G. Trucks, H. Schlegel, G. Scuseria, M. Robb, J. Cheeseman, G. Scalmani, V. Barone, G. Petersson, H. Nakatsuji, Gaussian, Inc. Wallingford, CT, **2016**.

[87] C. Legault, CYLview, 1.0 b, *Université de Sherbrooke* **2009**, *436*, 437.

[88] T. Lu, F. Chen, Multiwfn: A multifunctional wavefunction analyzer, *J. Comput. Chem.* **2012**, *33*, 580-592.

[89] W. Humphrey, A. Dalke, K. Schulten, VMD: visual molecular dynamics, *J. Mol. Graphics* **1996**, *14*, 33-38.

[90] Q. Peng, A. R. Gogoi, Á. Rentería-Gómez, O. Gutierrez, K. A. Scheidt, Visible-light-induced coupling of carboxylic acids with alcohols/amines via a phosphorous linchpin strategy, *Chem* **2023**, *9*, 1983-1993.
